# Biomechanical comparison of different suture anchors used in rotator cuff repair surgery–all-suture anchors are equivalent to other suture anchors: a systematic review and network meta-analysis

**DOI:** 10.1186/s40634-023-00608-w

**Published:** 2023-04-17

**Authors:** Yi-Shiuan Yang, Chien-An Shih, Ching-Ju Fang, Tzu-Teng Huang, Kai-Lan Hsu, Fa-Chuan Kuan, Wei-Ren Su, Chih-Kai Hong

**Affiliations:** 1 Department of Medicine, College of Medicine, National Cheng Kung University, Tainan, Taiwan; 2 Department of Orthopaedic Surgery, National Cheng Kung University Hospital, College of Medicine, National Cheng Kung University, Tainan, Taiwan; 3 Department of Secretariat, National Cheng Kung University Hospital, College of Medicine, National Cheng Kung University, Tainan, Taiwan; 4 Medical Library, National Cheng Kung University, Tainan, Taiwan; 5 Skeleton Materials and Bio-Compatibility Core Lab, Research Center of Clinical Medicine, National Cheng Kung University Hospital, College of Medicine, National Cheng Kung University, Tainan, Taiwan; 6 Musculoskeletal Research Center, Innovation Headquarter, National Cheng Kung University, Tainan, Taiwan

**Keywords:** Rotator cuff injuries, Rotator cuff repair, Suture anchors, All-suture anchors, Biocomposite anchors, PEEK anchor, Metal anchors

## Abstract

**Purpose:**

Suture anchors are commonly used to repair rotator cuff tendons in arthroscopy surgery, and several anchor materials have been created to maximize pull-out strength and minimize iatrogenic damage. We hypothesized that all-suture anchors have biomechanical properties equivalent to those of conventional anchors. Our purpose is to compare the biomechanical properties of different anchors used for rotator cuff repair.

**Methods:**

The Embase, PubMed, Cochrane, and Scopus databases were searched for biomechanical studies on various suture anchors. The search keywords included rotator cuff tears and suture anchors, and two authors conducted study a selection, risk of bias assessment, and data extraction. The failure load, stiffness, and displacement were calculated using the mean differences with 95% confidence intervals (CIs). Failure modes were estimated using summary odds ratios with 95% CIs. The surface under the cumulative ranking curve was used for the relative ranking probabilities. A sensitivity analysis was performed by excluding studies using synthetic bones.

**Results:**

The polyetheretherketone (PEEK) (*p* < 0.001) and all-suture anchors (*p* < 0.001) had higher failure loads than the biocomposite anchors, whereas no significant difference was observed in stiffness among the anchors. The all-suture (*p* = 0.006) and biocomposite anchors (*p* < 0.001) had displacements higher than the metal anchors. The relative ranking of the included anchors in failure loads and displacement changed in sensitivity analysis. The meta-analysis did not find significant differences, but the relative ranking probabilities suggested that all-suture anchor had a higher rate of anchor pull-out and a lower rate of eyelet or suture breakage. In contrast, the metal anchors were associated with a higher number of eyelet breakage episodes.

**Conclusions:**

All-suture anchors showed significantly higher failure loads than the biocomposite anchors and similar cyclic displacements to the biocomposite and PEEK anchors. There were no significant differences in stiffness between all-suture and conventional suture anchors. The relative ranking of biomechanical properties changed in sensitivity analysis, suggesting the potential effect of bone marrow density.

**Level of Evidence:**

Level IV.

**Supplementary Information:**

The online version contains supplementary material available at 10.1186/s40634-023-00608-w.

## Background

Arthroscopic rotator cuff repair has gained popularity for treating rotator cuff injuries, and suture anchors are commonly used to repair rotator cuff tendons in arthroscopic surgery [10]. Several anchor materials have been created to maximize pull-out strength and minimize iatrogenic damage. The anchor composition varied from metal to bioabsorbable to polyetheretherketone (PEEK) and all-suture anchors [7].

Metal anchors are simple to use and easy to visualize radiographically; however, they are associated with possible suture breakage because of sharp anchor eyelets, interference with magnetic resonance imaging, and difficulty in revision surgery [5]. Biocomposite anchors, composed of several materials such as polyglyconate, poly L-lactic acid (PLA), and calcium triphosphate, are associated with less suture damage but may cause inflammatory reactions and cyst formation [2]. The benefits of PEEK anchors include the fact that they appear to be radiolucent, non-absorbable, and non-metallic; however, their pull-out strengths are similar to those of metal anchors [6]. All-suture anchors use expanding intracortical sutures to fix the anchor, allowing for smaller drill holes with less bone disruption [25].

Several studies have compared the biomechanical properties of different materials of suture anchors in rotator cuff repair models [3, 23–25, 28, 31, 40]. However, there is a lack of systematic reviews and meta-analyses that draw a consensus on the optimal choice of suture anchors for rotator cuff repair in terms of biomechanical properties. Although the findings from biomechanical studies cannot be directly applied in clinical practice, precise suggestions from biomechanical studies would aid clinical decision-making in selecting suture anchors.

This network meta-analysis (NMA) aimed to compare the biomechanical properties of different suture anchors used in rotator cuff repair surgery. We hypothesized that all-suture anchors have biomechanical properties equivalent to those of conventional suture anchors.

## Materials and methods

### Search strategy and selection criteria

This NMA was performed following the preferred reporting items for systematic reviews and meta-analyses (PRISMA) extension guidelines [18] and was registered in PROSPERO (registration number: CRD42022337552). We conducted an electronic literature search using the following keywords and medical subject headings: population, rotator cuff tear, rotator cuff repair, rotator cuff injury, rotator cuff disease, and rotator cuff arthropathy. Interventions included all suture anchors, full suture anchors, soft anchors, suture anchors, Q-Fix, Iconix, JuggerKnot, and Y-knot for studies published from the inception of the databases (Embase, PubMed, Cochrane, and Scopus) to April 23, 2022. Additionally, we screened the reference lists of the extracted papers to identify potential studies that were not captured by the electronic database searches. The detailed syntax of the searches can be found in Appendix 3.2.

The inclusion criteria were as follows: (1) studies on rotator cuff repair models using human cadaveric or synthetic specimens; (2) studies that compared different suture anchor materials; (3) randomized controlled or comparative studies; (4) studies published in English; and (5) studies that used cadaver or osteoporotic bone models. The intervention arms included four suture anchor types: all-suture, biocomposite suture, PEEK, and metal anchors.

The exclusion criteria were as follows: (1) non-biomechanical studies, single-arm biomechanical studies, single-arm clinical studies, case series or reports, conference abstracts, or comments on other studies; (2) unknown target outcomes of interest; (3) rotator cuff repair models using pediatric, pathological, and animal specimens; and (4) comparisons without different suture anchor materials. In cases of duplicated data (e.g., different articles based on similar sources of participants), we included studies with more biomechanical outcomes.

### Data extraction and quality assessment

Two authors (Y-S Y and C-A S) initially screened titles and abstracts based on the inclusion and exclusion criteria and then independently evaluated the risk of bias for each domain described in the Cochrane risk-of-bias tool [16]. The tool includes five domains: the randomization process, intended intervention deviations, missing outcome data, outcome measurement, and reported result selection. Each study was identified as “high-risk,” “some concerns,” or “low-risk.” When a consensus could not be reached, a third author (C-K H) resolved any disagreements. The Quality Appraisal for Cadaveric Studies (QUACS) scale was used to assess the included studies [39].

One author (C-K H) extracted the following data and information: (1) first author’s name and publication year; (2) study nation or area; (3) study design; (4) specimen characteristics; (5) intervention and control protocols; and (6) primary biomechanical outcome measurement, including load-to-failure, stiffness, displacement, and failure modes.

### Parameter selection

When several stiffness and cyclic displacement values were estimated in a study, the calculated load for stiffness and cyclic displacement (from 10 to 100 N) and the cycle for displacement were chosen for cyclic-to-failure as priorities, followed by the most common cycles (100 and 1000 cycles). These loads and cycles reflected the stiffness and displacement values most frequently measured in other biomechanical studies. Displacements from different studies with variations were calculated as a single outcome, “displacement.” All authors validated the accuracy of the extracted data. When necessary, the authors of the original article were contacted to retrieve any missing information or additional data.

### Data synthesis and analysis

All statistical analyses were performed using the Stata 15.0 software (StataCorp. 2017. Stata Statistical Software: Release 15. College Station, TX: StataCorp LLC.). As only a few studies were included, various diameters and shapes of suture anchors were combined to calculate the results. For load-to-failure, stiffness, and displacement, mean differences (MDs) were calculated at 95% confidence intervals (CIs). For failure-mode data, we estimated summary odds ratios (ORs) with 95% CIs. An OR value of < 1 indicated a higher incidence of suture anchor failure. A pairwise meta-analysis was conducted for direct comparisons between trials, and an NMA was carried out to combine direct and indirect evidence [37]. The heterogeneity of the sample size and intervention protocols was evaluated using the estimated standard deviation of the effects across these studies. Statistical significance was defined as a two-tailed *p*-value < 0.05.

The relative ranking probabilities for the interventions and surface under the cumulative ranking curve (SUCRA) were calculated. The larger the SUCRA value [33], the higher the rank of the intervention [9, 29].

Publication bias in the NMA was examined using Egger regression. The potential inconsistency between the direct and indirect comparisons of all studies was determined using the loop-specific approach, local inconsistency with the node-splitting method, and the global inconsistency among the entire NMA with the design-by-treatment model [13, 38]. Finally, a sensitivity analysis was performed after excluding trials conducted using synthetic bone models. We also assessed the presence of small study effects on each outcome using a comparison-adjusted funnel plot. The funnel plot asymmetry indicated a small study effect bias [12, 17].

## Results

### Study selection, description, and quality

**Fig. 1:**
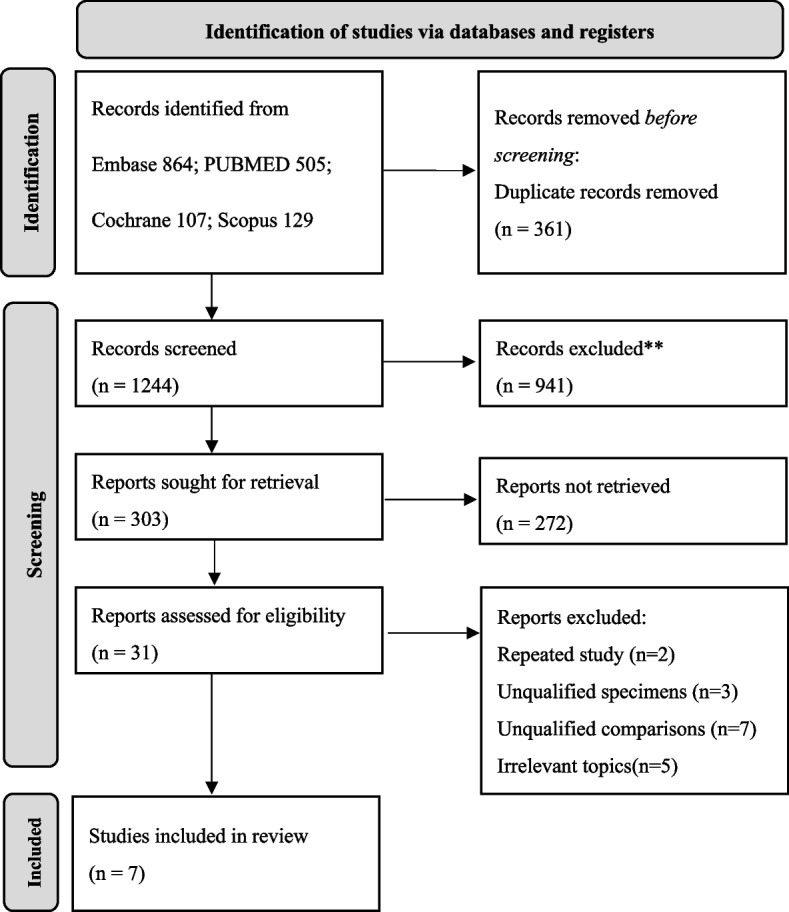
PRISMA Flow diagram for systematic reviews

As shown in the PRISMA flow diagram in Fig. 1, 1601 studies were identified after searching the databases and other sources. We finally retrieved seven studies included in the NMA [3, 23–25, 28, 31, 40]. These studies included > 60 sawbones, 98 humeri, and 291 anchors. All seven studies compared load-to-failure, three compared stiffness [24, 25, 31], five compared anchor displacement [23–25, 28, 31], and six compared the incidence of failure modes [3, 23–25][28, 40]. The characteristics of all studies and specimens are summarized in Tables 1 and 2.

**Table 1:** Descriptive characteristics of the included studies

Study and Year	Nation	Specimen, n	Age	Gender	Bone Mineral Density	Insertion Angle	Intervention	Outcomes	Level of Evidence	Risk of Bias
Yamauchi et al., 2022 [40]	Japan	Sawbone model, 160	N/A	N/A	1. 10-pounds/cubic foot (160 mg/cm3) 2. 5-pounds/cubic foot (80 mg/cm3)	90 degrees	Corkscrew FT Ti 4.5 mm, HEALICOIL PK 4.5 mm, Corkscrew Bio 4.75 mm	LTF, Failure mode	IV	High risk
Rosso et al., 2020 [31]	Switzerland	Sawbone model, 60	N/A	N/A	1. Physiological group: 120 mg/cm3 2. Osteoporotic group: 90 mg/cm3	45 degrees	TwinFix Ti 4.5 mm, Healix BR 4.5 mm, Iconix 2.3 mm	LTF, STF, DIS	IV	Low risk
Ntalos et al., 2019 [24]	Germany	Human humerus, 10	50-73y	N/A	1. All-suture anchor: 126 ± 25 mg/cm3 2. Conventional anchor: 127 ± 30 mg/cm3	90 degrees	Y-knot 2.8 mm, CrossFT 4.5 mm	LTF, STF, DIS, Failure mode	IV	Some concerns
Ntalos et al., 2019 [25]	Germany	Human humerus, 36	22-76y (61.4 ± 11y)	N/A	1. 126 ± 18 mg /cm3 2. 126 ± 26 mg /cm3 3. 127 ± 16 mg /cm3	45, 90, 110 degrees	Y-knot 2.8 mm, CrossFT 4.5 mm	LTF, STF, DIS, Failure mode	IV	Low risk
Nagra et al., 2017 [23]	United Kingdom	Human humerus, 24	58-96y	16 M, 8F	N/A	N/A	Y-knot 2.8 mm, TwinFix ultra PK 6.5 mm	LTF, DIS, Failure mode	IV	Some concerns
Barber et al., 2010 [3]	United States	Human humerus, 16	70-96y	7 M, 1F	N/A	N/A	Bio-Corkscrew FT 5.5 mm, CrossFT PK 5.5 mm	LTF, DIS, Failure mode	IV	Some concerns
Pietschmann et al., 2009 [28]	Germany	Human humerus, 12	27-93y	8 M, 4F	1. Non-osteopenic: 109 ± 26 mg/cm3 2. Osteoporotic bones: 41 ± 20 g/cm3	45 degrees	SPIRALOK BC 5.0 mm, Super Revo 5.0 mm	LTF, DIS, Failure mode	IV	Some concerns

LEGEND: Characteristics included author names, publication year, sources of country, specimen type, specimen numbers, age, gender and bone mineral densities of human cadaver, anchor insertion anchor, interventions, outcomes, level of evidence and risk of bias of each study. *LTF* Load to failure, *STF* Stiffness, *DIS* Displacement

**Table 2:** Descriptive characteristics of the included suture anchors

	**Anchor Name**	**Study**	**Material**	**Suture**	**Loaded**	**Diameter**	**n**
All-suture anchor	Iconix	Rosso et al	Braided UHMWP	No. 2 Force Fiber	Single	2.3 mm	10
	Y-knot	Ntalos et al Ntalos et al Nagra et al	Braided UHMWP	No. 2 HiFi	Single	2.8 mm	24
Biocomposite suture anchor	Bio Corkscrew	Yamauchi et al	Poly-L-lactic acid	No. 2 FiberWire	Single	4.75 mm	5
	Bio Corkscrew FT	Barber et al	Poly-L-lactic acid	No. 2 FiberWire	Single	5.5 mm	12
	Healix BR	Rosso et al	30% b-TCP/ 70% PLGA	No. 2 Orthocord	Single	4.5 mm	10
	SPIRALOK BC	Pietschmann et al	Poly-L-lactic acid	USP 2	Single	5.0 mm	6
PEEK suture anchor	CrossFT PK	Ntalos et al Ntalos et al	PEEK	No. 2 HiFi	Single	4.5 mm	19
	CrossFT PK	Barber et al	PEEK	No. 2 HiFi	Single	5.5 mm	12
	HEALICOIL PK	Yamauchi et al	PEEK	No. 2 Ultrabraid	Single	4.5 mm	5
	TwinFix ultra PK	Nagra et al	PEEK	No. 2 white/Cobraid blue	Single	6.5 mm	4
Metal suture anchor	TwinFix Ti	Rosso et al	Titanium	No. 2 white/Cobraid blue	Single	4.5 mm	10
	Corkscrew FT	Yamauchi et al	Titanium	No. 2 FiberWire	Single	4.5 mm	5
	Super Revo	Pietschmann et al	Titanium	No. 2 HiFi	Single	5.0 mm	6

LEGEND: Characteristics included each anchor name, study resource, anchor material, suture material, repair construction (all single loaded), anchor diameter and numbers. *UHMWP* Ultra-high-molecular-weight polyethylene, *PEEK* Polyetheretherketone, *β-TCP* β-tricalcium phosphate, *PLGA* Poly lactic-co-glycolic acid

### Network meta-analysis

**Fig. 2:**
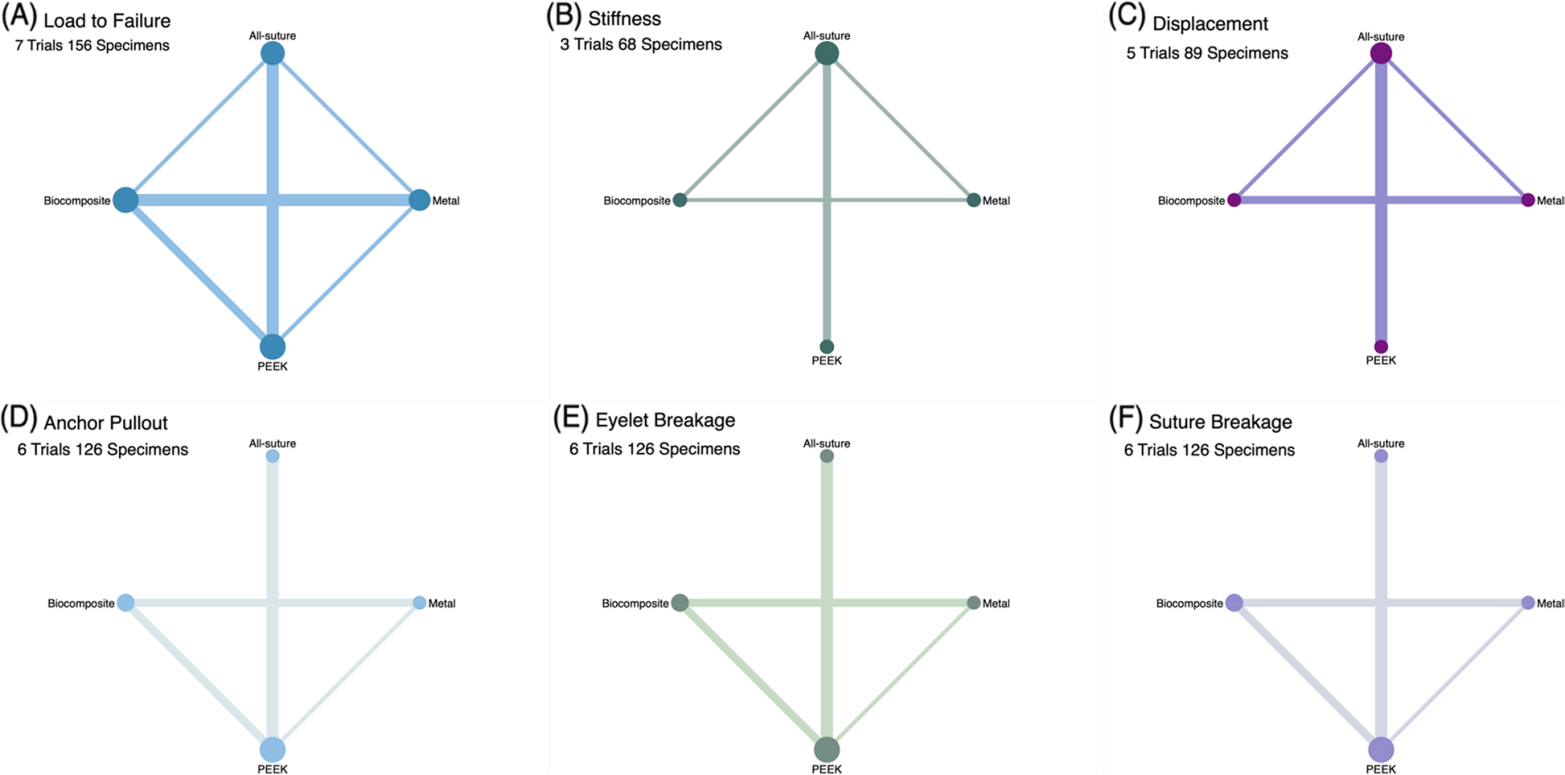
Summary of Network Geometry of Each Biomechanical Property. The size of straight lines is proportional to the number of studies, and the size of round nodes is proportional to the number of interventions. Direct and indirect evidence were combined for multiple treatment comparisons. **A** All included studies reported failure load values; therefore, all anchors had connections with each other. **B** Three studies had stiffness values, and (**C**) five studies had displacement values and a lack of direct connections from PEEK to the biocomposite and metal anchors. (**D**–**F**) Six studies reported failure mode rates and a lack of direct connections from all-suture anchors to biocomposites and metal anchors

**Fig. 3:**
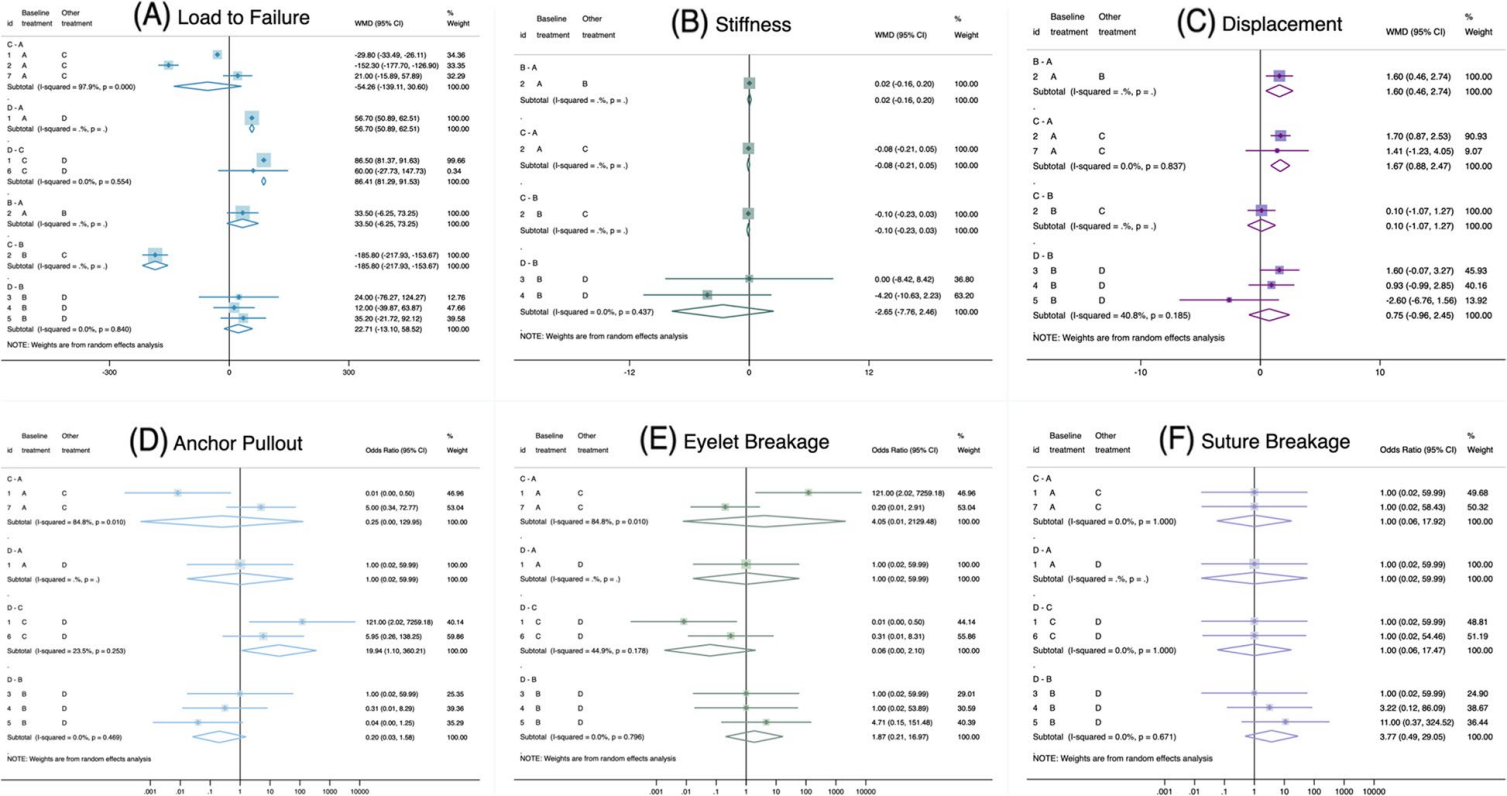
Forest Plots of Each Biomechanical Property. Forest plots demonstrate (A–C) weighted mean differences and (D–F) odds ratios

**Fig. 4:**
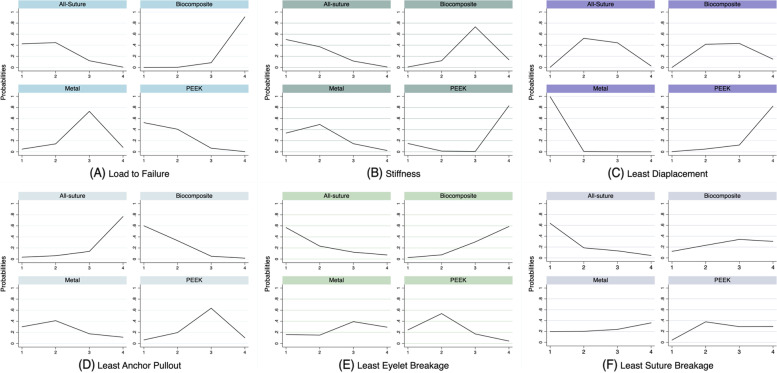
Probability Rankings of Each Biomechanical Property. In (**A**) and (**B**), higher values indicate a superior ranking. **C**-**F** smaller values indicate superior ranking

**Fig. 5:**
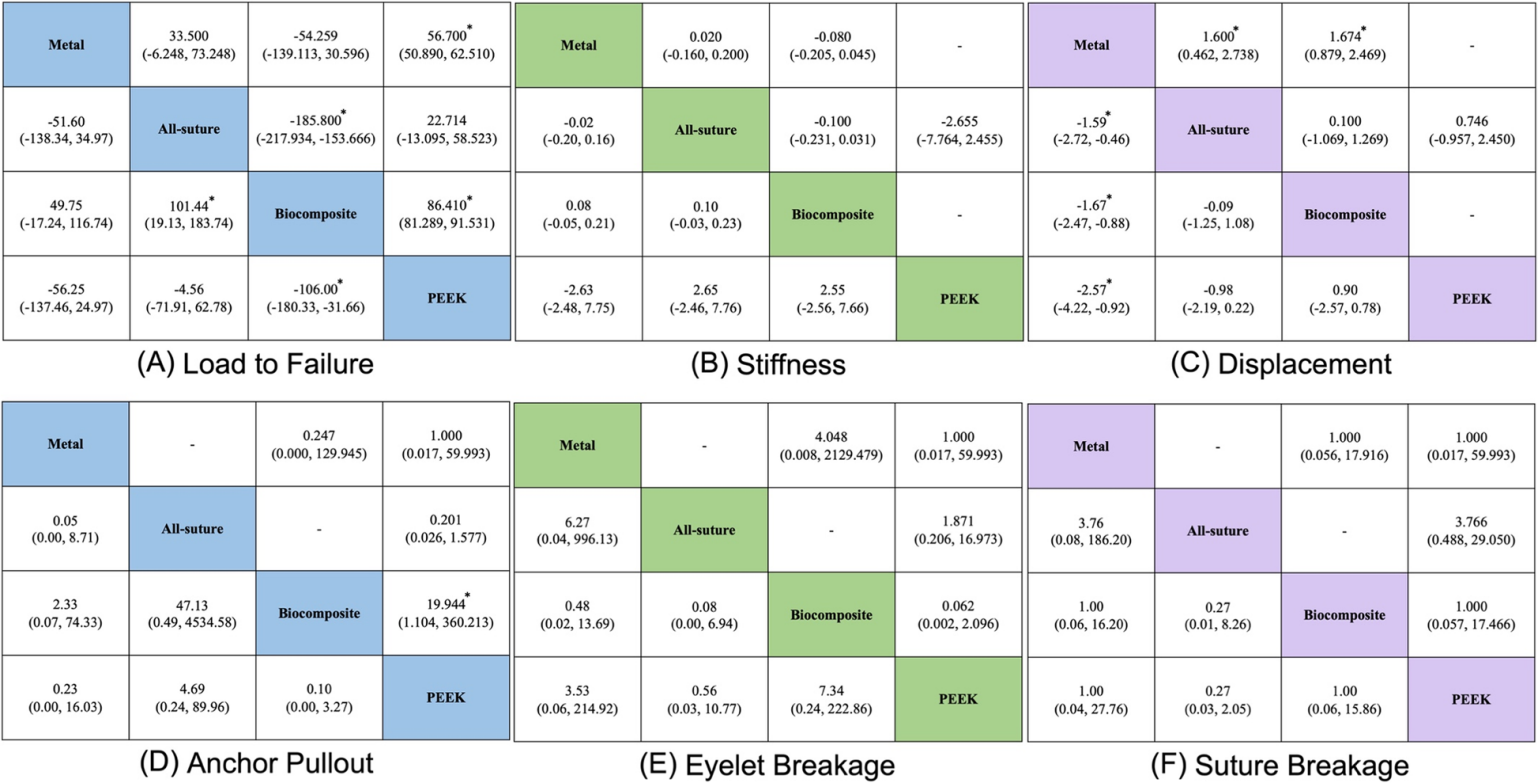
League Table and Pairwise of Each Biomechanical Property. The results of the network meta-analysis are presented in the lower-left half, whereas those of the pairwise meta-analysis are presented in the upper-right half. In (**A**-**C**), the weighted mean differences with 95% confidence intervals (CIs) are presented. If the 95% CIs crossed 0, the differences are not statistically significant. In (**D**-**F**), odds ratios (ORs) with 95% CIs are presented, and the column-defining treatment is favored if the odds ratio is < 1. If the 95% CIs crossed 1, there are no significant difference between groups. The stars marked in the figure refer to significant differences

The network geometry for each outcome is shown in Fig. 2. The forest plots and rank probabilities are shown in Figs. 3 and 4. The league table and pairwise plots of MD and OR with 95% CIs are shown in Fig. 5.

### Load to failure

In total, 128 anchors were evaluated. The PEEK anchor showed significantly higher strength than the metal and biocomposite anchors, while the biocomposite anchor showed significantly lower strength than the all-suture anchor. The PEEK anchor (SUCRA: 81.9%) ranked first ahead of the all-suture anchor (76.5%), followed by the metal anchor (38.7%) and the biocomposite anchor (3.0%) (Fig. 5A). In the sensitivity analysis, the PEEK anchor ranked first (SUCRA: 91.5%), followed by the all-suture (58.0%), biocomposite (39.7%), and metal (10.8%) anchors. However, no significant difference was observed in the sensitivity analysis.

### Stiffness

A total of 68 anchors were evaluated, and no significant differences in stiffness were observed. The all-suture and metal anchors ranked first (SUCRA: 79.1% and 71.5%, respectively), followed by the biocomposite (33.3%) and PEEK (16.1%) anchors (Fig. 5B). However, metal anchors (83.6%) ranked much higher than all-suture anchors (16.4%) in the sensitivity analysis, in which only cadaveric bone models were included.

### Displacement

In total, 89 anchors were evaluated. The all-suture and biocomposite anchors exhibited significantly greater displacements than the metal anchor. The metal anchor ranked first in terms of the least anchor displacement (SUCRA: 99.8%), followed by the all-suture (50.2%), biocomposite (42.3%), and PEEK (7.7%) anchors (Fig. 5C). In the sensitivity analysis, the all-suture anchor ranked first (66.3%) in displacement with the lowest values, followed by the metal (61.0%), biocomposite, and PEEK (35.5%; 37.3%) anchors. However, no significant difference was observed in the sensitivity analysis.

### Failure mode

A total of 98 anchors were evaluated. The PEEK anchor was significantly more likely to be pulled out than the biocomposite anchor. No other significant differences were observed in the three failure modes among the different suture anchors. The all-suture anchors were most probably pulled out, followed by the PEEK, metal, and biocomposite anchors (Fig. 5D). The biocomposite anchors were more likely to cause the eyelet breakage than the other three suture anchors (Fig. 5E). Finally, suture breakage was less likely to occur in all suture anchors (Fig. 5F). However, the differences in three failure modes among four anchors did not reach statistical significances. In the sensitivity analysis, eyelet or suture failure was the least likely to occur with the all-suture anchor. The metal anchor still had the highest possibility of eyelet breakage and the least anchor pull-out.

### Publication Bias, Inconsistency, and Heterogeneity

The funnel and Egger regression plots were mostly symmetric. Significant global and local inconsistencies were observed in the design-by-treatment interaction, side-splitting inconsistency, and loop-inconsistency models. (Additional file: Appendices 10, 11).

### Risk of Bias, CINeMA Assessment, and the QUACS Scale

The overall bias was low risk, and some concerns are listed in an additional file (Appendix 7). The confidence ratings of CINEMA were generally very low (Additional file: Appendix 13). The QUACS ranged from 8–10 out of 13 points (Additional file: Appendix 15).

## Discussion

The most significant finding of this study was that PEEK anchors had the greatest ultimate failure loads, whereas biocomposite anchors had the least; all-suture anchors had the highest stiffness, whereas PEEK anchors had the least. Regarding the displacement ranking, the metal anchor had the least displacement, followed by the all-suture anchor. We also found that the ranking of the anchors included in the ultimate failure load and displacement changed in the sensitivity analysis, which excluded synthetic bone models and included osteoporotic cadaveric models alone.

An ideal suture anchor should have a high failure load, high stiffness, and small cyclic displacement. Theoretically, a suture anchor with a high failure load reduces the possibility of surgical failure. The structural design and materials of anchors may affect the ultimate failure load [4, 5, 8, 19]. PEEK anchors, made of crystalline thermoplastic, are sufficiently solid to build a stable anchor-bone construct [8]. Biocomposite anchors made of PLA or calcium triphosphate can be absorbed into the bone, raising concerns regarding the preservation of the pull-out strength [4, 5]. Because only small drill tunnels are required for all-suture anchors, their surface area-to-anchor volume ratio is significantly higher than that of conventional anchors, providing sufficient stability without extensive bone damage [19].

In addition to the ultimate failure load, stiffness is an important feature when evaluating the biomechanical properties of suture anchors because it represents the capability of a suture anchor to stabilize the repair structure [23]. A repaired structure with greater stiffness improves surgical success and shortens recovery periods [23]. This NMA compared the stiffness of the constructs in rotator cuff repair models and revealed that all-suture anchors had comparable stiffness to other suture anchors. Based on the ultimate failure load and stiffness findings, an all-suture anchor can be an attractive option owing to its superior biomechanical properties. However, care should be taken when interpreting the results, as the sensitivity analysis indicated a high risk of bias because of the small number of studies included.

All-suture anchors have shown a clinical performance equivalent to that of hard-body anchors for rotator cuff repair [27]. Van der Bracht et al. reported a series of 20 patients who underwent double-row cuff repairs using all-suture anchors for both the medial and lateral rows [36]. They found that only one patient sustained a retear and that there was no difference in the contralateral supraspinatus strength at mean postoperative 1.58 years [36]. Dhinsa et al. analyzed 31 patients who underwent double-row repair and reported one retear at a mean follow-up of 10.2 months with a mean Constant score of 77.1 [11]. Ro et al. retrospectively compared 213 patients who underwent single-row rotator cuff repair using all-suture (*n* = 137), bioabsorbable (*n* = 36) or PEEK anchors (*n* = 40). They reported that 71% of the repaired tendons were healed irrespective of the anchor type [30]. Since promising clinical outcomes have been reported [11, 30, 36], the superior biomechanical properties of the current NMA provide further support for using all-suture anchors in rotator cuff repair.

Anchor characteristics are closely related to failure modes [8, 15, 22, 26]. This study showed that all-suture anchors were much more likely to fail owing to anchor pull-out, biocomposite anchors had more eyelet breakage, and that metal anchors easily caused suture breakage. The edge of the metal anchor is sharp enough to easily cut the suture, leading to frequent suture breakage [15, 22, 26]. The eyelet design of biocomposite anchors commonly uses a distal crossbar structurally weaker than the screw threads, resulting in crossbar breaking as the predominant anchor failure type [8]. PEEK anchors are chemically resistant without sharp edges, decreasing suture or eyelet breakage rates [8]. Thus, the PEEK anchors are more likely to be pulled out by the entire repair structure. Instead of the eyelet or suture breakage, anchor pull-out is the most common failure mode for all-suture anchors.

Bone mineral density affects the healing of the rotator cuff tendon [1] and suture anchor fixation strength [14, 21, 32, 34]. Placing anchors at areas with good cortical density provides higher resistance to pulling strength, thereby preventing suture anchor loosening and ensuring successful repair [14, 34]. Although previous studies have reported decreased load-to-failure in specimens with lower bone mineral density [20, 34, 35], there is a lack of comparison of the fixation strengths of different anchor types with respect to bone marrow density. This study conducted a sensitivity analysis, representing findings obtained from osteoporotic cadaveric models alone. The results showed that the ranking of the included anchors in terms of ultimate failure load and displacement changed after sensitivity analysis, suggesting that the biomechanical performance of different suture anchors is affected by bone marrow density to varying degrees. Further studies must analyze the effect of bone marrow density on the fixation strength of different anchor types.

### Limitations

This study had several limitations. First, some studies that used human cadavers and synthetic bone models were included in the NMA. Because the number of human cadaver studies that met the inclusion criteria was limited [3, 23–25, 28], we also included studies that used synthetic bone models [31, 40]. The inconsistent bone mineral densities observed in different studies may have affected the results. Although this study conducted a sensitivity analysis, thereby providing results from cadaveric studies only, diversity in bone mineral density was observed in these studies, potentially influencing the study results. The detailed mean and standard deviations of bone mineral density in each groups can be found in Appendix 6.4. Second, the anchors were classified according to the materials of which they were made, and their size and shape could not be controlled. Thus, inter-anchor variability within the same group may have caused these inconsistencies. Finally, the absorbability of the suture anchors was not considered in the time-zero biomechanical studies. Therefore, care should be taken when applying our biomechanical findings to clinical practice, as fixation strength may change after surgery. Despite these limitations, the NMA compared the most common biomechanical outcomes of different suture anchors, which may predict the possible clinical outcomes of patients.

## Conclusions

All-suture anchors showed significantly higher failure loads than the biocomposite anchors and similar cyclic displacements to the biocomposite and PEEK anchors. There were no significant differences in stiffness between all-suture and conventional suture anchors. The relative ranking of biomechanical properties changed in sensitivity analysis, suggesting the potential effect of bone marrow density.

## Supplementary Information

**Additional file 1. **

## Abbreviations

NMA: Network meta-analysis
MD: Mean difference
CI: Confidence interval
OR: Odds ratio
SUCRA: The surface under the cumulative ranking curve
PEEK: Polyetheretherketone
PLA: Poly L-lactic acid
