# Supplementary material for: Biomechanical comparison of different suture anchors used in rotator cuff repair surgery–all-suture anchors are equivalent to other suture anchors: a systematic review and network meta-analysis
Source: J Exp Orthop. 2023 Apr 17;10:45. doi: 10.1186/s40634-023-00608-w (PMC10110812; doi:10.1186/s40634-023-00608-w)
Supplement: Supplementary file 1 — Additional file 1. [file 40634_2023_608_MOESM1_ESM.docx]

**Appendix**

**Table of content**

[Appendix 1. Systematic reviews and meta-analysis published on materials of suture anchors in rotator cuff repairs 1](#_Toc127831181)

[Appendix 2 : PRISMA checklist 2](#_Toc127831182)

[Appendix 3: Protocol and Search strategies 6](#_Toc127831183)

[3.1. Review eligibility criteria 6](#_Toc127831184)

[3.2. Search vocabulary 7](#_Toc127831185)

[Appendix 4: Excluded studies and reasons 8](#_Toc127831186)

[Appendix 5: List of included studies 11](#_Toc127831187)

[5.1. Load to Failure 11](#_Toc127831188)

[5.1.1. Study group and primary outcome 11](#_Toc127831189)

[5.1.2. Study population and enrollment criteria 11](#_Toc127831190)

[5.1.3. Baseline characteristics 14](#_Toc127831191)

[5.2. Stiffness 16](#_Toc127831192)

[5.2.1. Study group and primary outcome 16](#_Toc127831193)

[5.2.2. Study population and enrollment criteria 16](#_Toc127831194)

[5.2.3. Baseline characteristics 17](#_Toc127831195)

[5.3. Displacement 18](#_Toc127831196)

[5.3.1. Study group and primary outcome 18](#_Toc127831197)

[5.3.2. Study population and enrollment criteria 18](#_Toc127831198)

[5.3.3. Baseline characteristics 20](#_Toc127831199)

[5.4. Failure mode 21](#_Toc127831200)

[5.4.1. Study group and primary outcome 21](#_Toc127831201)

[5.4.2. Study population and enrollment criteria 21](#_Toc127831202)

[5.4.3. Baseline characteristics 23](#_Toc127831203)

[5.5. References 26](#_Toc127831204)

[Appendix 6: Assessment of transitivity 27](#_Toc127831205)

[6.1. Anchor Numbers 27](#_Toc127831206)

[6.2. Human Cadaver: Synthetic bone rate 28](#_Toc127831207)

[6.3. Anchor Insertion Angle 29](#_Toc127831208)

[6.4. Bone Mineral Density 30](#_Toc127831209)

[6.5. Age of Human cadaver 30](#_Toc127831210)

[6.6. Publication Year 30](#_Toc127831211)

[Appendix 7: Risk of bias 31](#_Toc127831212)

[7.1. Load to Failure 31](#_Toc127831213)

[7.1.1. Risk of bias assessment for the individual domains: Load to Failure 31](#_Toc127831214)

[7.1.2. Risk of bias assessment for the individual studies: Load to Failure 31](#_Toc127831215)

[7.1.3. Risk of bias notes for the individual studies: Load to Failure 32](#_Toc127831216)

[7.2. Stiffness 33](#_Toc127831217)

[7.2.1. Risk of bias assessment for the individual domains: Stiffness 33](#_Toc127831218)

[7.2.2. Risk of bias assessment for the individual studies: Stiffness 33](#_Toc127831219)

[7.2.3. Risk of bias notes for the individual studies: Stiffness 34](#_Toc127831220)

[7.3. Displacement 34](#_Toc127831221)

[7.3.1. Risk of bias assessment for the individual domains: Displacement 34](#_Toc127831222)

[7.3.2. Risk of bias assessment for the individual studies: Displacement 34](#_Toc127831223)

[7.3.3. Risk of bias notes for the individual studies: Displacement 35](#_Toc127831224)

[7.4. Failure modes 35](#_Toc127831225)

[7.4.1. Risk of bias assessment for the individual domains in Failure mode 35](#_Toc127831226)

[7.4.2. Risk of bias assessment for the individual studies: Failure modes 36](#_Toc127831227)

[7.4.3. Risk of bias notes for the individual studies: Failure modes 36](#_Toc127831228)

[Appendix 8: Results 37](#_Toc127831229)

[8.1. Extracted outcome data in Load to failure 37](#_Toc127831230)

[8.1.1. List of Load to failure in each study 37](#_Toc127831231)

[8.1.2. Values of load to failure in each suture anchor 37](#_Toc127831232)

[8.2. Extracted outcome data in Stiffness 38](#_Toc127831233)

[8.2.1. List of stiffness in each study 38](#_Toc127831234)

[8.2.2. Values of stiffness in each suture anchor 38](#_Toc127831235)

[8.3. Extracted outcome data in Displacement 39](#_Toc127831236)

[8.3.1. List of displacement in each study 39](#_Toc127831237)

[8.3.2. Values of displacement in each suture anchor 39](#_Toc127831238)

[8.4. Extracted outcome data in Failure modes 40](#_Toc127831239)

[8.4.1. Events of anchor pullout in each suture anchor 40](#_Toc127831240)

[8.4.2. Events of eyelet breakage in each suture anchor 40](#_Toc127831241)

[8.4.3. Events of suture breakage in each suture anchor 40](#_Toc127831242)

[Appendix 9: League table, Pairwise and Relative ranking 42](#_Toc127831243)

[9.1. Load to Failure 42](#_Toc127831244)

[9.1.1. Load to Failure in league table and pairwise 42](#_Toc127831245)

[9.1.2. Load to Failure in relative ranking probability 43](#_Toc127831246)

[9.2. Stiffness 44](#_Toc127831247)

[9.2.1. Stiffness in league table and pairwise 44](#_Toc127831248)

[9.2.2. Stiffness in relative ranking probability 45](#_Toc127831249)

[9.3. Displacement 46](#_Toc127831250)

[9.3.1. Displacement in league table and pairwise 46](#_Toc127831251)

[9.3.2. LEAST Displacement in relative ranking probability 47](#_Toc127831252)

[9.4. Failure mode: Anchor pullout 48](#_Toc127831253)

[9.4.1. Anchor pullout in league table and pairwise 48](#_Toc127831254)

[9.4.2. LEAST Anchor pullout in relative ranking probability 49](#_Toc127831255)

[9.5. Failure mode: Eyelet breakage 50](#_Toc127831256)

[9.5.1. Eyelet breakage in league table and pairwise 50](#_Toc127831257)

[9.5.2. LEAST Eyelet breakage in relative ranking probability 51](#_Toc127831258)

[9.6. Failure mode: Suture breakage 52](#_Toc127831259)

[9.6.1. Suture breakage in league table and pairwise 52](#_Toc127831260)

[9.6.2. LEAST Suture breakage in relative ranking probability 53](#_Toc127831261)

[9.7. Interval plot 54](#_Toc127831262)

[Appendix 10: Publication bias 56](#_Toc127831263)

[10.1. Publication bias: Load to Failure 56](#_Toc127831264)

[10.2. Publication bias: Stiffness 57](#_Toc127831265)

[10.3. Publication bias: Displacement 58](#_Toc127831266)

[10.4. Publication bias: Anchor Pullout 59](#_Toc127831267)

[10.5. Publication bias: Eyelet Breakage 60](#_Toc127831268)

[10.6. Publication bias: Suture Breakage 61](#_Toc127831269)

[Appendix 11: Inconsistency 62](#_Toc127831270)

[11.1. Inconsistency: Load to Failure 62](#_Toc127831271)

[11.2. Inconsistency: Stiffness 63](#_Toc127831272)

[11.3. Inconsistency: Displacement 64](#_Toc127831273)

[11.4. Inconsistency: Anchor Pullout 65](#_Toc127831274)

[11.5. Inconsistency: Eyelet Breakage 66](#_Toc127831275)

[11.6. Inconsistency: Suture Breakage 67](#_Toc127831276)

[Appendix 12: Meta-regression 68](#_Toc127831277)

[12.1 SUCRA and mean ranks changes before and after model adjustments: Load to Failure 68](#_Toc127831278)

[12.2 SUCRA and mean ranks changes before and after model adjustments: Stiffness 68](#_Toc127831279)

[12.3 SUCRA and mean ranks changes before and after model adjustments: Displacement 68](#_Toc127831280)

[12.4 SUCRA and mean ranks changes before and after model adjustments: Anchor Pullout 68](#_Toc127831281)

[12.5 SUCRA and mean ranks changes before and after model adjustments: Eyelet Breakage 68](#_Toc127831282)

[12.6 SUCRA and mean ranks changes before and after model adjustments: Suture Breakage 68](#_Toc127831283)

[Appendix 13: Grading the evidence using CINeMA web application 69](#_Toc127831284)

[13.1 Confidence Rating: Load to Failure 69](#_Toc127831285)

[13.2 Confidence Rating: Stiffness 69](#_Toc127831286)

[13.3 Confidence Rating: Displacement 69](#_Toc127831287)

[13.4 Confidence Rating: Failure Modes 70](#_Toc127831288)

[Appendix 14: Sensitivity analysis 71](#_Toc127831289)

[14.1 League table excluding trials of artificial bone: Load to Failure 71](#_Toc127831290)

[14.2 League table excluding trials of artificial bone: Stiffness 71](#_Toc127831291)

[14.3 League table excluding trials of artificial bone: Displacement 72](#_Toc127831292)

[14.4 League table excluding trials of artificial bone: Anchor Pullout 72](#_Toc127831293)

[14.5 League table excluding trials of artificial bone: Eyelet Breakage 73](#_Toc127831294)

[14.6 League table excluding trials of artificial bone: Suture Breakage 73](#_Toc127831295)

[Appendix 15: The Quality Appraisal for Cadaveric Studies (QUACS) scale 74](#_Toc127831296)

[15.1 The Tables of The Quality Appraisal for Cadaveric Studies (QUACS) scale 74](#_Toc127831297)

#

# Appendix 1. Systematic reviews and meta-analysis published on materials of suture anchors in rotator cuff repairs

eTable 1. Systematic reviews and meta-analysis published on materials of suture anchors in rotator cuff repairs

| **Author.**  **Journal, Year** | **No. of trials**  **No. of cases** | **Specimens** | **Interventions**  **(No. of trials)** | **Comparisons**  **(No. of trials)** | **Outcomes** | **Limitationa** |
| --- | --- | --- | --- | --- | --- | --- |
| Ergun, S., et al./ Arthroscopy, Sports Medicine, and Rehabilitation/ 2020 | 16,  >2281 | (1) Rotator cuff repair  (2) Biceps tenodesis  (3) Glenoid | (1) JuggerKnot  (2) Iconix  (3) Y-knot  (4) Q-Fix  (5) Draw Tight | (1) Bio Mini Revo  (2) BioComposite  (3) SutureTak  (4) Bioraptor PEEK  (5) CorkScrew  (6) SwiveLock  (7) PEEK TwinIx Ultra | ASAs showed satisfactory results and little increase in the complication rate | 1. Relatively small number of studies used widely diverse biomechanical measurement methodologies. 2. Investigations were done in various soft tissue structures, such as ligamentous (labral) or tendinous (rotator cuff and biceps tendon) tissues. |

# Appendix 2 : PRISMA checklist

eTable 2. PRISMA checklist

| **Section/Topic** | **Item #** | **Checklist Item ^16^** | Corresponding pages # |
| --- | --- | --- | --- |
| TITLE | | | |
| Title | 1 | Identify the report as a systematic review *incorporating a network meta-analysis (or related form of meta-analysis).* | #1, Title section |
| ABSTRACT | | | |
| Structured summary | 2 | Provide a structured summary including, as applicable: **Background:** main objectives; **Methods:** data sources; study eligibility criteria, participants, and interventions; study appraisal; and *synthesis methods, such as network meta-analysis.* **Results:** number of studies and participants identified; summary estimates with corresponding confidence/credible intervals; *treatment rankings may also be discussed. Authors may choose to summarize pairwise comparisons against a chosen treatment included in their analyses for brevity.***Discussion/Conclusions:** limitations; conclusions and implications of findings. **Other:** primary source of funding; systematic review registration number with registry name. | #1-2, Abstract section |
| INTRODUCTION | | | |
| Rationale | 3 | Describe the rationale for the review in the context of what is already known*, including mention of why a network meta-analysis has been conducted.* | #3-4, Introduction (2^nd^ paragraph) |
| Objectives | 4 | Provide an explicit statement of questions being addressed, with reference to participants, interventions, comparisons, outcomes, and study design (PICOS). | #3-4, Introduction (3^rd^ paragraph); |
| METHODS | | | |
| Protocol and registration | 5 | Indicate whether a review protocol exists: **PROSPERO register：CRD42018115641** | #5, Method (1^st^ paragraph) |
| Eligibility criteria | 6 | Specify study characteristics (e.g., PICOS, length of follow-up) and report characteristics (e.g., years considered, language, publication status) used as criteria for eligibility, giving rationale. *Clearly describe eligible treatments included in the treatment network, and note whether any have been clustered or merged into the same node (with justification).* | #5-6, Method (2^nd^ paragraph); |

| Information sources | 7 | Describe all information sources (e.g., databases with dates of coverage, contact with study authors to identify additional studies) in the search and date last searched. | #5-6, Method (2^nd^ paragraph); |
| --- | --- | --- | --- |
| Search | 8 | Present full electronic search strategy for at least one database, including any limits used, such that it could be repeated. | #5-6, Method (2^nd^ paragraph); |
| Study selection | 9 | State the process for selecting studies (i.e., screening, eligibility, included in systematic review, and, if applicable, included in the meta-analysis). | #6-7, Method (2^nd^ paragraph); |
| Data collection process | 10 | Describe method of data extraction from reports (e.g., piloted forms, independently, in duplicate) and any processes for obtaining and confirming data from investigators. | #6-7, Method (2^nd^ paragraph) |
| Data items | 11 | List and define all variables for which data were sought (e.g., PICOS, funding sources) and any assumptions and simplifications made. | #6-7, Method (2^nd^ paragraph) |
| **Geometry of the network** | **S1** | Describe methods used to explore the geometry of the treatment network under study and potential biases related to it. This should include how the evidence base has been graphically summarized for presentation, and what characteristics were compiled and used to describe the evidence base to readers. | #7-8, Method (3^rd^ paragraph); |
| Risk of bias within individual studies | 12 | Describe methods used for assessing risk of bias of individual studies (including specification of whether this was done at the study or outcome level), and how this information is to be used in any data synthesis. | #7-8, Method (2^nd^ paragraph |
| Summary measures | 13 | State the principal summary measures (e.g., risk ratio, difference in means). *Also describe the use of additional summary measures assessed, such as treatment rankings and surface under the cumulative ranking curve (SUCRA) values, as well as modified approaches used to present summary findings from meta-analyses.* | #8, Method (2^nd^ paragraph |
| Planned methods of analysis | 14 | Describe the methods of handling data and combining results of studies for each network meta-analysis. | #8, Method (3^rd^ paragraph) |
| **Assessment of Inconsistency** | **S2** | Describe the statistical methods used to evaluate the agreement of direct and indirect evidence in the treatment network(s) studied. Describe efforts taken to address its presence when found. | #8, Method (3^rd^ paragraph) |
| Risk of bias across studies | 15 | Specify any assessment of risk of bias that may affect the cumulative evidence (e.g., publication bias, selective reporting within studies). | #8, Method (3^rd^ paragraph) |
| Additional analyses | 16 | Describe methods of additional analyses if done, indicating which were pre-specified. | #8, Method (3^rd^ paragraph) |
| RESULTS† | | | |
| Study selection | 17 | Give numbers of studies screened, assessed for eligibility, and included in the review, with reasons for exclusions at each stage, ideally with a flow diagram. | #8, Results (1^st^ paragraph) |
| **Presentation of network structure** | **S3** | Provide a network graph of the included studies to enable visualization of the geometry of the treatment network. | Figure 2 |
| **Summary of network geometry** | **S4** | Provide a brief overview of characteristics of the treatment network. This may include commentary on the abundance of trials and randomized patients for the different interventions and pairwise comparisons in the network, gaps of evidence in the treatment network, and potential biases reflected by the network structure. | #8, Results (1^st^ paragraph). |
| Study characteristics | 18 | For each study, present characteristics for which data were extracted (e.g., study size, PICOS, follow-up period) and provide the citations. | #8-12, Results (2^nd^ paragraph);  Table 1. |
| Risk of bias within studies | 19 | Present data on risk of bias of each study and, if available, any outcome level assessment. | #8-12, Results (2^nd^ paragraph); |
| Results of individual studies | 20 | For all outcomes considered (benefits or harms), present, for each study: 1) simple summary data for each intervention group, and 2) effect estimates and confidence intervals. | #8-12, Results (3^rd^ paragraph)  Table. 1 |
| Synthesis of results | 21 | Present results of each meta-analysis done, including confidence/credible intervals. If additional summary measures were explored (such as treatment rankings), these should also be presented. | #8-12, Results (3^rd^ paragraph)  Figure. 3; Figure.4; Figure. 5 |
| **Exploration for inconsistency** | **S5** | Describe results from investigations of inconsistency. This may include such information as measures of model fit to compare consistency and inconsistency models, *P* values from statistical tests, or summary of inconsistency estimates from different parts of the treatment network. | #8-12, Results (4^th^ paragraph);  Figure. 3; Figure.4; Figure. 5 |
| Risk of bias across studies | 22 | Present results of any assessment of risk of bias across studies for the evidence base being studied. | #8-12, Results (4^th^ paragraph);  Table.2 |
| Results of additional analyses | 23 | Give results of additional analyses, if done (e.g., sensitivity or subgroup analyses, meta-regression analyses*, alternative network geometries studied, alternative choice of prior distributions for Bayesian analyses,* and so forth). | #8-12, Results (4^th^ paragraph); |
| **DISCUSSION** |  |  |  |
| Summary of evidence | 24 | Summarize the main findings, including the strength of evidence for each main outcome; consider their relevance to key groups (e.g., healthcare providers, users, and policy-makers). | 13-15 |
| Limitations | 25 | Discuss limitations at study and outcome level (e.g., risk of bias), and at review level (e.g., incomplete retrieval of identified research, reporting bias). *Comment on the validity of the assumptions, such as transitivity and consistency. Comment on any concerns regarding network geometry (e.g., avoidance of certain comparisons).* | 15-16 |
| Conclusions | 26 | Provide a general interpretation of the results in the context of other evidence, and implications for future research. | 17 |
| FUNDING | | | |
| Funding | 27 | Describe sources of funding for the systematic review and other support (e.g., supply of data); role of funders for the systematic review. This should also include information regarding whether funding has been received from manufacturers of treatments in the network and/or whether some of the authors are content experts with professional conflicts of interest that could affect use of treatments in the network. | 17 |

PICOS = population, intervention, comparators, outcomes, study design.

* Text in italics indicateS wording specific to reporting of network meta-analyses that has been added to guidance from the PRISMA statement.

† Authors may wish to plan for use of appendices to present all relevant information in full detail for items in this section.

# Appendix 3: Protocol and Search strategies

Protocol as published in PROSPERO (Registration number: CRD42022337552)

## 3.1. Review eligibility criteria

eTable 3.1. PICOS, Inclusion and exclusion criteria

| **Patient** | Human cadaver or Synthetic bone |
| --- | --- |
| **Intervention** | Metal suture anchor |
| **Comparator** | All-suture anchor (including Inconix, Juggerknot, Q-fix, Y-knot); Biocomposite (Bioabsorbable/ Biomaterials/ Degradable) suture anchor; PEEK (polyetheretherketone) suture anchor |
| **Outcomes** | 1. Load to Failure 2. Stiffness 3. Displacement 4. Failure Mode, including Anchor Pullout, Eyelet Breakage, Suture Breakage |
| **Study design** | Network Meta-analysis |
| **Inclusion criteria** | 1. Rotator cuff repair models using human cadaveric or synthetic specimens 2. Different suture anchor materials were studied 3. Randomized controlled studies or comparative studies 4. Papers published in English 5. Older age of cadaver or osteoporotic bone model. |
| **Exclusion criteria** | 1. Non-biomechanical studies, single arm biomechanical studies, single-arm clinical studies, case series or reports, conference abstracts or comments on other studies 2. Unknown target outcomes of interest 3. Rotator cuff repair models using pediatric, pathologic, and animal specimens 4. Comparison without different suture anchor materials |

## 3.2. Search vocabulary

eTable 3.2. Search Vocabulary

| **Pubmed** | 1. "rotator cuff"[tw] OR ("rotator cuff"[tw] AND (tear*[tw] OR repair*[tw] OR injur*[tw] OR disease*[tw] OR arthropathy*[tw])) 2. "rotator cuff injuries" [mh] OR "rotator cuff" [mh] OR "rotator cuff tear arthropathy" [mh] 3. (("all suture"[tw] OR "all-suture"[tw] OR "full suture"[tw] OR "full-suture"[tw]) AND anchor*[tw]) OR ((soft[tw] OR "all-soft"[tw]) AND (suture*[tw] OR anchor*[tw])) OR "Q-Fix"[tw] OR "SutureFix"[tw] OR "Iconix"[tw] OR "JuggerKnot"[tw] OR "Y-knot"[tw] OR "Draw Tight"[tw] OR "FiberTak"[tw] 4. "suture anchors" [mh] 5. (#1 OR #2) AND (#3 OR #4) |
| --- | --- |
| **EMBASE, PICO search** | 1. ("rotator cuff" OR ("rotator cuff" NEAR/4 (tear* OR repair* OR injur* OR disease* OR arthropathy*))):ti,ab,kw,de 2. "rotator cuff injury"/exp OR "rotator cuff"/exp 3. (("all suture" NEAR/4 anchor*) OR ("all-suture" NEAR/3 anchor*) OR ("full suture" NEAR/4 anchor*) OR ("full-suture" NEAR/3 anchor*) OR (soft NEAR/4 (suture* OR anchor*)) OR ("all-soft" NEAR/3 (suture* OR anchor*)) OR "Q-Fix" OR "SutureFix" OR "Iconix" OR "JuggerKnot" OR "Y-knot" OR "Draw Tight" OR "FiberTak"):ti,ab,kw,de 4. "suture anchor"/exp 5. (#1 OR #2) AND (#3 OR #4) AND [embase]/lim |
| **Cochrane**  **CENTRAL** | 1. ("rotator cuff" OR ("rotator cuff" NEAR/3 (tear* OR repair* OR injur* OR disease* OR arthropathy*))):ti,ab,kw 2. [mh "rotator cuff injuries"] OR [mh "rotator cuff"] OR [mh "rotator cuff tear arthropathy"] 3. (("all suture" NEAR/3 anchor*) OR ("all-suture" NEAR/2 anchor*) OR (soft NEAR/3 (suture* OR anchor*)) OR ("all-soft" NEAR/2 (suture* OR anchor*)) OR ("full suture" NEAR/3 anchor*) OR ("full-suture" NEAR/2 anchor*) OR "Q-Fix" OR "SutureFix" OR "Iconix" OR "JuggerKnot" OR "Y-knot" OR "Draw Tight" OR "FiberTak"):ti,ab,kw 4. [mh "suture anchors"] 5. (#1 OR #2) AND (#3 OR #4) |
| **Scopus** | 1. TITLE-ABS-KEY ("rotator cuff" OR ("rotator cuff" W/3 (tear* OR repair* OR injur* OR disease* OR arthropath*))) 2. TITLE-ABS-KEY (("all suture" W/3 anchor*) OR ("all-suture" W/2 anchor*) OR (soft W/3 (all* OR suture* OR anchor*)) OR ("all-soft" W/2 (suture* OR anchor*)) OR ("full suture" W/3 anchor*) OR ("full-suture" W/2 anchor*) OR "Q-Fix" OR "SutureFix" OR "Iconix" OR "JuggerKnot" OR "Y-knot" OR "Draw Tight" OR "FiberTak") 3. #1 AND #2 |

# Appendix 4: Excluded studies and reasons

1. **Reviews or meta-analysis articles : 7 articles listed in eTable 1**
   1. Day Hazra, R. O., et al. (2021). "The Evolution of Arthroscopic Rotator Cuff Repair." Orthopaedic Journal of Sports Medicine 9(11).
   2. Thangarajah, T., et al. (2021). "Rotator cuff repair techniques: Current concepts." Journal of Clinical Orthopaedics and Trauma 17: 149-156.
   3. Ergün, S., et al. (2020). "The Clinical and Biomechanical Performance of All-Suture Anchors: A Systematic Review." Arthroscopy, Sports Medicine, and Rehabilitation 2(3): e263-e275.
   4. Chaudhry, S., et al. (2019). "A review of suture anchors." Orthopaedics and Trauma 33(4): 263-270.
   5. Parsons, B. and V. Olujimi (2017). "The Evolution of Arthroscopic Rotator Cuff Repair Techniques: What Repair Construct to Use and How to Do It Well." Techniques in Shoulder and Elbow Surgery 18(1): 1-7.
   6. Ma, R., et al. (2011). "Arthroscopic rotator cuff repair: Suture anchor properties, modes of failure and technical considerations." Expert Review of Medical Devices 8(3): 377-387.
   7. Shi, B. Y., et al. (2018). "Biomechanical Strength of Rotator Cuff Repairs: A Systematic Review and Meta-regression Analysis of Cadaveric Studies." Am J Sports Med 47(8): 1984-1993.
2. **Repeated Study: 2 studies**
   1. Müller, S., et al. (2018). "Biomechanical comparison of three anchors for rotator cuff repair in standardized physiological and osteoporotic bone-introducing 1000 loading cycles." Swiss Medical Weekly 148: 4S.
   2. Ntalos D, Huber G, Sellenschloh K, Saito H, Püschel K, Morlock MM, Frosch KH, Klatte TO. All-suture anchor pullout results in decreased bone damage and depends on cortical thickness. Knee Surg Sports Traumatol Arthrosc. 2021 Jul;29(7):2212-2219. doi: 10.1007/s00167-020-06004-6. Epub 2020 Apr 24. PMID: 32333058; PMCID: PMC8225531.
3. **Specimens: NOT Human Cadaver or Synthetic Bone Model: 3 studies**
   1. Godry H, Jettkant B, Seybold D, Venjakob AJ, Bockmann B. Pullout strength and failure mode of industrially manufactured and self-made all-suture anchors: a biomechanical analysis. J Shoulder Elbow Surg. 2020 Jul; 29(7):1479-1483. doi: 10.1016/j.jse.2019.12.007. Epub 2020 Mar 17. PMID: 32197807.
   2. Sano H, Tokunaga M, Noguchi M, Inawashiro T, Irie T, Abe H, Abrassart S, Itoi E. Comparison of fixation properties between coil-type and screw-type anchors for rotator cuff repair: A virtual pullout testing using 3-dimensional finite element method. J Orthop Sci. 2016 Jul;21(4):452-457. doi: 10.1016/j.jos.2016.04.011. Epub 2016 May 14. PMID: 27188930.
   3. Barber FA, Herbert MA. Cyclic loading biomechanical analysis of the pullout strengths of rotator cuff and glenoid anchors: 2013 update. Arthroscopy. 2013 May;29(5):832-44. doi: 10.1016/j.arthro.2013.01.028. Epub 2013 Apr 2. PMID: 23561482.
4. **Study without comparisons of different suture anchor materials: 7 studies**
   1. Trofa DP, Ruder JA, Yeatts NC, Peindl RD, Habet NA, Saltzman BM, Fleischli JE. Cyclic and Load-to-Failure Properties of All-Suture Anchors in Human Cadaveric Shoulder Greater Tuberosities. Arthroscopy. 2020 Nov;36(11):2805-2811. doi: 10.1016/j.arthro.2020.06.010. Epub 2020 Jun 15. PMID: 32554073.
   2. Aliaj K, Henninger HB, Tétreault-Paquin JE, Getelman MH, Donahue JP. Biomechanics of an interlinked suture anchor rotator cuff repair in a human cadaveric model. JSES Open Access. 2019 Apr 26;3(2):70-76. doi: 10.1016/j.jses.2019.02.002. PMID: 31334432; PMCID: PMC6620261.
   3. Nagamoto, H., et al. (2016). "Effect of friction of suture anchors on the “deadman angle theory”: A biomechanical study." Journal of Orthopaedic Research 34.
   4. Barber FA, Herbert MA, Hapa O, Rapley JH, Barber CA, Bynum JA, Hrnack SA. Biomechanical analysis of pullout strengths of rotator cuff and glenoid anchors: 2011 update. Arthroscopy. 2011 Jul;27(7):895-905. doi: 10.1016/j.arthro.2011.02.016. PMID: 21693345.
   5. Barber FA, Coons DA, Ruiz-Suarez M. Cyclic load testing of biodegradable suture anchors containing 2 high-strength sutures. Arthroscopy. 2007 Apr;23(4):355-60. doi: 10.1016/j.arthro.2006.12.009. PMID: 17418326.
   6. Aramberri-Gutiérrez M, Martínez-Menduiña A, Boyle S, Valencia M. Biomechanical testing of trans-humeral all-suture anchors for rotator cuff repair. Shoulder Elbow. 2019 May;11(1 Suppl):77-85. doi: 10.1177/1758573218779078. Epub 2018 Aug 4. PMID: 31019566; PMCID: PMC6463375.
   7. Ammon JT, Nyland J, Chang HC, Burden R, Caborn DN. Evaluation of BioCorkscrew and Bioknotless RC suture anchor rotator cuff repair fixation: an in vitro biomechanical study. Knee Surg Sports Traumatol Arthrosc. 2007 Nov;15(11):1375-81. doi: 10.1007/s00167-007-0321-0. Epub 2007 Apr 17. PMID: 17437085.
5. **Study about Suture Bridge Techniques in Double Row Repairs: 5 studies**
   1. Bernardoni ED, Frank RM, Veera SS, Waterman BR, Griffin JW, Shewman EF, Cole BJ, Romeo AA, Verma NN. Biomechanical Analysis of Medial-Row All-Suture Suture Anchor Fixation for Rotator Cuff Repair in a Pair-Matched Cadaveric Model. Arthroscopy. 2019 May;35(5):1370-1376. doi: 10.1016/j.arthro.2019.01.023. Epub 2019 Apr 15. PMID: 31000387.
   2. Bernardoni E, Frank RM, Veera SS, Griffin JW, Waterman BR, Shewman E, Cole BJ, Romeo AA, Verma NN. Biomechanical Analysis of All-Suture Anchor Fixation for Rotator Cuff Repair. Orthop J Sports Med. 2018 Jul 27;6(7 suppl4):2325967118S00175. doi: 10.1177/2325967118S00175. PMID: 30128313; PMCID: PMC6093985.
   3. Galland A, Airaudi S, Gravier R, Le Cann S, Chabrand P, Argenson JN. Pullout strength of all suture anchors in the repair of rotator cuff tears: a biomechanical study. Int Orthop. 2013 Oct;37(10):2017-23. doi: 10.1007/s00264-013-1984-4. Epub 2013 Jul 9. PMID: 23835556; PMCID: PMC3779582.
   4. Goschka AM, Hafer JS, Reynolds KA, Aberle NS 2nd, Baldini TH, Hawkins MJ, McCarty EC. Biomechanical comparison of traditional anchors to all-suture anchors in a double-row rotator cuff repair cadaver model. Clin Biomech (Bristol, Avon). 2015 Oct;30(8):808-13. doi: 10.1016/j.clinbiomech.2015.06.009. Epub 2015 Jun 14. PMID: 26117162.
   5. Müller, P. E., et al. (2013). "Biomechanical aspects of rotator cuff repair: Influence of suture materials, knot techniques and suture techniques on the primary stability." Sport-Orthopadie - Sport-Traumatologie 29(1): 45-48.

# Appendix 5: List of included studies

## 5.1. Load to Failure

### 5.1.1. Study group and primary outcome

eTable 5.1.1. Randomized controlled trials or comparative studies in Load to Failure

| **Author, Year** | **Country** | **No. of specimens** | **No. of anchors** | **Analyzed Interventions** | **Bone Mineral Density** |
| --- | --- | --- | --- | --- | --- |
| Yamauchi et al, 2022 | Japan | 160 | 160 | Corkscrew FT Ti 4.5mm, HEALICOIL PK 4.5mm, Corkscrew Bio 4.75mm | (1) 10-pounds/cubic foot  (2) 5-pounds/cubic foot |
| Rosso et al, 2020 | Switzerland | 60 | 60 | TwinFix Ti 4.5mm, Healix BR 4.5mm, Iconix 2.3mm | (1) Physiological group, 0.12 g/cm3;  (2) Osteoporotic group, 0.09 g/cm3 |
| Ntalos et al, 2019 | Germany | 10 | 15 | Y-knot 2.8mm, CrossFT 4.5mm | (1) 126 ± 25 mg/cm3 in ASA group  (2) 127 ± 30 mg/cm3 in conventional anchor |
| Ntalos et al, 2019 | Germany | 36 | 28 | Y-knot 2.8mm, TwinFix ultra PK 6.5mm | (1) 126 ± 18 mg /cm3  (2) 126 ± 26 mg /cm3  (3) 127 ±16 mg /cm3 |
| Nagra et al, 2017 | UK | 24 | 24 | Bio-Corkscrew FT 5.5mm,  CrossFT PK 5.5mm | N/A |
| Barber et al, 2010 | USA | 16 | 96 | Hemodynamic parameters | N/A |
| Pietschmann et al, 2009 | Germany | 12 | 36 | SPIRALOK BC 5.0 mm , Super Revo 5.0 mm | (1) Non-osteopenic bones: 0.109 ± 0.026 g/cm3  (2) Osteoporotic bones: 0.041 ± 0.020 g/cm3 |

### 5.1.2. Study population and enrollment criteria

eTable 5.1.2. Definition and inclusion criteria in trials of Load to Failure

| **Author, Year** | **Specimens** | **Bone mineral density definition** | **All Interventions** | **Testing procedures** |
| --- | --- | --- | --- | --- |
| Yamauchi et al, 2022 | 160 Sawbone models | A 10- pound per cubic foot (pcf) model (part #1522-01) was used as a normal cancellous bone model, and a 5-pcf model (part #1522-23) was used as an osteoporotic cancellous bone model. | TwinFix Ti 5.0 mm and 6.5 mm and Corkscrew FT 4.5 mm, 5.5 mm, and 6.5 mm. HEALICOIL PK (HC-PK) 4.5 mm and 5.5 mm and SwiveLock PK 4.75 mm and 5.5 mm. HC-RG 4.75 mm and 5.5 mm, Corkscrew Bio 4.75 mm, 5.5 mm, and 6.5 mm, and SwiveLock BC 4.75 mm and 5.5 mm. | Pullout testing was performed in parallel to the insertion axis at a displacement rate of 12.5 mm/s. |
| Rosso et al, 2020 | 60 Sawbone models | Artificial bone specimens with standardized bone densities (physiological, 0.12 g/cm3, 2 mm cortical layer; osteoporotic, 0.09 g/cm3, 1 mm cortical layer) | Three currently used anchors were used in this study, the titanium TwinFix Ti 4.5-mm, the bioresorbable Healix BR 4.5-mm, and the Iconix 2.3-mm all-suture anchor. | Preconditioned the bone-anchor constructs with a pre- load of 100 N, cyclic loading was continuously performed in a sinusoidal load-controlled route between 10 and 100 N at a frequency of 0.5 Hz for 1000 loading cycles. After 1000 loading cycles, a preload of 55 N was applied before strain-controlled ultimate load to failure (ULTF) testing was performed for all constructs at a strain rate of 3.15 mm/s until failure of the samples occurred. |
| Ntalos et al, 2019 | 10 Human humerus | A total of 10 human humeral bones (5 matched pairs) were collected from donors aged between 50 and 73 years. Determination of volumetric bone mineral density revealed similar values in both groups (126 ± 25 mg/cm3 in ASA/ revision group and 127 ± 30 mg/cm3 in 4.5- mm conventional anchor group). | Three different anchor types were tested: a commercially available ASA with a drill size of 2.8 mm and a diameter greater than 5 mm after dilatation (Y-Knot RC), a conventional 4.5-mm suture anchor (CrossFT), and a conventional 5.5-mm suture anchor (CrossFT). | Starting with a preload of 20 N, a cyclic testing protocol at a rate of 1 Hz with a stepwise increase to the maximum pullout force was performed at 90 degrees regarding the anchor and bone surface. The respective upper peak load started at 50 N and increased by 0.05 N during each cycle till suture anchor failure. |
| Ntalos et al, 2019 | 36 Human humerus | 36 human humeri (18 matched pairs) were collected from donors, between the age of 22–76 years (mean 61.4 years, standard deviation 11 years). The usage of matched pairs caused comparable vBMD for both treatments (all-suture anchor vs. conventional treatment) in all three groups. | Two different anchor types were tested on each matched pair. The Y-Knot RC an all-suture anchor with a drill size of 2.8mm and the conventional 4.50mm CrossFT Suture Anchor. | After an initial preload with 20N a cyclic testing protocol with a stepwise increasing pullout of 0.05N for each cycle at a rate of 1Hz was performed. Cyclic extension was continued until system failure. Displacement was defined as difference between the initial construct length after pre-load and the subsequent clamp to anchor distance at 1000, 2000 and 3000 cycles. |
| Nagra et al, 2017 | 24 Human humerus | 12 matched pairs, age range 58 to 96, 4:2 ratio of men = women. | All-suture anchors tested included the y-Knot, Q-FIX, ICoNIX and JuggerKnot. a commonly used traditional plastic anchor (TWINFIX ultra PK Suture anchors) was used as a comparator and control. | Tensile testing to failure was then performed using a Zwick/Roell tensile testing machine and a 10 kN load cell, with a clamp-to-clamp distance of 100 mm. An initial 10 N tension was placed on the anchors to ensure proper deployment of the subcortical segment of the all-suture anchors and to prevent loading artefacts. |
| Barber et al, 2010 | 16 Human humerus | The mean age was 80 years (range, 70 to 96 years), and there was only 1 female donor. | The 4 medial anchors tested were the Bio-Corkscrew FT, the CrossFT PEEK, the TwinFix PK FT, and the Healix PEEK. | The constructs were pre- loaded with 10 N at 1 N/s. The preload was held for 5 seconds, and then the constructs were cycled from 10 to 60 N at 1 Hz for 500 cycles. For those constructs surviving the 500 cycles, this was followed by a single pull to failure at 33 mm/s, consistent with prior reports. |
| Pietschmann et al, 2009 | 12 Human humerus | Twelve human humeri from 12 cadavers, eight male and four female bones, with a mean age of 57 years at the time of death (range 27–93 years) were used. Six non-osteoporotic bones (BMD: 0.109 ± 0.026 g/cm3) with an average age of 42.4 years and six osteoporotic bones (BMD: 0.041 ± 0.020 g/cm3) with an average age of 73.8 years were used. | Two screw anchors and one tilting anchor were tested: SPIRALOK 5.0 mm, Super Revo 5 mm and UltraSorb anchor. | A preload of 20 N and a crosshead extension rate of 20 mm/min were selected [18]. Fifty cycles with a tensile load of 75 N were applied, and then the tensile load was increased to 100 N for another 50 cycles. Until failure of the anchor fixation system, the tensile load was gradually increased by 25 N per 50 cycles. The ultimate failure loads, the total system displacement, as well as the system displacement after the first pull with 75 N were recorded during testing. |

### 5.1.3. Baseline characteristics

eTable 5.1.3. Baseline characteristics in trials with Load to Failure

| **Author, Year** | **Arm** | **Mean in Osteoporotic BMD** | **SD in Osteoporotic BMD** | **Mean in Physiological BMD** | **SD in Physiological BMD** |
| --- | --- | --- | --- | --- | --- |
| Yamauchi et al, 2022 | TwinFix Ti 5.0mm | 64.9 | 1.1 | 175.2 | 11.4 |
|  | TwinFix Ti 6.5 mm | 102.1 | 16.2 | 304 | 15.2 |
|  | Corkscrew FT 4.5mm | 59.1 | 3.7 | 160.1 | 11 |
|  | Corkscrew FT 5.5mm | 89.5 | 3.6 | 248.6 | 10 |
|  | Corkscrew FT 6.5mm | 99.4 | 2.7 | 247.3 | 9.3 |
|  | HEALICOIL PK 4.5mm | 115.8 | 5.5 | 226.5 | 10.7 |
|  | HEALICOIL PK 5.5mm | 146.3 | 5.8 | 235.6 | 27.7 |
|  | SwiveLock PK 4.75mm | 68.8 | 3.6 | 140 | 37.3 |
|  | SwiveLock PK 5.5mm | 78.8 | 7.41 | 180.8 | 4.8 |
|  | HEALICOIL RG 4.75mm | 119 | 1.4 | 237.9 | 3.5 |
|  | HEALICOIL RG 5.5mm | 134.2 | 3.4 | 266.1 | 7.9 |
|  | Corkscrew Bio 4.75mm | 29.3 | 2 | 212.2 | 15.6 |
|  | Corkscrew Bio 5.5mm | 107.9 | 4.6 | 234.8 | 11.8 |
|  | Corkscrew Bio 6.5mm | 102.1 | 7.8 | 230.2 | 38.5 |
| Rosso et al, 2020 | TwinFix Ti 4.5mm | 63.2 | 6.8 | 140 | 6.3 |
|  | Healix BR 4.5mm | 86.7 | 3.8 | 190.5 | 7.9 |
|  | Iconix 2.3mm | 467.4 | 39.4 | 497.1 | 50.5 |
| Ntalos et al, 2019 | Y-knot 2.8mm | 315.1 | 11.3 | N/A | N/A |
|  | CrossFT 4.5mm | 500.9 | 50.6 | N/A | N/A |
|  | CrossFT 5.5mm | 271 | 106 | N/A | N/A |
| Ntalos et al, 2019 | Y-knot 2.8mm | 295 | 43 | N/A | N/A |
|  | CrossFT 4.5mm | 281 | 87 | N/A | N/A |
| Nagra et al, 2017 | Y-knot 2.8mm | 247 | 78 | N/A | N/A |
|  | Q-fix 2.8mm | 259 | 61 | N/A | N/A |
|  | Iconix 2.3mm | 145.8 | 51.65 | N/A | N/A |
|  | JuggerKnot 2.9mm | 144.9 | 29.96 | N/A | N/A |
|  | TwinFix ultra PK | 103.9 | 22.58 | N/A | N/A |
| Barber et al, 2010 | Bio-Corkscrew FT 5.5 | 137.8 | 45.39 | N/A | N/A |
|  | CrossFT PK 5.5 | 181 | 35.2 | N/A | N/A |
|  | TwinFix PK FT 5.5 | 209.3 | 96.65 | N/A | N/A |
|  | Healix PK 5.5 | 269.3 | 121.24 | N/A | N/A |
|  | PopLok PEEK 4.5 | 263.7 | 107.04 | N/A | N/A |
|  | PEEK PushLock 5.5 | 295.9 | 131.98 | N/A | N/A |
|  | Footprint PEEK anchor 5.5 | 209.8 | 69.97 | N/A | N/A |
|  | Versalok PEEK 4.9 | 201.7 | 147.92 | N/A | N/A |
| Pietschmann et al, 2009 | SPIRALOK BC 5.0 mm | 163.2 | 108.77 | 274 | 29 |
|  | Super Revo 5 mm | 308 | 64.09 | 188 | 34 |
|  | UltraSorb anchor | 171 | 19 | 192 | 34 |

## 5.2. Stiffness

### 5.2.1. Study group and primary outcome

eTable 5.2.1. Randomized controlled trials or comparative studies in Stiffness

| **Author, Year** | **Country** | **No. of specimens** | **No. of anchors** | **Analyzed Interventions** | **Bone Mineral Density** |
| --- | --- | --- | --- | --- | --- |
| Rosso et al, 2020 | Switzerland | 60 | 60 | TwinFix Ti 4.5mm, Healix BR 4.5mm, Iconix 2.3mm | (1) Physiological group, 0.12 g/cm3;  (2) Osteoporotic group, 0.09 g/cm3 |
| Ntalos et al, 2019 | Germany | 10 | 15 | Y-knot 2.8mm, CrossFT 4.5mm | (1) 126 ± 25 mg/cm3 in ASA group  (2) 127 ± 30 mg/cm3 in conventional anchor |
| Ntalos et al, 2019 | Germany | 36 | 28 | Y-knot 2.8mm, TwinFix ultra PK 6.5mm | (1) 126 ± 18 mg /cm3  (2) 126 ± 26 mg /cm3  (3) 127 ±16 mg /cm3 |

### 5.2.2. Study population and enrollment criteria

eTable 5.2.2. Definition and inclusion criteria in trials of Stiffness

| **Author, Year** | **Specimens** | **Bone mineral density definition** | **All Interventions** | **Testing procedures** |
| --- | --- | --- | --- | --- |
| Rosso et al, 2020 | 60 Sawbone models | Artificial bone specimens with standardized bone densities (physiological, 0.12 g/cm3, 2 mm cortical layer; osteoporotic, 0.09 g/cm3, 1 mm cortical layer) | Three currently used anchors were used in this study, the titanium TwinFix Ti 4.5-mm, the bioresorbable Healix BR 4.5-mm, and the Iconix 2.3-mm all-suture anchor. | Preconditioned the bone-anchor constructs with a pre- load of 100 N, cyclic loading was continuously performed in a sinusoidal load-controlled route between 10 and 100 N at a frequency of 0.5 Hz for 1000 loading cycles. After 1000 loading cycles, a preload of 55 N was applied before strain-controlled ultimate load to failure (ULTF) testing was performed for all constructs at a strain rate of 3.15 mm/s until failure of the samples occurred. |
| Ntalos et al, 2019 | 10 Human humerus | A total of 10 human humeral bones (5 matched pairs) were collected from donors aged between 50 and 73 years. Determination of volumetric bone mineral density revealed similar values in both groups (126 ± 25 mg/cm3 in ASA/ revision group and 127 ± 30 mg/cm3 in 4.5- mm conventional anchor group). | Three different anchor types were tested: a commercially available ASA with a drill size of 2.8 mm and a diameter greater than 5 mm after dilatation (Y-Knot RC), a conventional 4.5-mm suture anchor (CrossFT), and a conventional 5.5-mm suture anchor (CrossFT). | Starting with a preload of 20 N, a cyclic testing protocol at a rate of 1 Hz with a stepwise increase to the maximum pullout force was performed at 90 degrees regarding the anchor and bone surface. The respective upper peak load started at 50 N and increased by 0.05 N during each cycle till suture anchor failure. |
| Ntalos et al, 2019 | 36 Human humerus | 36 human humeri (18 matched pairs) were collected from donors, between the age of 22–76 years (mean 61.4 years, standard deviation 11 years). The usage of matched pairs caused comparable vBMD for both treatments (all-suture anchor vs. conventional treatment) in all three groups. | Two different anchor types were tested on each matched pair. The Y-Knot RC an all-suture anchor with a drill size of 2.8mm and the conventional 4.50mm CrossFT Suture Anchor. | After an initial preload with 20N a cyclic testing protocol with a stepwise increasing pullout of 0.05N for each cycle at a rate of 1Hz was performed. Cyclic extension was continued until system failure. Displacement was defined as difference between the initial construct length after pre-load and the subsequent clamp to anchor distance at 1000, 2000 and 3000 cycles. |

### 5.2.3. Baseline characteristics

eTable 5.2.3. Baseline characteristics in trials with Stiffness

| **Author, Year** | **Arm** | **Mean in Osteoporotic BMD** | **SD in Osteoporotic BMD** | **Mean in Physiological BMD** | **SD in Physiological BMD** |
| --- | --- | --- | --- | --- | --- |
| **Rosso et al, 2020** | TwinFix Ti 4.5mm | 0.37 | 0.2 | 0.44 (P) | 0.16 |
|  | Healix BR 4.5mm | 0.29 | 0.03 | 0.33 | 0.02 |
|  | Iconix 2.3mm | 0.39 | 0.21 | 0.59 | 0.09 |
| **Ntalos et al, 2019** | Y-knot 2.8mm | 74 | 9.4 | N/A | N/A |
|  | CrossFT 4.5mm | 74 | 2 | N/A | N/A |
|  | CrossFT 5.5mm | 68 | 5.7 | N/A | N/A |
| **Ntalos et al, 2019** | Y-knot 2.8mm | 66.1 | 10.2 | N/A | N/A |
|  | CrossFT 4.5mm | 61.9 | 6.82 | N/A | N/A |

## 5.3. Displacement

### 5.3.1. Study group and primary outcome

eTable 5.3.1. Randomized controlled trials or comparative studies in Displacement

| **Author, Year** | **Country** | **No. of specimens** | **No. of anchors** | **Analyzed Interventions** | **Bone Mineral Density** |
| --- | --- | --- | --- | --- | --- |
| Rosso et al, 2020 | Switzerland | 60 | 60 | TwinFix Ti 4.5mm, Healix BR 4.5mm, Iconix 2.3mm | (1) Physiological group, 0.12 g/cm3;  (2) Osteoporotic group, 0.09 g/cm3 |
| Ntalos et al, 2019 | Germany | 10 | 15 | Y-knot 2.8mm, CrossFT 4.5mm | (1) 126 ± 25 mg/cm3 in ASA group  (2) 127 ± 30 mg/cm3 in conventional anchor |
| Ntalos et al, 2019 | Germany | 36 | 28 | Y-knot 2.8mm, TwinFix ultra PK 6.5mm | (1) 126 ± 18 mg /cm3  (2) 126 ± 26 mg /cm3  (3) 127 ±16 mg /cm3 |
| Nagra et al, 2017 | UK | 24 | 24 | Bio-Corkscrew FT 5.5mm,  CrossFT PK 5.5mm | N/A |
| Pietschmann et al, 2009 | Germany | 12 | 36 | SPIRALOK BC 5.0 mm , Super Revo 5.0 mm | (1) Non-osteopenic bones: 0.109 ± 0.026 g/cm3  (2) Osteoporotic bones: 0.041 ± 0.020 g/cm3 |

### 5.3.2. Study population and enrollment criteria

eTable 5.3.2. Definition and inclusion criteria in trials of Displacement

| **Author, Year** | **Specimens** | **Bone mineral density definition** | **All Interventions** | **Testing procedures** |
| --- | --- | --- | --- | --- |
| Rosso et al, 2020 | 60 Sawbone models | Artificial bone specimens with standardized bone densities (physiological, 0.12 g/cm3, 2 mm cortical layer; osteoporotic, 0.09 g/cm3, 1 mm cortical layer) | Three currently used anchors were used in this study, the titanium TwinFix Ti 4.5-mm, the bioresorbable Healix BR 4.5-mm, and the Iconix 2.3-mm all-suture anchor. | Preconditioned the bone-anchor constructs with a pre- load of 100 N, cyclic loading was continuously performed in a sinusoidal load-controlled route between 10 and 100 N at a frequency of 0.5 Hz for 1000 loading cycles. After 1000 loading cycles, a preload of 55 N was applied before strain-controlled ultimate load to failure (ULTF) testing was performed for all constructs at a strain rate of 3.15 mm/s until failure of the samples occurred. |
| Ntalos et al, 2019 | 10 Human humerus | A total of 10 human humeral bones (5 matched pairs) were collected from donors aged between 50 and 73 years. Determination of volumetric bone mineral density revealed similar values in both groups (126 ± 25 mg/cm3 in ASA/ revision group and 127 ± 30 mg/cm3 in 4.5- mm conventional anchor group). | Three different anchor types were tested: a commercially available ASA with a drill size of 2.8 mm and a diameter greater than 5 mm after dilatation (Y-Knot RC), a conventional 4.5-mm suture anchor (CrossFT), and a conventional 5.5-mm suture anchor (CrossFT). | Starting with a preload of 20 N, a cyclic testing protocol at a rate of 1 Hz with a stepwise increase to the maximum pullout force was performed at 90 degrees regarding the anchor and bone surface. The respective upper peak load started at 50 N and increased by 0.05 N during each cycle till suture anchor failure. |
| Ntalos et al, 2019 | 36 Human humerus | 36 human humeri (18 matched pairs) were collected from donors, between the age of 22–76 years (mean 61.4 years, standard deviation 11 years). The usage of matched pairs caused comparable vBMD for both treatments (all-suture anchor vs. conventional treatment) in all three groups. | Two different anchor types were tested on each matched pair. The Y-Knot RC an all-suture anchor with a drill size of 2.8mm and the conventional 4.50mm CrossFT Suture Anchor. | After an initial preload with 20N a cyclic testing protocol with a stepwise increasing pullout of 0.05N for each cycle at a rate of 1Hz was performed. Cyclic extension was continued until system failure. Displacement was defined as difference between the initial construct length after pre-load and the subsequent clamp to anchor distance at 1000, 2000 and 3000 cycles. |
| Nagra et al, 2017 | 24 Human humerus | 12 matched pairs, age range 58 to 96, 4:2 ratio of men = women. | All-suture anchors tested included the y-Knot, Q-FIX, ICoNIX and JuggerKnot. a commonly used traditional plastic anchor (TWINFIX ultra PK Suture anchors) was used as a comparator and control. | Tensile testing to failure was then performed using a Zwick/Roell tensile testing machine and a 10 kN load cell, with a clamp-to-clamp distance of 100 mm. An initial 10 N tension was placed on the anchors to ensure proper deployment of the subcortical segment of the all-suture anchors and to prevent loading artefacts. |
| Pietschmann et al, 2009 | 12 Human humerus | Twelve human humeri from 12 cadavers, eight male and four female bones, with a mean age of 57 years at the time of death (range 27–93 years) were used. Six non-osteoporotic bones (BMD: 0.109 ± 0.026 g/cm3) with an average age of 42.4 years and six osteoporotic bones (BMD: 0.041 ± 0.020 g/cm3) with an average age of 73.8 years were used. | Two screw anchors and one tilting anchor were tested: SPIRALOK 5.0 mm, Super Revo 5 mm and UltraSorb anchor. | A preload of 20 N and a crosshead extension rate of 20 mm/min were selected [18]. Fifty cycles with a tensile load of 75 N were applied, and then the tensile load was increased to 100 N for another 50 cycles. Until failure of the anchor fixation system, the tensile load was gradually increased by 25 N per 50 cycles. The ultimate failure loads, the total system displacement, as well as the system displacement after the first pull with 75 N were recorded during testing. |

### 5.3.3. Baseline characteristics

eTable 5.3.3. Baseline characteristics in trials with Displacement

| **Author, Year** | **Arm** | **Mean in Osteoporotic BMD** | **SD in Osteoporotic BMD** | **Mean in Physiological BMD** | **SD in Physiological BMD** |
| --- | --- | --- | --- | --- | --- |
| **Rosso et al, 2020** | TwinFix Ti 4.5mm | 14.9 | 0.9 | 15 | 0.9 |
|  | Healix BR 4.5mm | 16.6 | 1 | 16.7 | 1 |
|  | Iconix 2.3mm | 16.5 | 1.6 | 16.5 | 1.6 |
| **Ntalos et al, 2019** | Y-knot 2.8mm | 21.9 | 1.22 | N/A | N/A |
|  | CrossFT 4.5mm | 23.5 | 1.46 | N/A | N/A |
|  | CrossFT 5.5mm | 21.36 | 2.32 | N/A | N/A |
| **Ntalos et al, 2019** | Y-knot 2.8mm | 10.67 | 2.47 | N/A | N/A |
|  | CrossFT 4.5mm | 11.6 | 2.71 | N/A | N/A |
| **Nagra et al, 2017** | Y-knot 2.8mm | 15.1 | 4.7 | N/A | N/A |
|  | Q-fix 2.8mm | 11.8 | 7.6 | N/A | N/A |
|  | Iconix 2.3mm | 17.9 | 4.25 | N/A | N/A |
|  | JuggerKnot 2.9mm | 22.6 | 5.59 | N/A | N/A |
|  | TwinFix ultra PK | 12.5 | 0.6 | N/A | N/A |
| **Pietschmann et al, 2009** | SPIRALOK BC 5.0 mm | 3.47 | 2.87 | 1.81 | 1.08 |
|  | Super Revo 5 mm | 2.06 | 1.63 | 1.53 | 1.39 |
|  | UltraSorb anchor | 2.71 | 1.14 | 2.58 | 1.56 |

## 5.4. Failure mode

### 5.4.1. Study group and primary outcome

eTable 5.4.1. Randomized controlled trials or comparative studies in Failure mode

| **Author, Year** | **Country** | **No. of specimens** | **No. of anchors** | **Analyzed Interventions** | **Bone Mineral Density** |
| --- | --- | --- | --- | --- | --- |
| Yamauchi et al, 2022 | Japan | 160 | 160 | Corkscrew FT Ti 4.5mm, HEALICOIL PK 4.5mm, Corkscrew Bio 4.75mm | (1) 10-pounds/cubic foot  (2) 5-pounds/cubic foot |
| Ntalos et al, 2019 | Germany | 10 | 15 | Y-knot 2.8mm, CrossFT 4.5mm | (1) 126 ± 25 mg/cm3 in ASA group  (2) 127 ± 30 mg/cm3 in conventional anchor |
| Ntalos et al, 2019 | Germany | 36 | 28 | Y-knot 2.8mm, TwinFix ultra PK 6.5mm | (1) 126 ± 18 mg /cm3  (2) 126 ± 26 mg /cm3  (3) 127 ±16 mg /cm3 |
| Nagra et al, 2017 | UK | 24 | 24 | Bio-Corkscrew FT 5.5mm,  CrossFT PK 5.5mm | N/A |
| Barber et al, 2010 | USA | 16 | 96 | Hemodynamic parameters | N/A |
| Pietschmann et al, 2009 | Germany | 12 | 36 | SPIRALOK BC 5.0 mm , Super Revo 5.0 mm | (1) Non-osteopenic bones: 0.109 ± 0.026 g/cm3  (2) Osteoporotic bones: 0.041 ± 0.020 g/cm3 |

### 5.4.2. Study population and enrollment criteria

eTable 5.4.2. Definition and inclusion criteria in trials of Failure mode

| **Author, Year** | **Specimens** | **Bone mineral density definition** | **All Interventions** | **Testing procedures** |
| --- | --- | --- | --- | --- |
| Yamauchi et al, 2022 | 160 Sawbone models | A 10- pound per cubic foot (pcf) model (part #1522-01) was used as a normal cancellous bone model, and a 5-pcf model (part #1522-23) was used as an osteoporotic cancellous bone model. | TwinFix Ti 5.0 mm and 6.5 mm and Corkscrew FT 4.5 mm, 5.5 mm, and 6.5 mm. HEALICOIL PK (HC-PK) 4.5 mm and 5.5 mm and SwiveLock PK 4.75 mm and 5.5 mm. HC-RG 4.75 mm and 5.5 mm, Corkscrew Bio 4.75 mm, 5.5 mm, and 6.5 mm, and SwiveLock BC 4.75 mm and 5.5 mm. | Pullout testing was performed in parallel to the insertion axis at a displacement rate of 12.5 mm/s. |
| Ntalos et al, 2019 | 10 Human humerus | A total of 10 human humeral bones (5 matched pairs) were collected from donors aged between 50 and 73 years. Determination of volumetric bone mineral density revealed similar values in both groups (126 ± 25 mg/cm3 in ASA/ revision group and 127 ± 30 mg/cm3 in 4.5- mm conventional anchor group). | Three different anchor types were tested: a commercially available ASA with a drill size of 2.8 mm and a diameter greater than 5 mm after dilatation (Y-Knot RC), a conventional 4.5-mm suture anchor (CrossFT), and a conventional 5.5-mm suture anchor (CrossFT). | Starting with a preload of 20 N, a cyclic testing protocol at a rate of 1 Hz with a stepwise increase to the maximum pullout force was performed at 90 degrees regarding the anchor and bone surface. The respective upper peak load started at 50 N and increased by 0.05 N during each cycle till suture anchor failure. |
| Ntalos et al, 2019 | 36 Human humerus | 36 human humeri (18 matched pairs) were collected from donors, between the age of 22–76 years (mean 61.4 years, standard deviation 11 years). The usage of matched pairs caused comparable vBMD for both treatments (all-suture anchor vs. conventional treatment) in all three groups. | Two different anchor types were tested on each matched pair. The Y-Knot RC an all-suture anchor with a drill size of 2.8mm and the conventional 4.50mm CrossFT Suture Anchor. | After an initial preload with 20N a cyclic testing protocol with a stepwise increasing pullout of 0.05N for each cycle at a rate of 1Hz was performed. Cyclic extension was continued until system failure. Displacement was defined as difference between the initial construct length after pre-load and the subsequent clamp to anchor distance at 1000, 2000 and 3000 cycles. |
| Nagra et al, 2017 | 24 Human humerus | 12 matched pairs, age range 58 to 96, 4:2 ratio of men = women. | All-suture anchors tested included the y-Knot, Q-FIX, ICoNIX and JuggerKnot. a commonly used traditional plastic anchor (TWINFIX ultra PK Suture anchors) was used as a comparator and control. | Tensile testing to failure was then performed using a Zwick/Roell tensile testing machine and a 10 kN load cell, with a clamp-to-clamp distance of 100 mm. An initial 10 N tension was placed on the anchors to ensure proper deployment of the subcortical segment of the all-suture anchors and to prevent loading artefacts. |
| Barber et al, 2010 | 16 Human humerus | The mean age was 80 years (range, 70 to 96 years), and there was only 1 female donor. | The 4 medial anchors tested were the Bio-Corkscrew FT, the CrossFT PEEK, the TwinFix PK FT, and the Healix PEEK. | The constructs were pre- loaded with 10 N at 1 N/s. The preload was held for 5 seconds, and then the constructs were cycled from 10 to 60 N at 1 Hz for 500 cycles. For those constructs surviving the 500 cycles, this was followed by a single pull to failure at 33 mm/s, consistent with prior reports. |
| Pietschmann et al, 2009 | 12 Human humerus | Twelve human humeri from 12 cadavers, eight male and four female bones, with a mean age of 57 years at the time of death (range 27–93 years) were used. Six non-osteoporotic bones (BMD: 0.109 ± 0.026 g/cm3) with an average age of 42.4 years and six osteoporotic bones (BMD: 0.041 ± 0.020 g/cm3) with an average age of 73.8 years were used. | Two screw anchors and one tilting anchor were tested: SPIRALOK 5.0 mm, Super Revo 5 mm and UltraSorb anchor. | A preload of 20 N and a crosshead extension rate of 20 mm/min were selected [18]. Fifty cycles with a tensile load of 75 N were applied, and then the tensile load was increased to 100 N for another 50 cycles. Until failure of the anchor fixation system, the tensile load was gradually increased by 25 N per 50 cycles. The ultimate failure loads, the total system displacement, as well as the system displacement after the first pull with 75 N were recorded during testing. |

### 5.4.3. Baseline characteristics

eTable 5.4.3. Baseline characteristics in trials with Failure mode

| **Author, Year** | **Arm** | **Anchor pullout in Osteoporotic BMD** | **Eyelet breakage in Osteoporotic BMD** | **Suture breakage in Osteoporotic BMD** |
| --- | --- | --- | --- | --- |
| Yamauchi et al, 2022 | TwinFix Ti 5.0mm | 5 | 0 | 0 |
|  | TwinFix Ti 6.5 mm | 5 | 0 | 0 |
|  | Corkscrew FT 4.5mm | 5 | 0 | 0 |
|  | Corkscrew FT 5.5mm | 5 | 0 | 0 |
|  | Corkscrew FT 6.5mm | 5 | 0 | 0 |
|  | HEALICOIL PK 4.5mm | 5 | 0 | 0 |
|  | HEALICOIL PK 5.5mm | 5 | 0 | 0 |
|  | SwiveLock PK 4.75mm | 5 | 0 | 0 |
|  | SwiveLock PK 5.5mm | 5 | 0 | 0 |
|  | HEALICOIL RG 4.75mm | 5 | 0 | 0 |
|  | HEALICOIL RG 5.5mm | 5 | 0 | 0 |
|  | Corkscrew Bio 4.75mm | 0 | 5 | 0 |
|  | Corkscrew Bio 5.5mm | 5 | 0 | 0 |
|  | Corkscrew Bio 6.5mm | 5 | 0 | 0 |
| Ntalos et al, 2019 | Y-knot 2.8mm | 5 | 0 | 0 |
|  | CrossFT 4.5mm | 5 | 0 | 0 |
|  | CrossFT 5.5mm | 5 | 0 | 0 |
| Ntalos et al, 2019 | Y-knot 2.8mm | 14 | 0 | 0 |
|  | CrossFT 4.5mm | 13 | 0 | 1 |
| Nagra et al, 2017 | Y-knot 2.8mm | 5 | 0 | 0 |
|  | Q-fix 2.8mm | 5 | 0 | 0 |
|  | Iconix 2.3mm | 5 | 0 | 0 |
|  | JuggerKnot 2.9mm | 4 | 0 | 1 |
|  | TwinFix ultra PK | 1 | 1 | 2 |
| Barber et al, 2010 | Bio-Corkscrew FT 5.5 | 10 | 1 | 0 |
|  | CrossFT PK 5.5 | 12 | 0 | 0 |
|  | TwinFix PK FT 5.5 | 9 | 2 | 1 |
|  | Healix PK 5.5 | 10 | 1 | 0 |
|  | PopLok PEEK 4.5 | 11 | 0 | 0 |
|  | PEEK PushLock 5.5 | 7 | 3 | 0 |
|  | Footprint PEEK anchor 5.5 | 8 | 3 | 0 |
|  | Versalok PEEK 4.9 | 10 | 0 | 2 |
| Pietschmann et al, 2009 | SPIRALOK BC 5.0 mm | 5 | 1 | 0 |
|  | Super Revo 5 mm | 3 | 3 | 0 |
|  | UltraSorb anchor | 5 | 0 | 1 |

eTable 5.4.4. Included Arm, Mean and Standard Deviation of Each Outcomes

| **Author, Year** | **Arm** | **Load to Failure** | | **Stiffness** | | **Displacement** | | **Failure mode** | | |
| --- | --- | --- | --- | --- | --- | --- | --- | --- | --- | --- |
|  |  | **Mean** | **SD** | **Mean** | **SD** | **Mean** | **SD** | **Anchor pullout** | **Eyelet breakage** | **Suture breakage** |
| Yamauchi et al, 2022 | Corkscrew FT 4.5mm | 59.1 | 3.7 | N/A | N/A | N/A | N/A | 5 | 0 | 0 |
|  | HEALICOIL PK 4.5mm | 115.8 | 5.5 | N/A | N/A | N/A | N/A | 5 | 0 | 0 |
|  | Corkscrew Bio 4.75mm | 29.3 | 2 | N/A | N/A | N/A | N/A | 0 | 5 | 0 |
| Rosso et al, 2020 | TwinFix Ti 4.5mm | 467.4 | 39.4 | 0.37 | 0.2 | 14.9 | 0.9 | 5 | 0 | 0 |
|  | Healix BR 4.5mm | 315.1 | 11.3 | 0.29 | 0.03 | 16.6 | 1 | 5 | 0 | 0 |
|  | Iconix 2.3mm | 500.9 | 50.6 | 0.39 | 0.21 | 16.5 | 1.6 | 14 | 0 | 0 |
| Ntalos et al, 2019 | Y-knot 2.8mm | 271 | 106 | 74 | 9.4 | 21.9 | 1.22 | 13 | 0 | 1 |
|  | CrossFT 4.5mm | 295 | 43 | 74 | 2 | 23.5 | 1.46 | 5 | 0 | 0 |
| Ntalos et al, 2019 | Y-knot 2.8mm | 247 | 78 | 66.1 | 10.2 | 10.67 | 2.47 | 1 | 1 | 2 |
|  | CrossFT 4.5mm | 259 | 61 | 61.9 | 6.82 | 11.6 | 2.71 | 10 | 1 | 0 |
| Nagra et al, 2017 | Y-knot 2.8mm | 145.8 | 51.65 | N/A | N/A | 15.1 | 4.7 | 12 | 0 | 0 |
|  | TwinFix ultra PK | 181 | 35.2 | N/A | N/A | 12.5 | 0.6 | 5 | 1 | 0 |
| Barber et al, 2010 | Bio-Corkscrew FT 5.5 | 209.3 | 96.65 | N/A | N/A | N/A | N/A | 3 | 3 | 0 |
|  | CrossFT PK 5.5 | 269.3 | 121.24 | N/A | N/A | N/A | N/A | 14 | 0 | 0 |
| Pietschmann et al, 2009 | SPIRALOK BC 5.0 mm | 171 | 19 | N/A | N/A | 3.47 | 2.87 | 14 | 0 | 0 |
|  | Super Revo 5 mm | 150 | 42 | N/A | N/A | 2.06 | 1.63 | 5 | 0 | 0 |

## 5.5. References

Referenced to studies included in this review

1. **Yamauchi et al, 2022**

Yamauchi S, Tsukada H, Sasaki E, et al. Biomechanical analysis of bioabsorbable suture anchors for rotator cuff repair using osteoporotic and normal bone models. Article. Journal of Orthopaedic Science. 2022;27(1):115-121. doi:10.1016/j.jos.2020.11.017

1. **Rosso et al, 2020**

Rosso C, Weber T, Dietschy A, de Wild M, Müller S. Three anchor concepts for rotator cuff repair in standardized physiological and osteoporotic bone: a biomechanical study. Article. Journal of Shoulder and Elbow Surgery. 2020;29(2):e52-e59. doi:10.1016/j.jse.2019.07.032

1. **Ntalos et al, 2019**

Ntalos D, Huber G, Sellenschloh K, et al. Biomechanical analysis of conventional anchor revision after all-suture anchor pullout: a human cadaveric shoulder model. Article. Journal of Shoulder and Elbow Surgery. 2019;28(12):2433-2437. doi:10.1016/j.jse.2019.04.053

1. **Ntalos et al, 2019**

Ntalos D, Sellenschloh K, Huber G, et al. Conventional rotator cuff versus all-suture anchors—A biomechanical study focusing on the insertion angle in an unlimited cyclic model. Article. PLoS ONE. 2019;14(11)doi:10.1371/journal.pone.0225648

1. **Nagra et al, 2017**

Nagra NS, Zargar N, Smith RDJ, Carr AJ. Mechanical properties of all-suture anchors for rotator cuff repair. Article. Bone and Joint Research. 2017;6(2):82-89. doi:10.1302/2046-3758.62.BJR-2016-0225.R1

1. **Barber et al, 2010**

Barber FA, Hapa O, Bynum JA. Comparative testing by cyclic loading of rotator cuff suture anchors containing multiple high-strength sutures. Article. Arthroscopy - Journal of Arthroscopic and Related Surgery. 2010;26(9 SUPPL. 1):S134-S141+e166. doi:10.1016/j.arthro.2010.03.007

1. **Pietschmann et al, 2009**

Pietschmann MF, Fröhlich V, Ficklscherer A, et al. Suture anchor fixation strength in osteopenic versus non-osteopenic bone for rotator cuff repair. Article. Archives of Orthopaedic and Trauma Surgery. 2009;129(3):373-379. doi:10.1007/s00402-008-0689-4

# Appendix 6: Assessment of transitivity

Before statistical analysis, we assessed the transitivity assumption by checking the included trials in the NMA were on average similar in terms of characteristics that might modify the treatment effect. Those characteristics in sepsis trials included

1. Anchor Numbers
2. Human cadaver: Synthetic bone rate
3. Anchor Insertion Angle
4. Bone mineral density
5. Age of Human Cadaver
6. Publication Year

## 6.1. Anchor Numbers

eFigure 6.1: Assessment of transitivity in Anchor Numbers

## 6.2. Human Cadaver: Synthetic bone rate

eFigure 6.2: Assessment of transitivity in Human cadaver: Synthetic bone rate

| % | All-suture | Biocomposite | PEEK | Metal |
| --- | --- | --- | --- | --- |
| Human | 70.59 | 54.55 | 87.5 | 28.57 |
| Synthetic | 29.41 | 45.45 | 12.5 | 71.43 |

## 6.3. Anchor Insertion Angle

eFigure 6.3: Assessment of transitivity in Anchor Insertion Angle

| % | All-suture | Biocomposite | PEEK | Metal |
| --- | --- | --- | --- | --- |
| 45 degrees | 50 | 76.2 | 20 | 76.2 |
| 90 degrees | 33.33 | 23.8 | 60 | 23.8 |
| 100 degrees | 16.67 | 0 | 20 | 0 |

## 6.4. Bone Mineral Density

eTable 6.4.1: Assessment of transitivity in Bone Mineral Density

|  | All-suture | Biocomposite | PEEK | Metal |
| --- | --- | --- | --- | --- |
| Mean | 114.17 | 73.62 | 117.2 | 73.62 |
| SD | 24.26 | 21.01 | 27.89 | 21.01 |

eTable 6.4.2: Assessment of transitivity in Bone Mineral Density after excluding artificial bones.

|  | All-suture | Biocomposite | PEEK | Metal |
| --- | --- | --- | --- | --- |
| Mean | 126.25 | 41.0 | 126.5 | 41.0 |
| SD | 21.09 | 20.0 | 23.24 | 20.0 |

## 6.5. Age of Human cadaver

eTable 6.5: Assessment of transitivity in Age of Human cadaver

|  | All-suture | Biocomposite | PEEK | Metal |
| --- | --- | --- | --- | --- |
| Mean | 64.67 | 79.9 | 70.78 | 73.8 |
| SD | 6.324 | 4.34 | 10.07 | N/A |

## 6.6. Publication Year

eTable 6.6: Assessment of transitivity in Publication Year

|  | All-suture | Biocomposite | PEEK | Metal |
| --- | --- | --- | --- | --- |
| Mean | 2019 | 2015.4 | 2016.5 | 2017.3 |
| SD | 0.939 | 4.966 | 4.41 | 5.33 |

# Appendix 7: Risk of bias

## 7.1. Load to Failure

### 7.1.1. Risk of bias assessment for the individual domains: Load to Failure

eFigure 7.1.1.: Risk of bias assessment for the individual domains in Load to Failure

### 7.1.2. Risk of bias assessment for the individual studies: Load to Failure

| Author, Year | Domain 1 | Domain 2 | Domain 3 | Domain 4 | Domain 5 | Overall Bias |  |  |
| --- | --- | --- | --- | --- | --- | --- | --- | --- |
| Yamauchi et al, 2022 |  |  |  |  |  |  |  | Domain :   1. Randomization process 2. Deviations from intended interventions 3. Missing outcome data 4. Measurement of the outcome 5. Selection of the reported result |
| Raosso et al, 2020 |  |  |  |  |  |  |  |  |
| Ntalos et al, 2019 |  |  |  |  |  |  |  |  |
| Ntalos et al, 2019 |  |  |  |  |  |  |  |  |
| Nagra et al, 2017 |  |  |  |  |  |  |  |  |
| Barber et al, 2010 |  |  |  |  |  |  |  |  |
| Pietschman et al, 2009 |  |  |  |  |  |  |  |  |

eFigure 7.1.2.: Risk of bias assessment for the individual studies in Load to Failure

### 7.1.3. Risk of bias notes for the individual studies: Load to Failure

eTable 7.1.1. Risk of bias notes for the individual studies in Load to Failure

| Study ID | Notes for risk of bias assessment |
| --- | --- |
| Yamauchi et al, 2022 | Domain 2. There were 16 types of suture anchors with several materials, diameters and shapes, which were much different from anchors included in other studies. |
|  | Domain 5. We choose three suture anchors with different materials, which were far from the numbers of anchors included in the study. |
| Ntalos et al, 2019 | Domain 1. The distributions of anchors to specimens were not illustrated in details. |
|  | Domain 5. PEEK anchors with larger diameter were implanted after all-suture anchors pullout, which were not included in our analysis. |
| Nagra et al, 2017 | Domain 5. There were four types of all-suture anchors made in different companies, but we choose the one which was included in other studies. |
| Barber et al, 2010 | Domain 2. There were 7 types of PEEK anchors with several diameters and shapes, which were much different from anchors included in other studies. |
|  | Domain 5. We choose one type of PEEK anchors with larger diameter, which was included in other studies. |
| Pietschman et al, 2009 | Domain 2. Suture anchors that included in the study were not utilized in clinical nowadays, which were much different from anchors included in other studies. |

## 7.2. Stiffness

### 7.2.1. Risk of bias assessment for the individual domains: Stiffness

eFigure 7.2:1.: Risk of bias assessment for the individual domains in Stiffness

### 7.2.2. Risk of bias assessment for the individual studies: Stiffness

| Author, Year | Domain 1 | Domain 2 | Domain 3 | Domain 4 | Domain 5 | Overall Bias |  |  |
| --- | --- | --- | --- | --- | --- | --- | --- | --- |
| Raosso et al, 2020 |  |  |  |  |  |  |  | Domain :   1. Randomization process 2. Deviations from intended interventions 3. Missing outcome data 4. Measurement of the outcome 5. Selection of the reported result |
| Ntalos et al, 2019 |  |  |  |  |  |  |  |  |
| Ntalos et al, 2019 |  |  |  |  |  |  |  |  |
|  |  |  |  |  |  |  |  |  |
|  |  |  |  |  |  |  |  |  |

eFigure 7.2.2.: Risk of bias assessment for the individual studies in Stiffness

### 7.2.3. Risk of bias notes for the individual studies: Stiffness

eTable 7.2. Risk of bias notes for the individual studies in Stiffness

| Study ID | Notes for risk of bias assessment |
| --- | --- |
| Ntalos et al, 2019 | Domain 1. The distributions of anchors to specimens were not illustrated in details. |
|  | Domain 5. PEEK anchors with larger diameter were implanted after all-suture anchors pullout, which were not included in our analysis. |

## 7.3. Displacement

### 7.3.1. Risk of bias assessment for the individual domains: Displacement

eFigure 7.3.1.: Risk of bias assessment for the individual domains in Displacement

### 7.3.2. Risk of bias assessment for the individual studies: Displacement

| Author, Year | Domain 1 | Domain 2 | Domain 3 | Domain 4 | Domain 5 | Overall Bias |  |  |
| --- | --- | --- | --- | --- | --- | --- | --- | --- |
| Raosso et al, 2020 |  |  |  |  |  |  |  | Domain :   1. Randomization process 2. Deviations from intended interventions 3. Missing outcome data 4. Measurement of the outcome 5. Selection of the reported result |
| Ntalos et al, 2019 |  |  |  |  |  |  |  |  |
| Ntalos et al, 2019 |  |  |  |  |  |  |  |  |
| Nagra et al, 2017 |  |  |  |  |  |  |  |  |
| Pietschman et al, 2009 |  |  |  |  |  |  |  |  |

eFigure 7.3.2.: Risk of bias assessment for the individual studies in Displacement

### 7.3.3. Risk of bias notes for the individual studies: Displacement

eTable 7.3. Risk of bias notes for the individual studies in Displacement

| Study ID | Notes for risk of bias assessment |
| --- | --- |
| Ntalos et al, 2019 | Domain 1. The distributions of anchors to specimens were not illustrated in details. |
|  | Domain 5. PEEK anchors with larger diameter were implanted after all-suture anchors pullout, which were not included in our analysis. |
| Nagra et al, 2017 | Domain 5. There were four types of all-suture anchors made in different companies, but we choose the one which was included in other studies. |
| Pietschman et al, 2009 | Domain 2. Suture anchors that included in the study were not utilized in clinical nowadays, which were much different from anchors included in other studies. |

## 7.4. Failure modes

### 7.4.1. Risk of bias assessment for the individual domains in Failure mode

eFigure 7.4.1.: Risk of bias assessment for the individual domains in failure mode

### 7.4.2. Risk of bias assessment for the individual studies: Failure modes

| Author, Year | Domain 1 | Domain 2 | Domain 3 | Domain 4 | Domain 5 | Overall Bias |  |  |
| --- | --- | --- | --- | --- | --- | --- | --- | --- |
| Yamauchi et al, 2022 |  |  |  |  |  |  |  | Domain :   1. Randomization process 2. Deviations from intended interventions 3. Missing outcome data 4. Measurement of the outcome 5. Selection of the reported result |
| Ntalos et al, 2019 |  |  |  |  |  |  |  |  |
| Ntalos et al, 2019 |  |  |  |  |  |  |  |  |
| Nagra et al, 2017 |  |  |  |  |  |  |  |  |
| Barber et al, 2010 |  |  |  |  |  |  |  |  |
| Pietschman et al, 2009 |  |  |  |  |  |  |  |  |

eFigure 7.4.2.: Risk of bias assessment for the individual studies in Failure modes

### 7.4.3. Risk of bias notes for the individual studies: Failure modes

eTable 7.4. Risk of bias notes for the individual studies in Failure mode

| Study ID | Notes for risk of bias assessment |
| --- | --- |
| Yamauchi et al, 2022 | Domain 2. There were 16 types of suture anchors with several materials, diameters and shapes, which were much different from anchors included in other studies. |
|  | Domain 5. We choose three suture anchors with different materials, which were far from the numbers of anchors included in the study. |
| Ntalos et al, 2019 | Domain 1. The distributions of anchors to specimens were not illustrated in details. |
|  | Domain 5. PEEK anchors with larger diameter were implanted after all-suture anchors pullout, which were not included in our analysis. |
| Ntalos et al, 2019 |  |
|  |  |
| Nagra et al, 2017 | Domain 5. There were four types of all-suture anchors made in different companies, but we choose the one which was included in other studies. |
| Barber et al, 2010 | Domain 2. There were 7 types of PEEK anchors with several diameters and shapes, which were much different from anchors included in other studies. |
|  | Domain 5. We choose one type of PEEK anchors with larger diameter, which was included in other studies. |
| Pietschman et al, 2009 | Domain 2. Suture anchors that included in the study were not utilized in clinical nowadays, which were much different from anchors included in other studies. |

# Appendix 8: Results

## 8.1. Extracted outcome data in Load to failure

### 8.1.1. List of Load to failure in each study

eTable 8.1.1 List of Load to Failure in each study

| Author, Year | Treatment | Mean | Standard deviation | No. of cases |
| --- | --- | --- | --- | --- |
| Yamauchi et al, 2022 | Metal | 59.1 | 3.7 | 5 |
|  | PEEK | 115.8 | 5.5 | 5 |
|  | Biocomposite | 29.3 | 2 | 5 |
| Rosso et al, 2020 | Metal | 467.4 | 39.4 | 10 |
|  | Biocomposite | 315.1 | 11.3 | 10 |
|  | All-suture | 500.9 | 50.6 | 10 |
| Ntalos et al, 2019 | All-suture | 271 | 106 | 5 |
|  | PEEK | 295 | 43 | 5 |
| Ntalos et al, 2019 | All-suture | 247 | 78 | 14 |
|  | PEEK | 259 | 61 | 14 |
| Nagra et al, 2017 | All-suture | 145.8 | 51.65 | 5 |
|  | PEEK | 181 | 35.2 | 4 |
| Barber et al, 2010 | Biocomposite | 209.3 | 96.65 | 12 |
|  | PEEK | 269.3 | 121.24 | 12 |
| Pietschman et al, 2009 | Biocomposite | 171 | 19 | 6 |
|  | Metal | 150 | 42 | 6 |

### 8.1.2. Values of load to failure in each suture anchor

eTable 8.1.2 Values of Load to Failure in each suture anchor

|  | All-suture anchor | | | Biocomposite | | | PEEK | | | Metal | | |
| --- | --- | --- | --- | --- | --- | --- | --- | --- | --- | --- | --- | --- |
| Author, Year | m | sd | n | m | sd | n | m | sd | n | m | sd | n |
| Yamauchi et al, 2022 | N/A | N/A | N/A | 29.3 | 2 | 5 | 115.8 | 5.5 | 5 | 59.1 | 3.7 | 5 |
| Rosso et al, 2020 | 500.9 | 50.6 | 10 | 315.1 | 11.3 | 10 | N/A | N/A | N/A | 467.4 | 39.4 | 10 |
| Ntalos et al, 2019 | 271 | 106 | 5 | N/A | N/A | N/A | 295 | 43 | 5 | N/A | N/A | N/A |
| Ntalos et al, 2019 | 247 | 78 | 14 | N/A | N/A | N/A | 259 | 61 | 14 | N/A | N/A | N/A |
| Nagra et al, 2017 | 145.8 | 51.65 | 5 | N/A | N/A | N/A | 181 | 35.2 | 4 | N/A | N/A | N/A |
| Barber et al, 2010 | N/A | N/A | N/A | 209.3 | 96.65 | 12 | 269.3 | 121.2 | 12 | N/A | N/A | N/A |
| Pietschman et al. 2009 | N/A | N/A | N/A | 171 | 19 | 6 | N/A | N/A | N/A | 150 | 42 | 6 |

m, mean of volume; sd: standard deviation of volume; n, sample size

## 8.2. Extracted outcome data in Stiffness

### 8.2.1. List of stiffness in each study

eTable 8.2.1 List of Stiffness in each study

| Author, Year | Treatment | Mean | Standard deviation | No. of cases |
| --- | --- | --- | --- | --- |
| Rosso et al, 2020 | Metal | 0.37 | 0.2 | 10 |
|  | Biocomposite | 0.29 | 0.03 | 10 |
|  | All-suture | 0.39 | 0.21 | 10 |
| Ntalos et al, 2019 | All-suture | 74 | 9.4 | 5 |
|  | PEEK | 74 | 2 | 5 |
| Ntalos et al, 2019 | All-suture | 66.1 | 10.2 | 14 |
|  | PEEK | 61.9 | 6.82 | 14 |

### 8.2.2. Values of stiffness in each suture anchor

eTable 8.2.2 Values of Stiffness in each suture anchor

|  | All-suture anchor | | | Biocomposite | | | PEEK | | | Metal | | |
| --- | --- | --- | --- | --- | --- | --- | --- | --- | --- | --- | --- | --- |
| Author, Year | m | sd | n | m | sd | n | m | sd | n | m | sd | n |
| Yamauchi et al, 2022 | N/A | N/A | N/A | N/A | N/A | N/A | N/A | N/A | N/A | N/A | N/A | N/A |
| Rosso et al, 2020 | 0.39 | 0.21 | 10 | 0.29 | 0.03 | 10 | N/A | N/A | N/A | 0.37 | 0.2 | 10 |
| Ntalos et al, 2019 | 74 | 9.4 | 5 | N/A | N/A | N/A | 74 | 2 | 5 | N/A | N/A | N/A |
| Ntalos et al, 2019 | 66.1 | 10.2 | 14 | N/A | N/A | N/A | 61.9 | 6.82 | 14 | N/A | N/A | N/A |
| Nagra et al, 2017 | N/A | N/A | N/A | N/A | N/A | N/A | N/A | N/A | N/A | N/A | N/A | N/A |
| Barber et al, 2010 | N/A | N/A | N/A | N/A | N/A | N/A | N/A | N/A | N/A | N/A | N/A | N/A |
| Pietschman et al. 2009 | N/A | N/A | N/A | N/A | N/A | N/A | N/A | N/A | N/A | N/A | N/A | N/A |

m, mean of volume; sd: standard deviation of volume; n, sample size

## 8.3. Extracted outcome data in Displacement

### 8.3.1. List of displacement in each study

eTable 8.3.1 List of Displacement in each study

| Author, Year | Treatment | Mean | Standard deviation | No. of cases |
| --- | --- | --- | --- | --- |
| Rosso et al, 2020 | Metal | 14.9 | 0.9 | 10 |
|  | Biocomposite | 16.6 | 1 | 10 |
|  | All-suture | 16.5 | 1.6 | 10 |
| Ntalos et al, 2019 | All-suture | 21.9 | 1.22 | 5 |
|  | PEEK | 23.5 | 1.46 | 5 |
| Ntalos et al, 2019 | All-suture | 10.67 | 2.47 | 14 |
|  | PEEK | 11.6 | 2.71 | 14 |
| Nagra et al, 2017 | All-suture | 15.1 | 4.7 | 5 |
|  | PEEK | 12.5 | 0.6 | 4 |
| Pietschman et al, 2009 | Biocomposite | 3.47 | 2.87 | 6 |
|  | Metal | 2.06 | 1.63 | 6 |

### 8.3.2. Values of displacement in each suture anchor

eTable 8.3.2 Values of Displacement in each suture anchor

|  | All-suture anchor | | | Biocomposite | | | PEEK | | | Metal | | |
| --- | --- | --- | --- | --- | --- | --- | --- | --- | --- | --- | --- | --- |
| Author, Year | m | sd | n | m | sd | n | m | sd | n | m | sd | n |
| Yamauchi et al, 2022 | N/A | N/A | N/A | N/A | N/A | N/A | N/A | N/A | N/A | N/A | N/A | N/A |
| Rosso et al, 2020 | 16.5 | 1.6 | 10 | 16.6 | 1 | 10 | N/A | N/A | N/A | 14.9 | 0.9 | 10 |
| Ntalos et al, 2019 | 21.9 | 1.22 | 5 | N/A | N/A | N/A | 23.5 | 1.46 | 5 | N/A | N/A | N/A |
| Ntalos et al, 2019 | 10.67 | 2.47 | 14 | N/A | N/A | N/A | 11.6 | 2.71 | 14 | N/A | N/A | N/A |
| Nagra et al, 2017 | 15.1 | 4.7 | 5 | N/A | N/A | N/A | 12.5 | 0.6 | 4 | N/A | N/A | N/A |
| Barber et al, 2010 | N/A | N/A | N/A | N/A | N/A | N/A | N/A | N/A | N/A | N/A | N/A | N/A |
| Pietschman et al. 2009 | N/A | N/A | N/A | 3.47 | 2.87 | 6 | N/A | N/A | N/A | 2.06 | 1.63 | 6 |

m, mean of volume; sd: standard deviation of volume; n, sample size

## 8.4. Extracted outcome data in Failure modes

### 8.4.1. Events of anchor pullout in each suture anchor

eTable 8.4.1 Events of Anchor Pullout in each suture anchor

| Author, Year | All-suture anchor | | Biocomposite | | PEEK | | Metal | |
| --- | --- | --- | --- | --- | --- | --- | --- | --- |
|  | d | n | d | n | d | n | d | n |
| Yamauchi et al, 2022 | N/A | N/A | 0 | 5 | 5 | 5 | 5 | 5 |
| Rosso et al, 2020 | N/A | N/A | N/A | N/A | N/A | N/A | N/A | N/A |
| Ntalos et al, 2019 | 5 | 5 | N/A | N/A | 5 | 5 | N/A | N/A |
| Ntalos et al, 2019 | 14 | 14 | N/A | N/A | 13 | 14 | N/A | N/A |
| Nagra et al, 2017 | 5 | 5 | N/A | N/A | 1 | 4 | N/A | N/A |
| Barber et al, 2010 | N/A | N/A | 10 | 12 | 12 | 12 | N/A | N/A |
| Pietschman et al, 2009 | N/A | N/A | 5 | 6 | N/A | N/A | 3 | 6 |

d: no. of case with events, n: no. of case.

### 8.4.2. Events of eyelet breakage in each suture anchor

eTable 8.4.2 Events of Eyelet Breakage in each suture anchor

| Author, Year | All-suture anchor | | Biocomposite | | PEEK | | Metal | |
| --- | --- | --- | --- | --- | --- | --- | --- | --- |
|  | d | n | d | n | d | n | d | n |
| Yamauchi et al, 2022 | N/A | N/A | 5 | 5 | 0 | 5 | 0 | 5 |
| Rosso et al, 2020 | N/A | N/A | N/A | N/A | N/A | N/A | N/A | N/A |
| Ntalos et al, 2019 | 0 | 5 | N/A | N/A | 0 | 5 | N/A | N/A |
| Ntalos et al, 2019 | 0 | 14 | N/A | N/A | 0 | 14 | N/A | N/A |
| Nagra et al, 2017 | 0 | 5 | N/A | N/A | 1 | 4 | N/A | N/A |
| Barber et al, 2010 | N/A | N/A | 1 | 12 | 0 | 12 | N/A | N/A |
| Pietschman et al, 2009 | N/A | N/A | 1 | 6 | N/A | N/A | 3 | 6 |

d: no. of case with events, n: no. of case.

### 8.4.3. Events of suture breakage in each suture anchor

eTable 8.4.3 Events of Suture Breakage in each suture anchor

| Author, Year | All-suture anchor | | Biocomposite | | PEEK | | Metal | |
| --- | --- | --- | --- | --- | --- | --- | --- | --- |
|  | d | n | d | n | d | n | d | n |
| Yamauchi et al, 2022 | N/A | N/A | 0 | 5 | 0 | 5 | 0 | 5 |
| Rosso et al, 2020 | N/A | N/A | N/A | N/A | N/A | N/A | N/A | N/A |
| Ntalos et al, 2019 | 0 | 5 | N/A | N/A | 0 | 5 | N/A | N/A |
| Ntalos et al, 2019 | 0 | 14 | N/A | N/A | 1 | 14 | N/A | N/A |
| Nagra et al, 2017 | 0 | 5 | N/A | N/A | 2 | 4 | N/A | N/A |
| Barber et al, 2010 | N/A | N/A | 0 | 12 | 0 | 12 | N/A | N/A |
| Pietschman et al, 2009 | N/A | N/A | 0 | 6 | N/A | N/A | 0 | 6 |

d: no. of case with events, n: no. of case

# Appendix 9: League table, Pairwise and Relative ranking

## 9.1. Load to Failure

### 9.1.1. Load to Failure in league table and pairwise

eTable 9.1.1 Load to Failure in league table and pairwise

| **Metal** | 33.500  (-6.248, 73.248) | -54.259  (-139.113, 30.596) | 56.700  (50.890, 62.510) |
| --- | --- | --- | --- |
| -51.60  (-138.34, 34.97) | **All-suture** | -185.800  (-217.934, -153.666) | 22.714  (-13.095, 58.523) |
| 49.75  (-17.24, 116.74) | 101.44 (19.13, 183.74) | **Biocomposite** | 86.410  (81.289, 91.531) |
| -56.25  (-137.46, 24.97) | -4.56  (-71.91, 62.78) | -106.00  (-180.33, -31.66) | **PEEK** |

Abbreviations: PEEK, polyetheretherketone

*Results of the network meta-analysis are presented in the left lower half. Odd ratio and 95% confidence interval were presented and left hand side intervention was reference group. Odds ratio less than one favor the column-defining treatment.

*Results of the pairwise meta-analysis are presented in the right upper half. Odd ratio and 95% confidence interval were presented and right hand side intervention was reference group. Odds ratio less than one favor the column-defining treatment.

eTable 9.1.2 Significant test of ES: Load to Failure


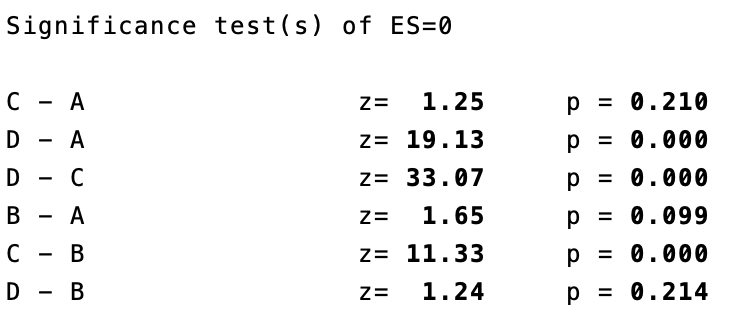


*A: Metal anchor; B: All-suture anchor; C: Biocomposite anchor; D: PEEK anchor.

### 9.1.2. Load to Failure in relative ranking probability

eTable 9.1.3 Load to Failure in relative ranking probability

| 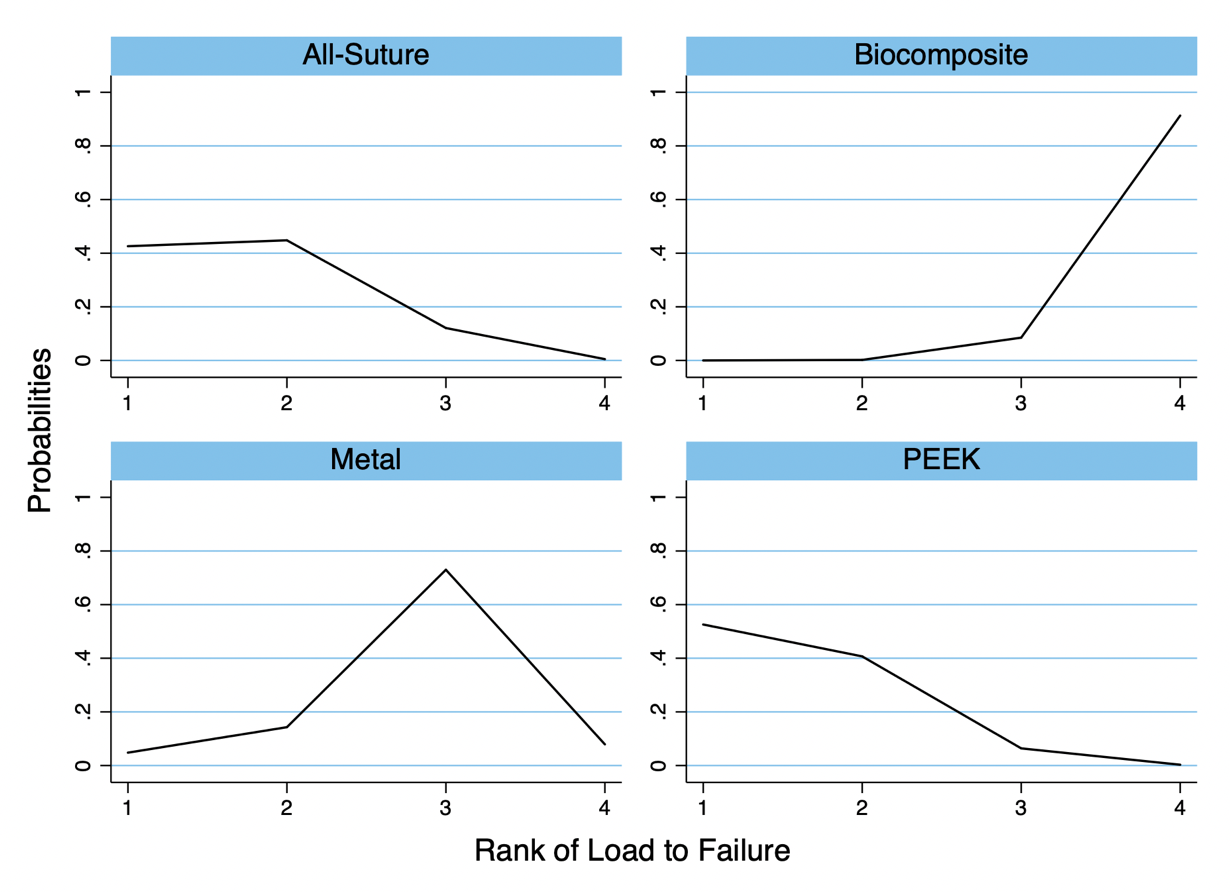 | | | | |
| --- | --- | --- | --- | --- |
| Ranking\Treatment | Metal | All-suture | Biocomposite | PEEK |
| Best | 4.8 | 42.6 | 0.0 | 52.6 |
| Mean Rank | 2.8 | 1.7 | 3.9 | 1.5 |
| SUCRA | 38.7 | 76.5 | 3.0 | 81.9 |

Abbreviations: PEEK, polyetheretherketone; SUCRA, the surface under the cumulative ranking.

## 9.2. Stiffness

### 9.2.1. Stiffness in league table and pairwise

eTable 9.2.1 Stiffness in league table and pairwise

| **Metal** | 0.020  (-0.160, 0.200) | -0.080  (-0.205, 0.045) | - |
| --- | --- | --- | --- |
| -0.02  (-0.20, 0.16) | **All-suture** | -0.100  (-0.231, 0.031) | -2.655  (-7.764, 2.455) |
| 0.08  (-0.05, 0.21) | 0.10 (-0.03, 0.23) | **Biocomposite** | - |
| -2.63  (-2.48, 7.75) | 2.65  (-2.46, 7.76) | 2.55  (-2.56, 7.66) | **PEEK** |

Abbreviations: PEEK, polyetheretherketone

*Results of the network meta-analysis are presented in the left lower half. Odd ratio and 95% confidence interval were presented and left hand side intervention was reference group. Odds ratio less than one favor the column-defining treatment.

*Results of the pairwise meta-analysis are presented in the right upper half. Odd ratio and 95% confidence interval were presented and right hand side intervention was reference group. Odds ratio less than one favor the column-defining treatment.

eTable 9.2.2 Significant test of ES: Stiffness


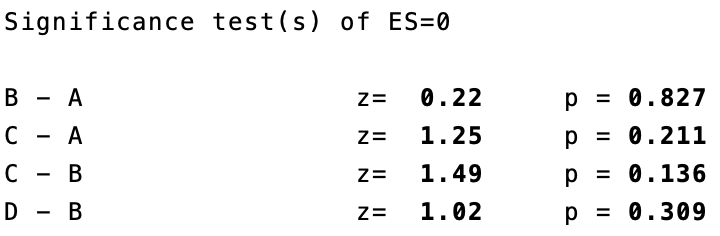


*A: Metal anchor; B: All-suture anchor; C: Biocomposite anchor; D: PEEK anchor.

### 9.2.2. Stiffness in relative ranking probability

eTable 9.2.3 Stiffness in relative ranking probability

| 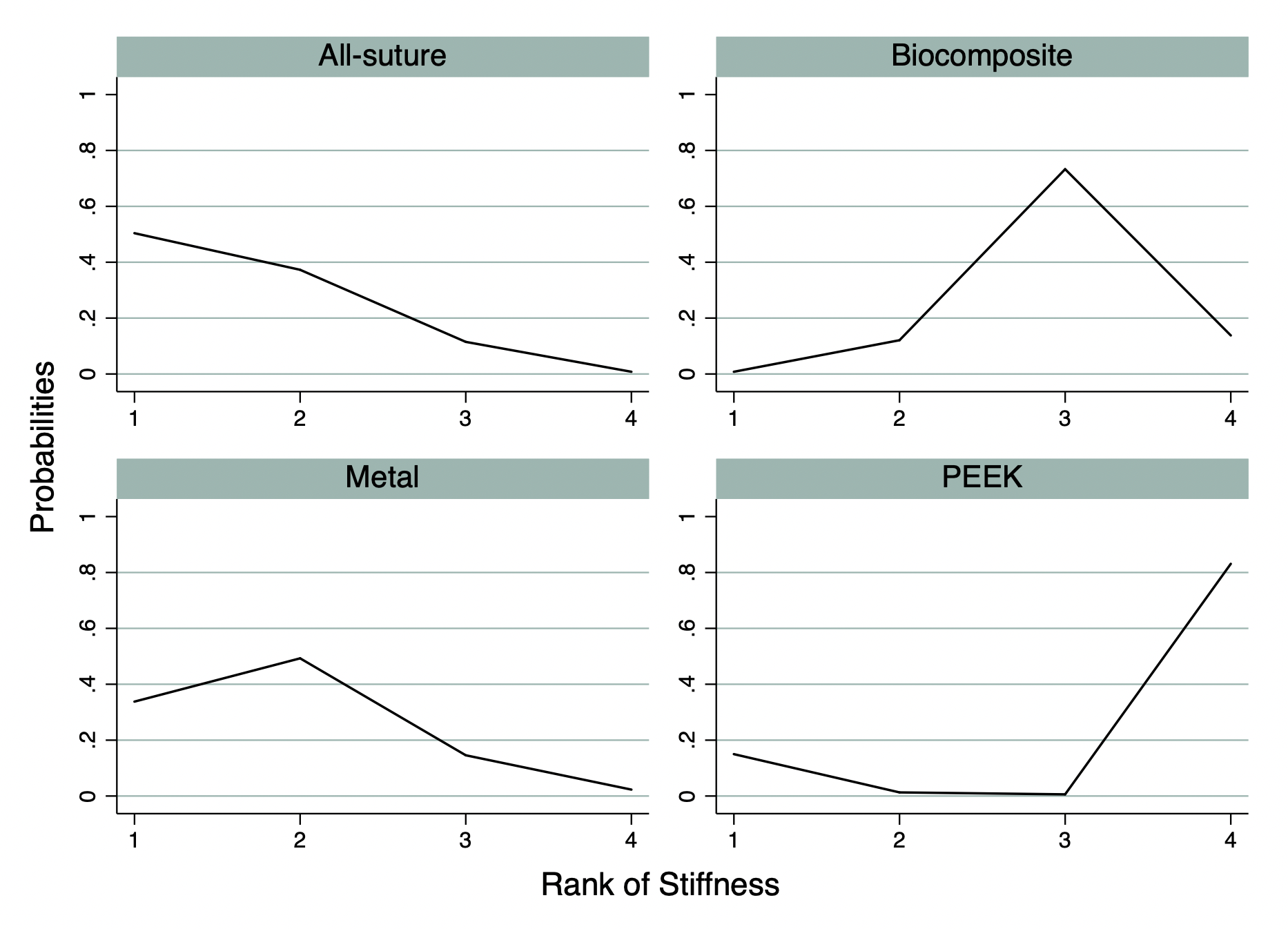 | | | | |
| --- | --- | --- | --- | --- |
| Ranking\Treatment | Metal | All-suture | Biocomposite | PEEK |
| Best | 33.8 | 50.4 | 0.8 | 15.0 |
| Mean Rank | 1.9 | 1.6 | 3.0 | 3.5 |
| SUCRA | 71.5 | 79.1 | 33.3 | 16.1 |

Abbreviations: PEEK, polyetheretherketone; SUCRA, the surface under the cumulative ranking.

###

## 9.3. Displacement

### 9.3.1. Displacement in league table and pairwise

eTable 9.3.1 Displacement in league table and pairwise

| **Metal** | 1.600  (0.462, 2.738) | 1.674  (0.879, 2.469) | - |
| --- | --- | --- | --- |
| -1.59  (-2.72, -0.46) | **All-suture** | 0.100  (-1.069, 1.269) | 0.746  (-0.957, 2.450) |
| -1.67  (-2.47, -0.88) | -0.09 (-1.25, 1.08) | **Biocomposite** | - |
| -2.57  (-4.22, -0.92) | -0.98  (-2.19, 0.22) | 0.90  (-2.57, 0.78) | **PEEK** |

Abbreviations: PEEK, polyetheretherketone

*Results of the network meta-analysis are presented in the left lower half. Weighted mean differences and 95% confidence interval were presented.

*Results of the pairwise meta-analysis are presented in the right upper half. Weighted mean differences and 95% confidence interval were presented.

eTable 9.3.2 Significant test of ES: Displacements


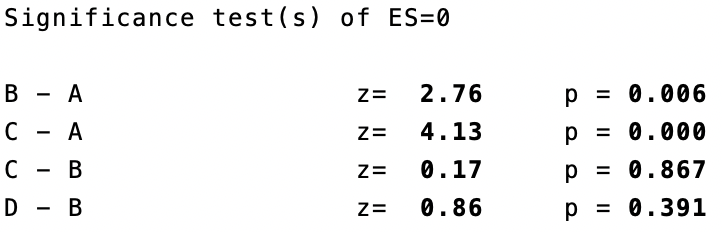


*A: Metal anchor; B: All-suture anchor; C: Biocomposite anchor; D: PEEK anchor.

### 9.3.2. LEAST Displacement in relative ranking probability

eTable 9.3.3 LEAST Displacement in relative ranking probability

| 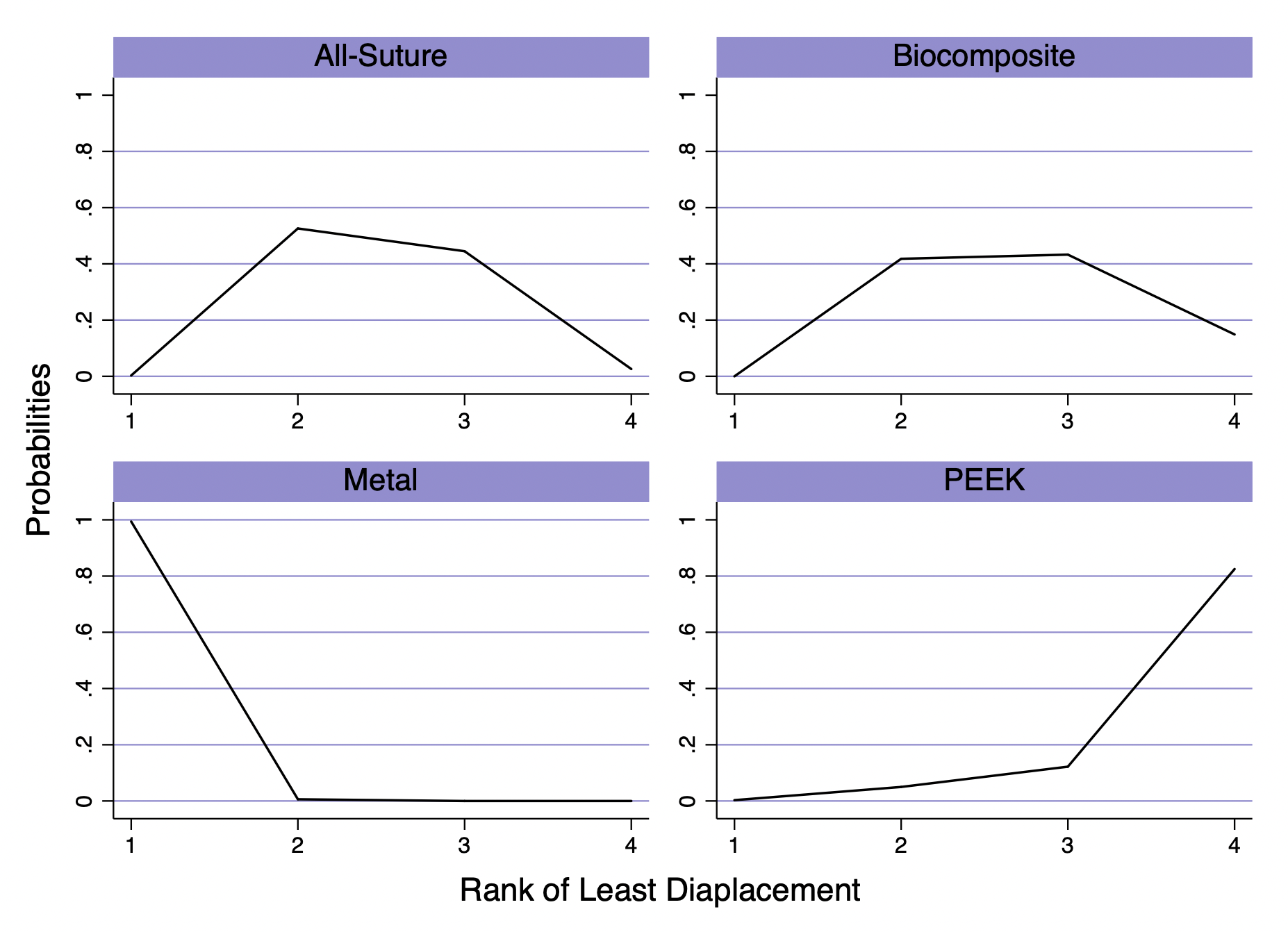 | | | | |
| --- | --- | --- | --- | --- |
| Ranking\Treatment | Metal | All-suture | Biocomposite | PEEK |
| Best | 99.4 | 0.3 | 0.0 | 0.3 |
| Mean Rank | 1.0 | 2.5 | 2.7 | 3.8 |
| SUCRA | 99.8 | 50.2 | 42.3 | 7.7 |

Abbreviations: PEEK, polyetheretherketone; SUCRA, the surface under the cumulative ranking.

## 9.4. Failure mode: Anchor pullout

### 9.4.1. Anchor pullout in league table and pairwise

eTable 9.4.1 Anchor pullout in league table and pairwise

| **Metal** | - | 0.247  (0.000, 129.945) | 1.000  (0.017, 59.993) |
| --- | --- | --- | --- |
| 0.05  (0.00, 8.71) | **All-suture** | - | 0.201  (0.026, 1.577) |
| 2.33  (0.07, 74.33) | 47.13 (0.49, 4534.58) | **Biocomposite** | 19.944  (1.104, 360.213) |
| 0.23  (0.00, 16.03) | 4.69  (0.24, 89.96) | 0.10  (0.00, 3.27) | **PEEK** |

Abbreviations: PEEK, polyetheretherketone

*Results of the network meta-analysis are presented in the left lower half. Weighted mean differences and 95% confidence interval were presented.

*Results of the pairwise meta-analysis are presented in the right upper half. Weighted mean differences and 95% confidence interval were presented.

eTable 9.4.2 Significant test of ES: Anchor Pullout


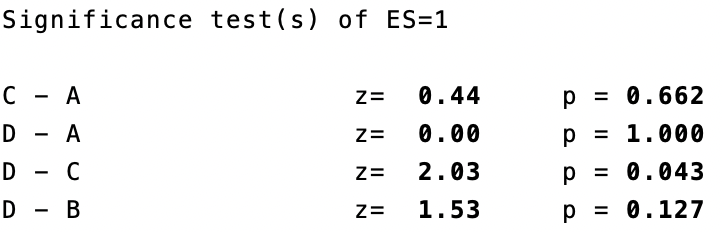


*A: Metal anchor; B: All-suture anchor; C: Biocomposite anchor; D: PEEK anchor.

### 9.4.2. LEAST Anchor pullout in relative ranking probability

eTable 9.4.3 LEAST Anchor pullout in relative ranking probability

| 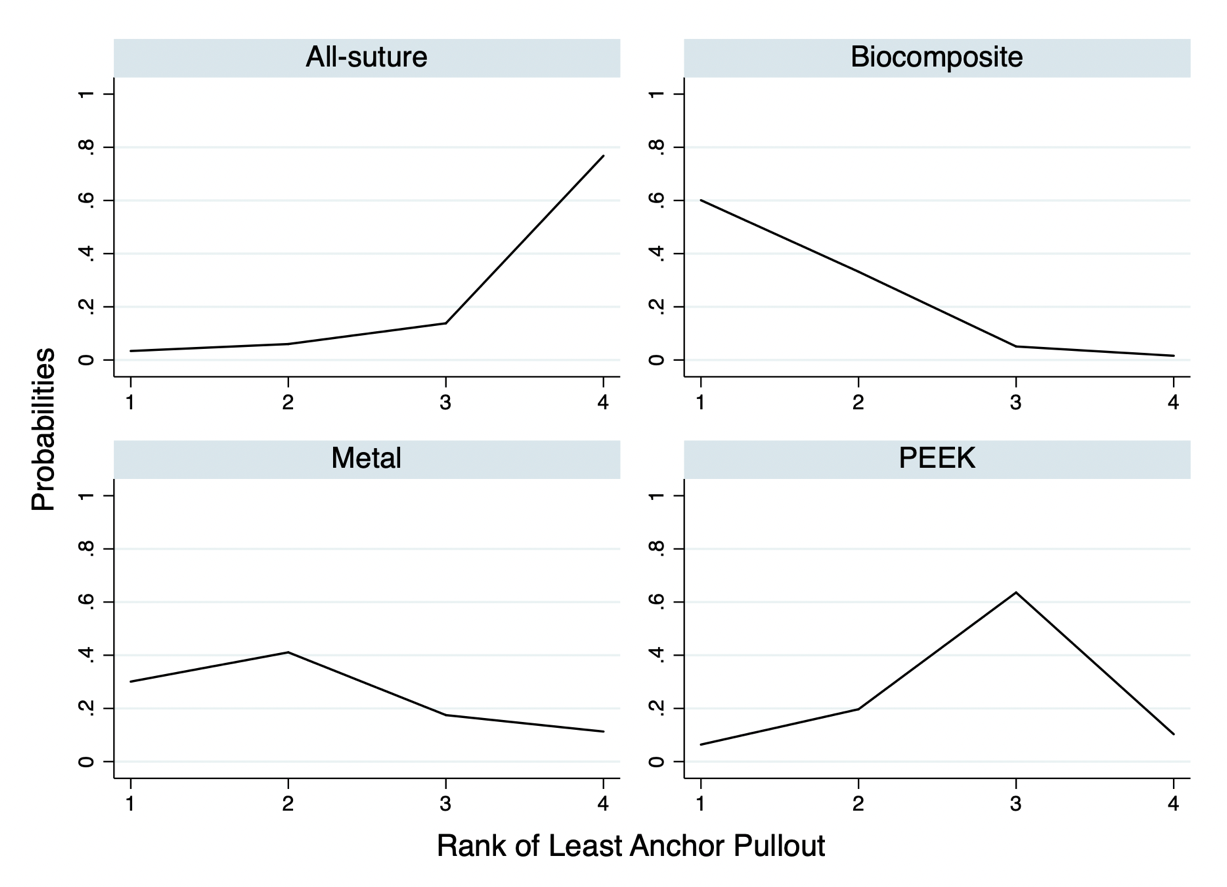 | | | | |
| --- | --- | --- | --- | --- |
| Ranking\Treatment | Metal | All-suture | Biocomposite | PEEK |
| Best | 30.1 | 3.4 | 60.1 | 6.4 |
| Mean Rank | 2.1 | 3.6 | 1.5 | 2.8 |
| SUCRA | 63.3 | 12.0 | 83.9 | 40.9 |

Abbreviations: PEEK, polyetheretherketone; SUCRA, the surface under the cumulative ranking.

## 9.5. Failure mode: Eyelet breakage

### 9.5.1. Eyelet breakage in league table and pairwise

eTable 9.5.1 Eyelet breakage in league table and pairwise

| **Metal** | - | 4.048  (0.008, 2129.479) | 1.000  (0.017, 59.993) |
| --- | --- | --- | --- |
| 6.27  (0.04, 996.13) | **All-suture** | - | 1.871  (0.206, 16.973) |
| 0.48  (0.02, 13.69) | 0.08 (0.00, 6.94) | **Biocomposite** | 0.062  (0.002, 2.096) |
| 3.53  (0.06, 214.92) | 0.56  (0.03, 10.77) | 7.34  (0.24, 222.86) | **PEEK** |

Abbreviations: PEEK, polyetheretherketone

*Results of the network meta-analysis are presented in the left lower half. Weighted mean differences and 95% confidence interval were presented.

*Results of the pairwise meta-analysis are presented in the right upper half. Weighted mean differences and 95% confidence interval were presented.

eTable 9.5.2 Significant test of ES: Eyelet Breakage


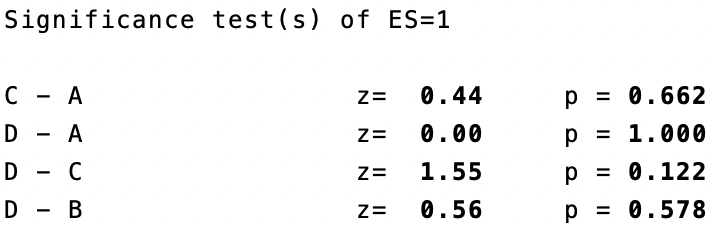


*A: Metal anchor; B: All-suture anchor; C: Biocomposite anchor; D: PEEK anchor.

### 9.5.2. LEAST Eyelet breakage in relative ranking probability

eTable 9.5.3 LEAST Eyelet breakage in relative ranking probability

| 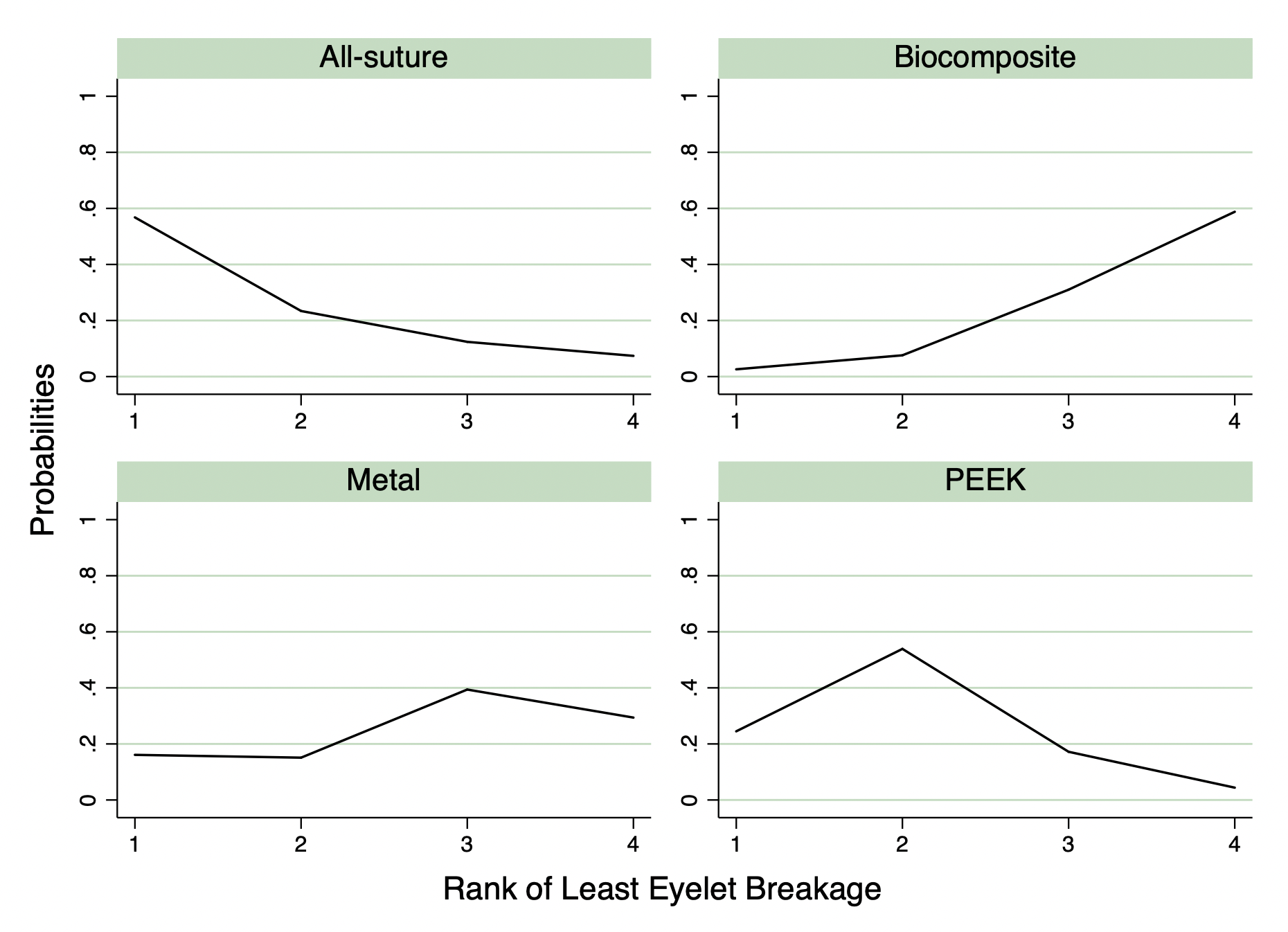 | | | | |
| --- | --- | --- | --- | --- |
| Ranking\Treatment | Metal | All-suture | Biocomposite | PEEK |
| Best | 16.1 | 56.8 | 2.6 | 24.5 |
| Mean Rank | 2.8 | 1.7 | 3.5 | 2.0 |
| SUCRA | 39.3 | 76.5 | 18.0 | 66.2 |

Abbreviations: PEEK, polyetheretherketone; SUCRA, the surface under the cumulative ranking.

## 9.6. Failure mode: Suture breakage

### 9.6.1. Suture breakage in league table and pairwise

eTable 9.6.1 Suture breakage in league table and pairwise

| **Metal** | - | 1.000  (0.056, 17.916) | 1.000  (0.017, 59.993) |
| --- | --- | --- | --- |
| 3.76  (0.08, 186.20) | **All-suture** | - | 3.766  (0.488, 29.050) |
| 1.00  (0.06, 16.20) | 0.27 (0.01, 8.26) | **Biocomposite** | 1.000  (0.057, 17.466) |
| 1.00  (0.04, 27.76) | 0.27  (0.03, 2.05) | 1.00  (0.06, 15.86) | **PEEK** |

Abbreviations: PEEK, polyetheretherketone

*Results of the network meta-analysis are presented in the left lower half. Odd ratio and 95% confidence interval were presented. Odds ratio less than one favor the column-defining treatment.

*Results of the pairwise meta-analysis are presented in the right upper half. Odd ratio and 95% confidence interval were presented. Odds ratio less than one favor the column-defining treatment.

eTable 9.6.2 Significant test of ES: Suture Breakage


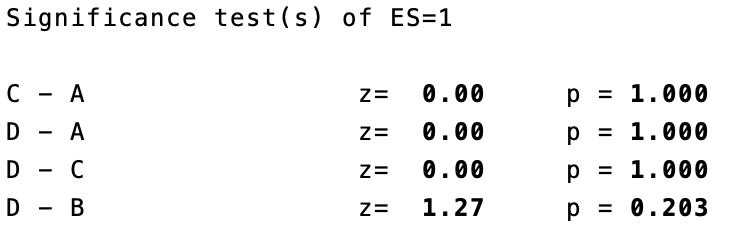


*A: Metal anchor; B: All-suture anchor; C: Biocomposite anchor; D: PEEK anchor.

### 9.6.2. LEAST Suture breakage in relative ranking probability

eTable 9.6.3 LEAST Suture breakage in relative ranking probability

| 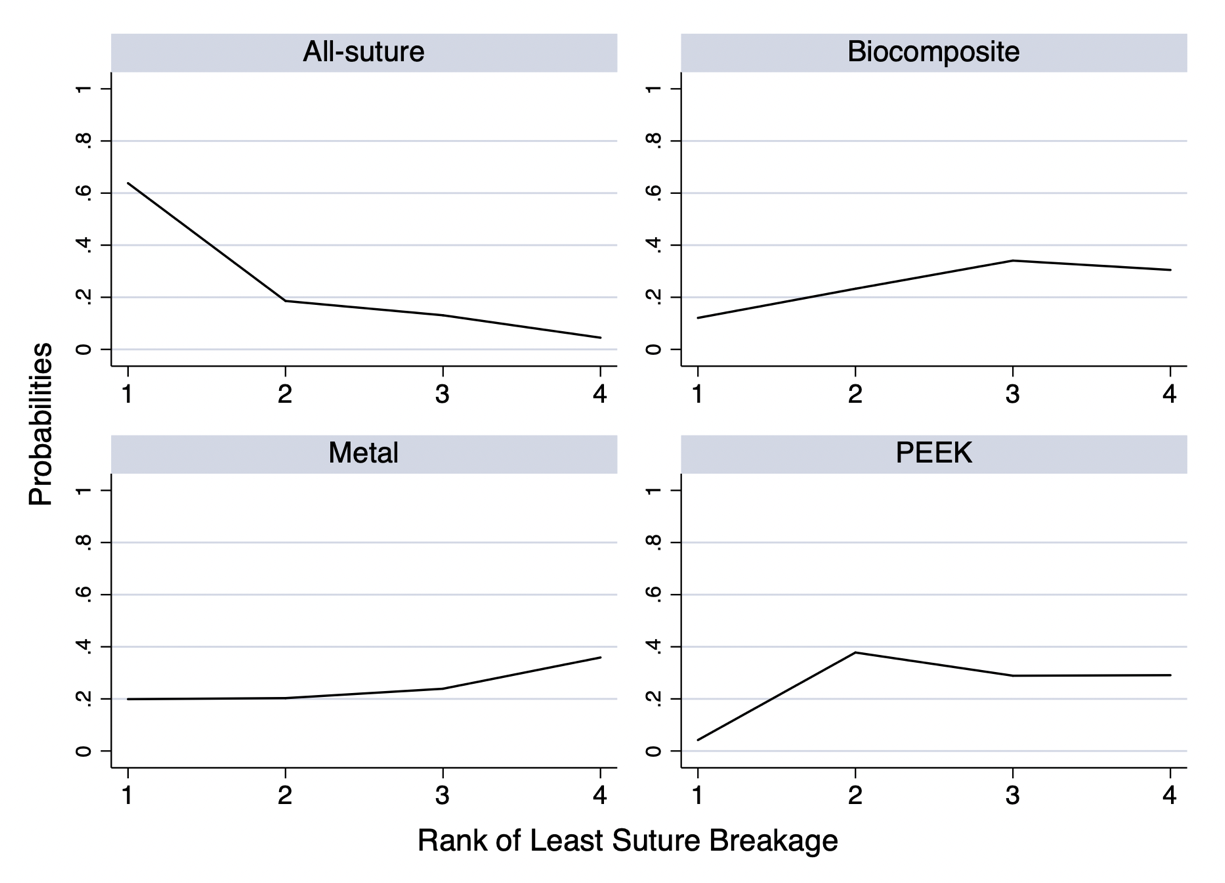 | | | | |
| --- | --- | --- | --- | --- |
| Ranking\Treatment | Metal | All-suture | Biocomposite | PEEK |
| Best | 19.9 | 63.8 | 12.1 | 4.2 |
| Mean Rank | 2.8 | 1.6 | 2.8 | 2.8 |
| SUCRA | 41.4 | 80.6 | 39.0 | 39.0 |

Abbreviations: PEEK, polyetheretherketone; SUCRA, the surface under the cumulative ranking.

##

## 9.7. Interval plot


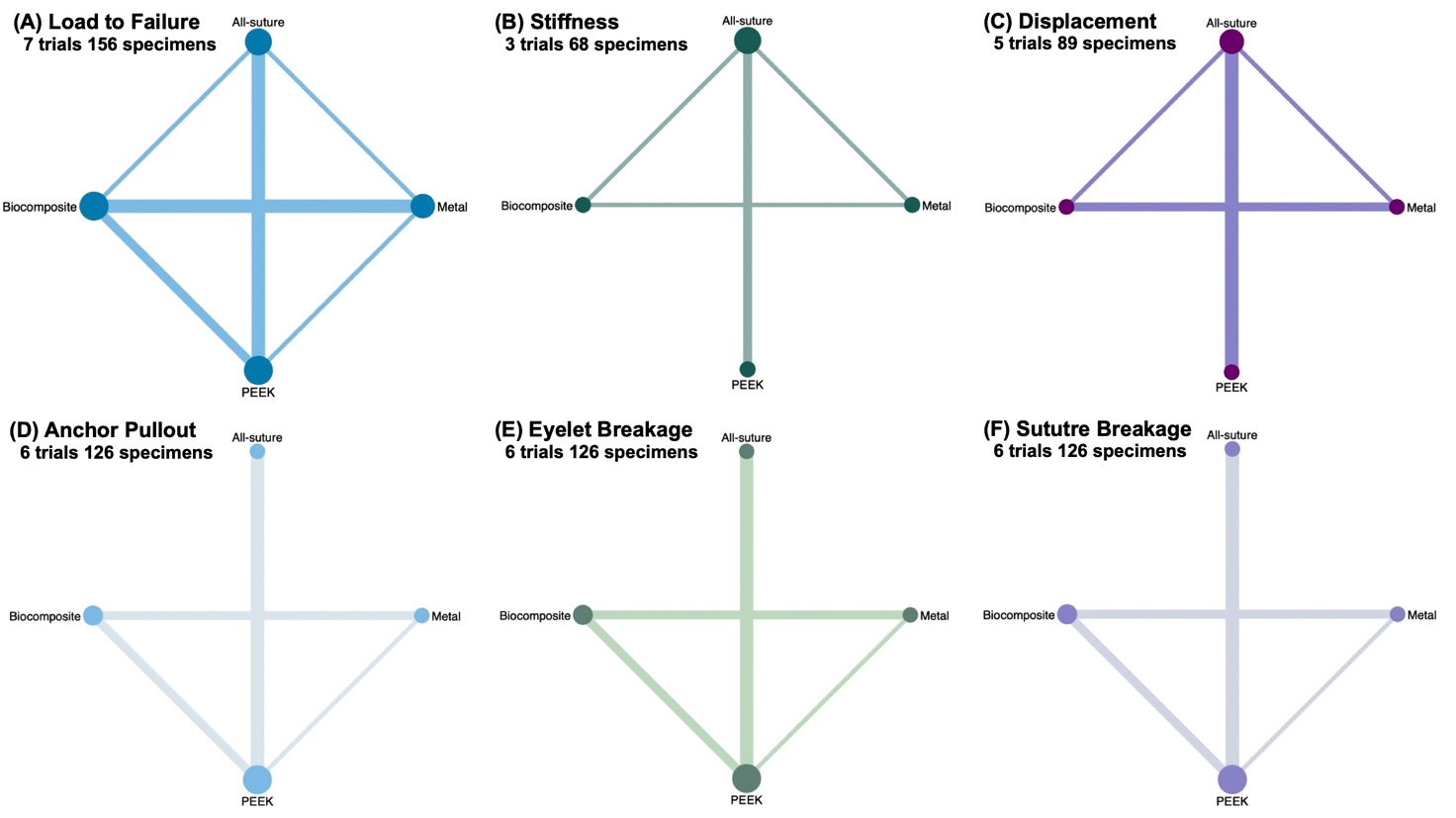


eFigure.9.1. Network geometry

1. Load to Failure, (B)Stiffness, (C)Displacement, (D) Anchor Pullout, (E) Eyelet Breakage, (F) Suture Breakage.


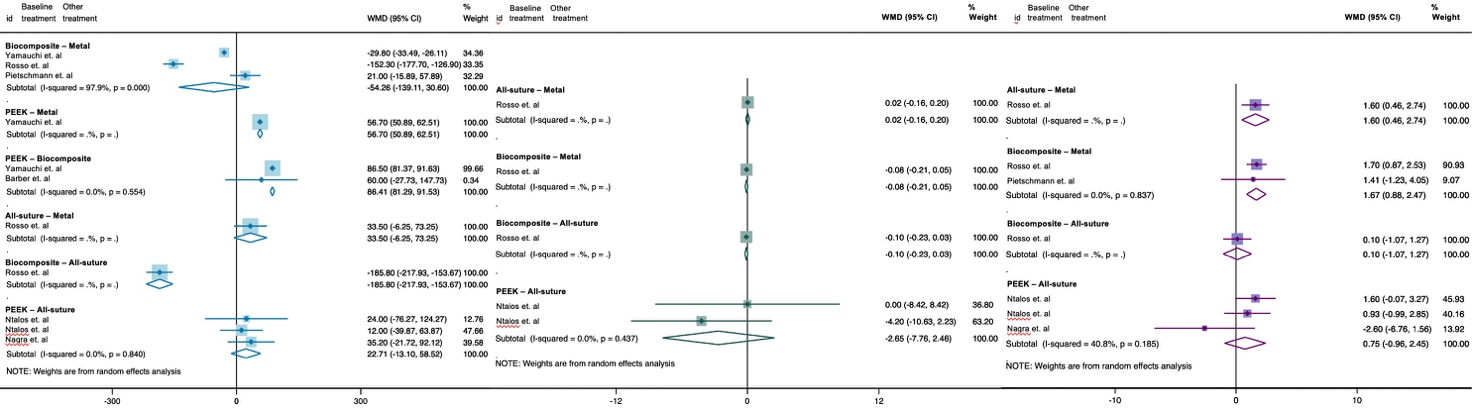


eFigure 9.2. Pairwise forest plot

1. Load to Failure, (B)Stiffness, (C)Displacement, (D) Anchor Pullout, (E) Eyelet Breakage, (F) Suture Breakage. (OR, odds ration; *, p<0.05; **, p<0.01; L, low confidence rating；VL, very low confidence rating)

**
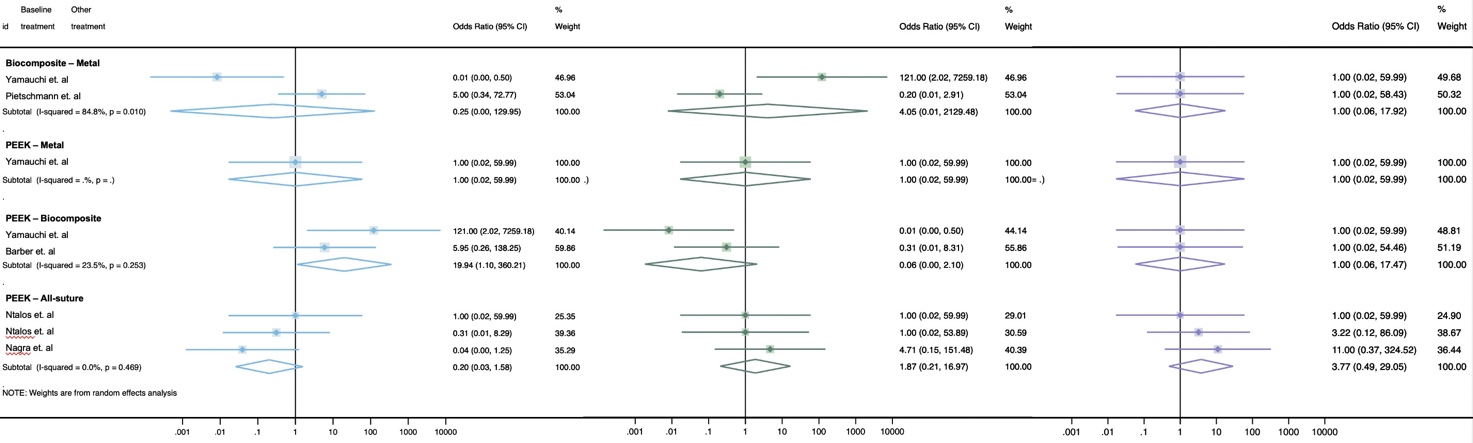
**

eFigure 9.3. Pairwise forest plot

1. Load to Failure, (B)Stiffness, (C)Displacement, (D) Anchor Pullout, (E) Eyelet Breakage, (F) Suture Breakage. (OR, odds ration; *, p<0.05; **, p<0.01; L, low confidence rating；VL, very low confidence rating)

# Appendix 10: Publication bias

## 10.1. Publication bias: Load to Failure


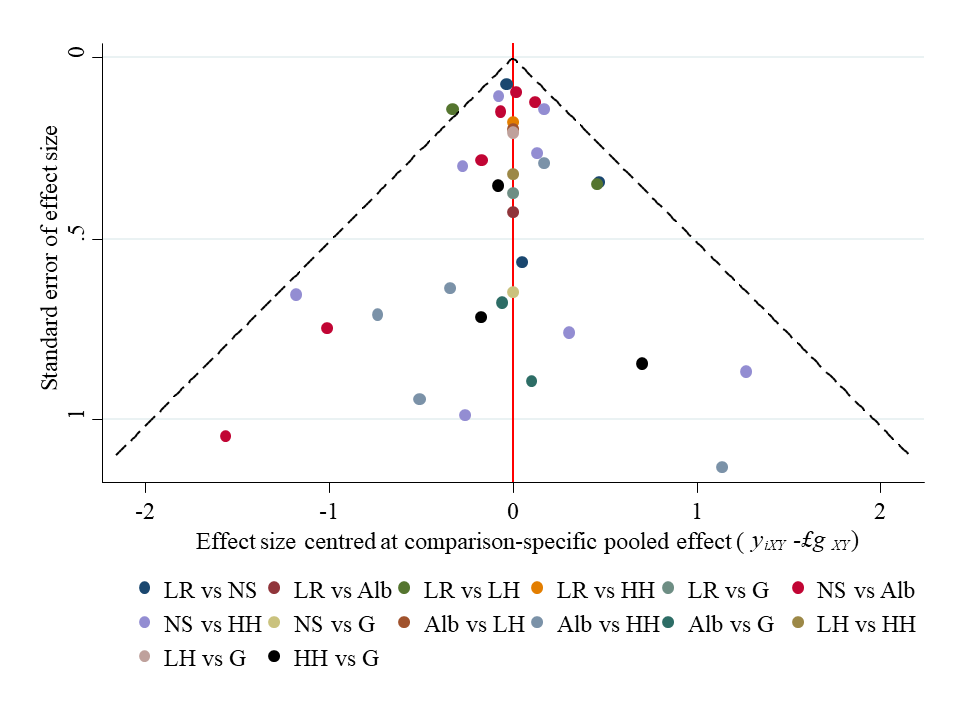


*PEEK, polyetheretherketone.

**
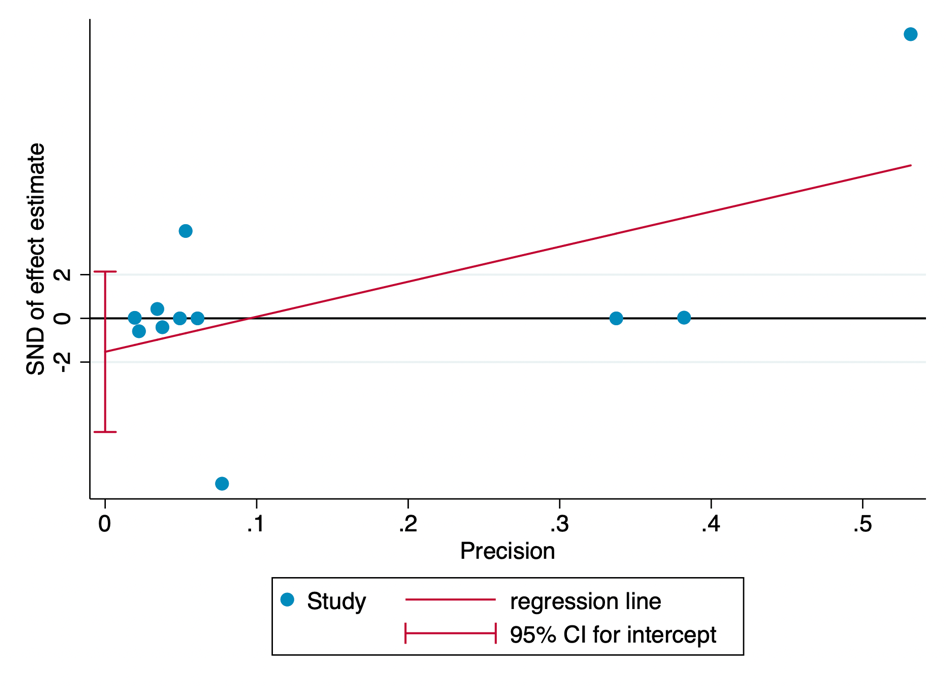
**

| Egger's test for small-study effects: | | | | | | |
| --- | --- | --- | --- | --- | --- | --- |
| Std_Eff | Coef. | Std. Err. | t | P>t | [95% Conf. | Interval] |
| slope | 16.04589 | 7.185384 | 2.23 | 0.052 | -0.2085762 | 32.30036 |
| bias | -1.53025 | 1.622464 | -0.94 | 0.370 | -5.200518 | 2.140018 |
| Test of H0: no small-study effects= P = 0.370 | | | | | | |

## 10.2. Publication bias: Stiffness


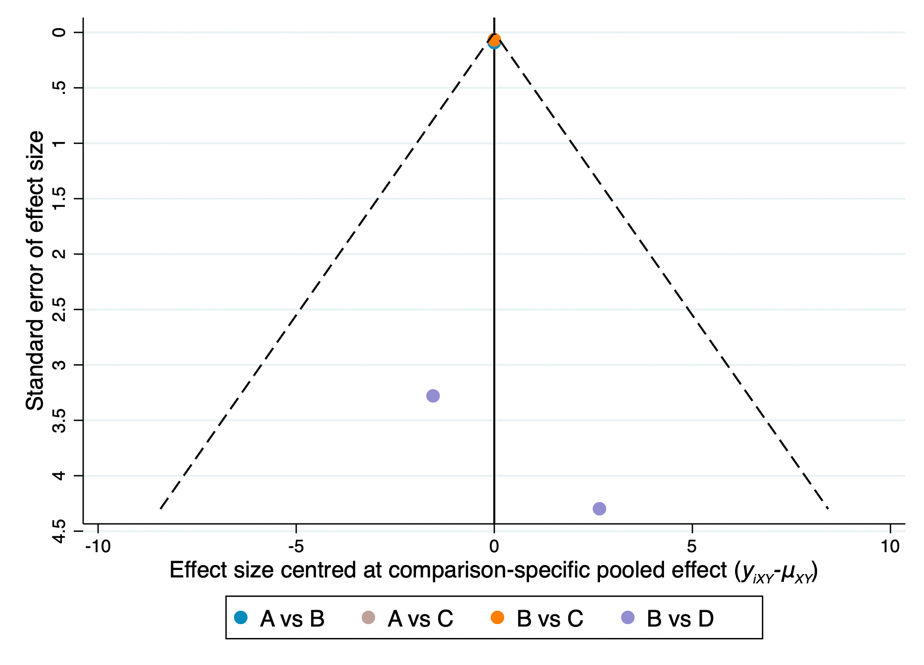


* PEEK, polyetheretherketone.


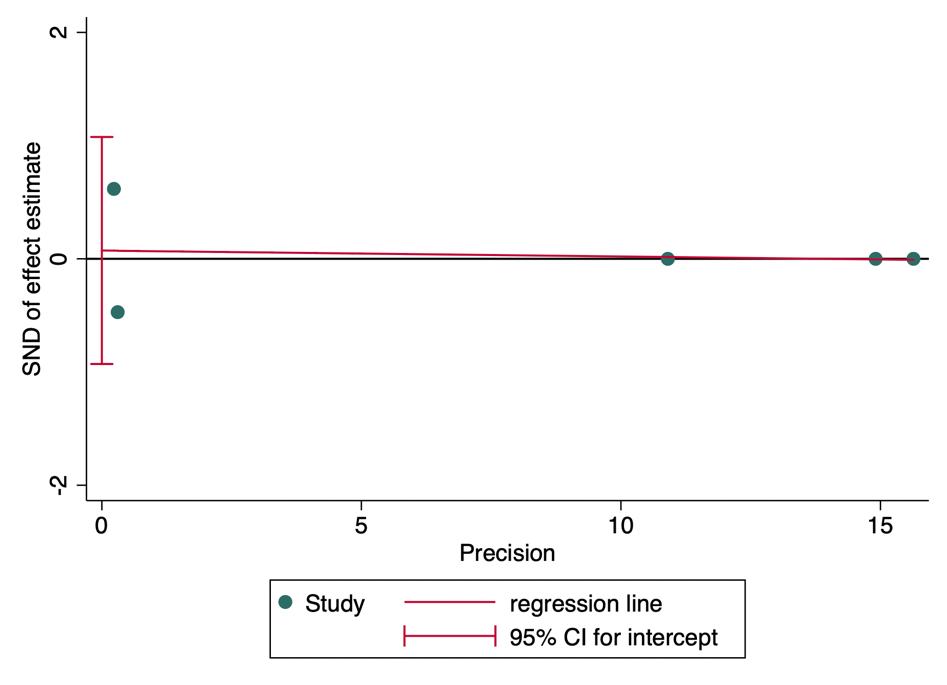


| Egger's test for small-study effects: | | | | | | |
| --- | --- | --- | --- | --- | --- | --- |
| Std_Eff | Coef. | Std. Err. | t | P>t | [95% Conf. | Interval] |
| slope | -0.0052707 | 0.0291084 | -0.18 | 0.868 | -0.0979067 | 0.0873653 |
| bias | 0.0735353 | 0.316064 | 0.23 | 0.830 | -0.9291389 | 1.07621 |
| Test of H0: no small-study effects P = 0.830 | | | | | | |

## 10.3. Publication bias: Displacement

**
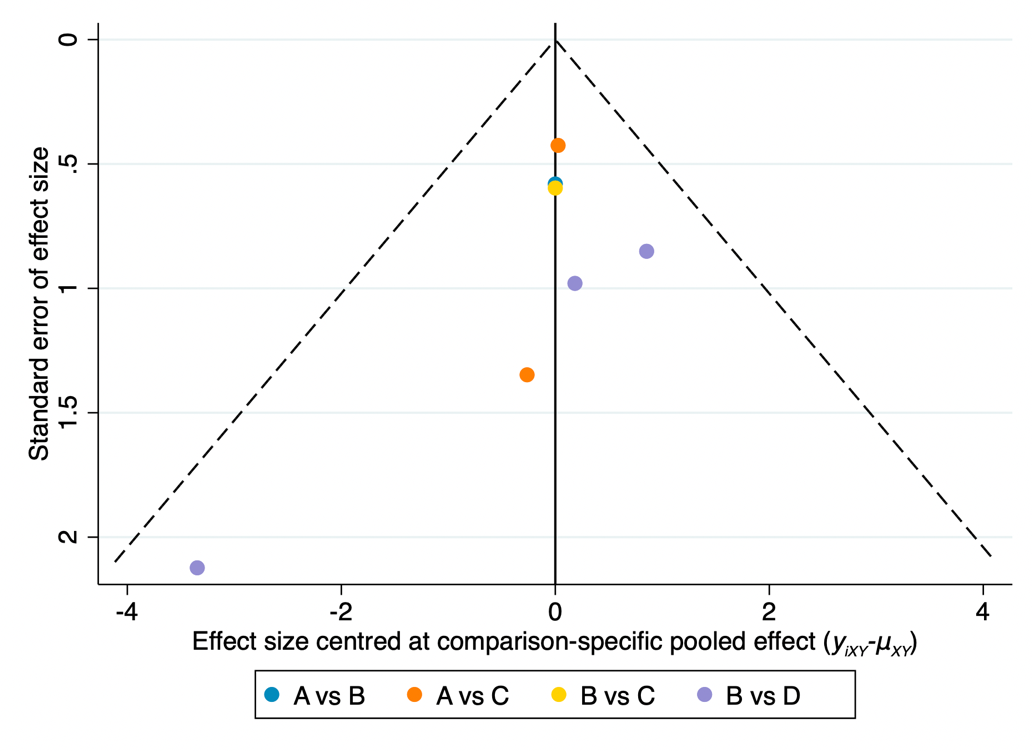
**

* PEEK, polyetheretherketone.

.
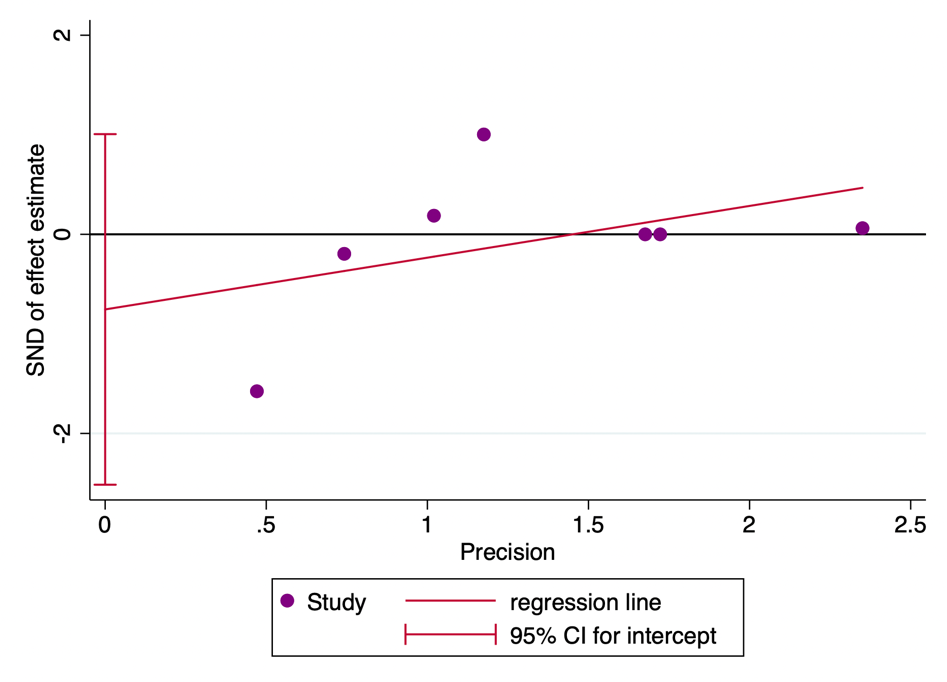


| Egger's test for small-study effects: | | | | | | |
| --- | --- | --- | --- | --- | --- | --- |
| Std_Eff | Coef. | Std. Err. | t | P>t | [95% Conf. | Interval] |
| slope | 0.519834 | 0.4759985 | 1.09 | 0.315 | -0.7037592 | 1.743427 |
| bias | -0.7543083 | 0.6849916 | -1.10 | 0.321 | -2.515135 | 1.006519 |
| Test of H0: no small-study effects P = 0.321 | | | | | | |

## 10.4. Publication bias: Anchor Pullout

**
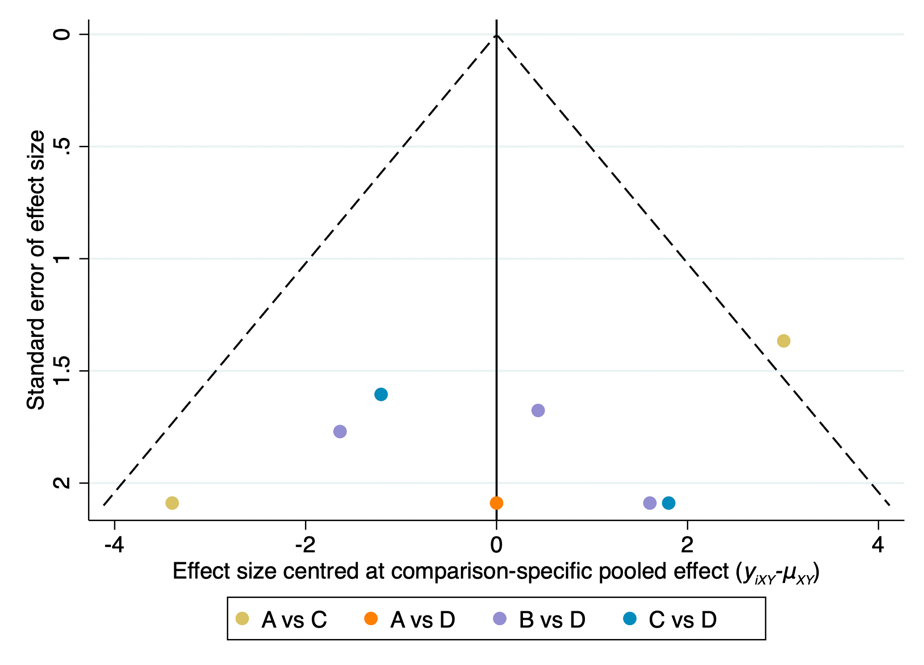
**

* PEEK, polyetheretherketone.

**
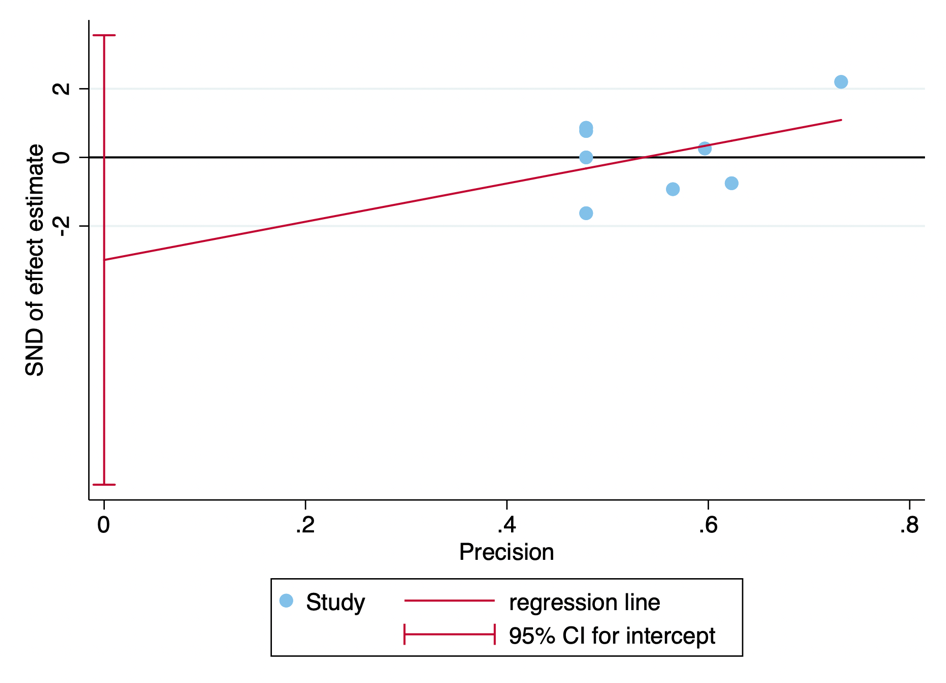
**

| Egger's test for small-study effects: | | | | | | |
| --- | --- | --- | --- | --- | --- | --- |
| Std_Eff | Coef. | Std. Err. | t | P>t | [95% Conf. | Interval] |
| slope | 5.574263 | 4.773621 | 1.17 | 0.287 | -6.106366 | 17.25489 |
| bias | -2.989298 | 2.676882 | -1.12 | 0.307 | -9.539393 | 3.560796 |
| Test of H0: no small-study effects P = 0.307 | | | | | | |

## 10.5. Publication bias: Eyelet Breakage


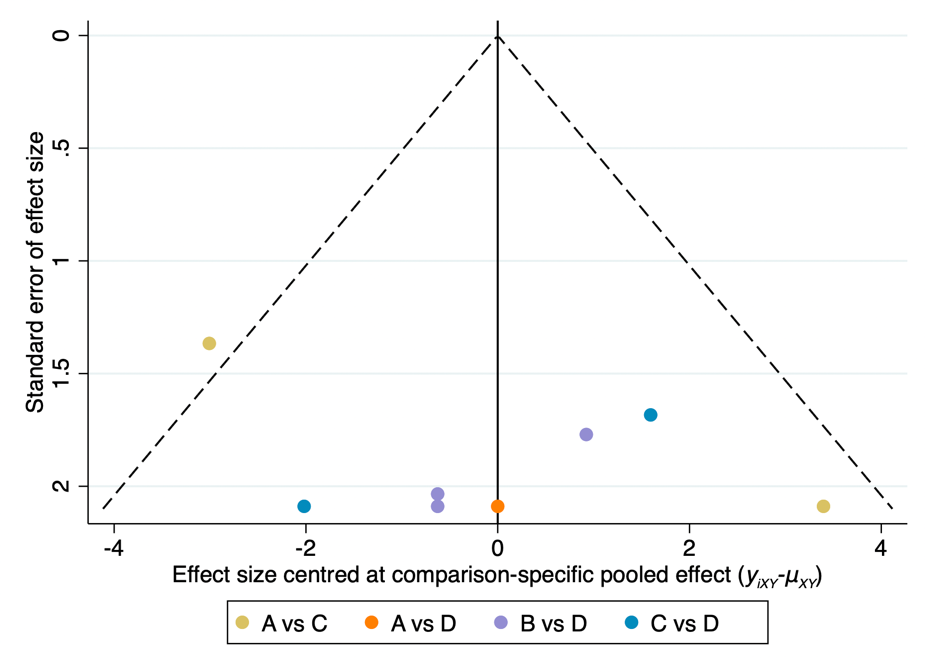


* PEEK, polyetheretherketone.


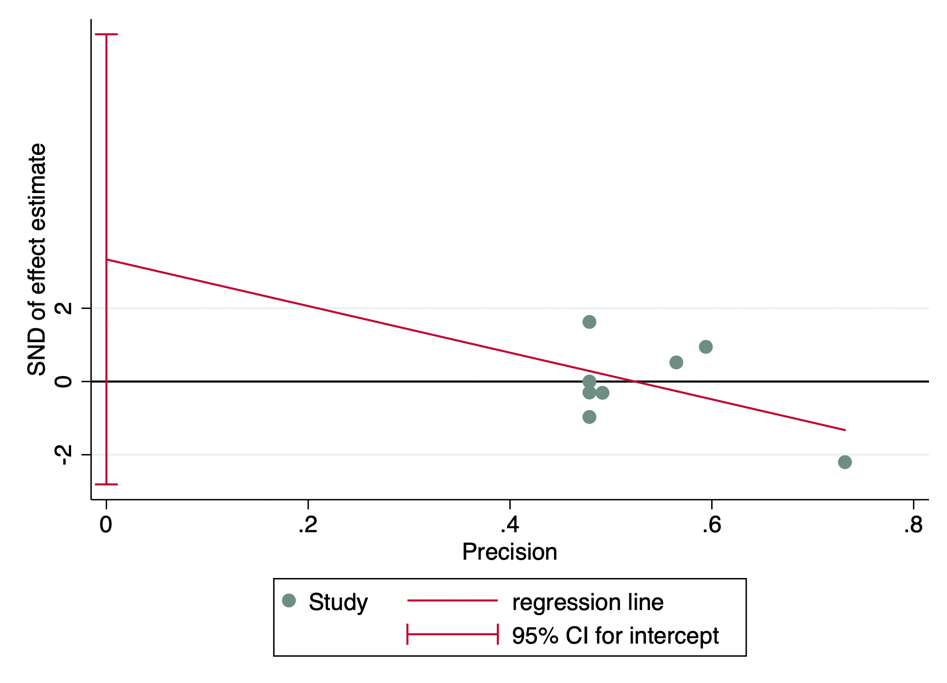


| Egger's test for small-study effects: | | | | | | |
| --- | --- | --- | --- | --- | --- | --- |
| Std_Eff | Coef. | Std. Err. | t | P>t | [95% Conf. | Interval] |
| slope | -6.369989 | 4.618735 | -1.38 | 0.217 | -17.67163 | 4.931649 |
| bias | 3.336797 | 2.51181 | 1.33 | 0.232 | -2.80938 | 9.482974 |
| Test of H0: no small-study effects P = 0.232 | | | | | | |

## 10.6. Publication bias: Suture Breakage


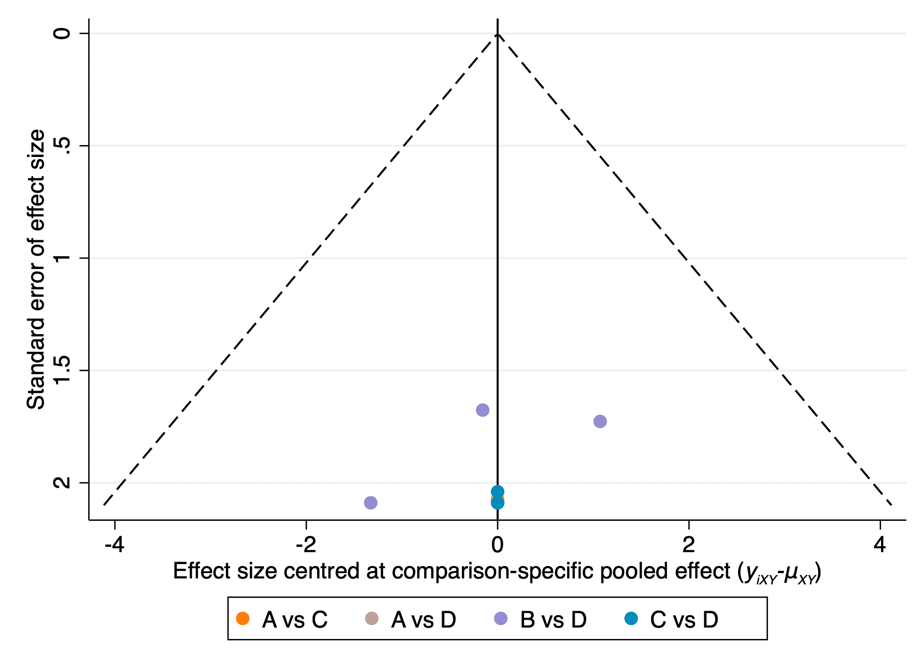


* PEEK, polyetheretherketone.


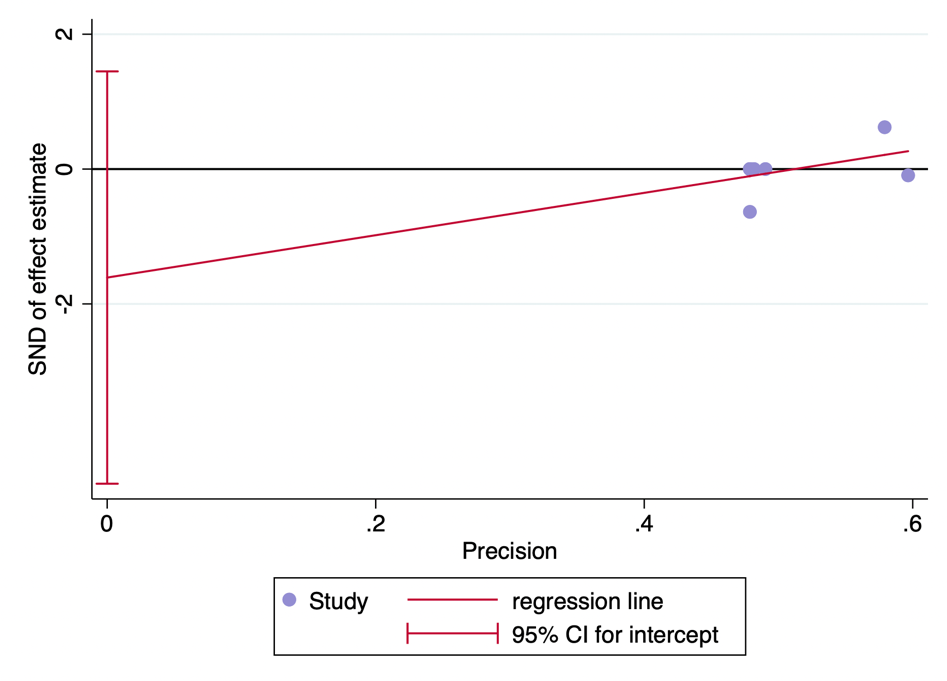


| Egger's test for small-study effects: | | | | | | |
| --- | --- | --- | --- | --- | --- | --- |
| Std_Eff | Coef. | Std. Err. | t | P>t | [95% Conf. | Interval] |
| slope | 3.142135 | 2.4502 | 1.28 | 0.247 | -2.853288 | 9.137559 |
| bias | -1.609079 | 1.249503 | -1.29 | 0.245 | -4.666501 | 1.448344 |
| Test of H0: no small-study effects P = 0.245 | | | | | | |

# Appendix 11: Inconsistency

| **Outcome** | **Fit design-by-treatment interaction model** | **Explore Loop inconsistency** |
| --- | --- | --- |
| **Load to Failure** | p=0.000 | p=0.6289 |
| **Stiffness** | p=0.3086 | p=0.3086 |
| **Displacement** | p=0.8375 | p=0.1098 |
| **Failure mode: Anchor pullout** | p=0.0354 | p=0.7733 |
| **Failure mode: Eyelet breakage** | p=0.0321 | p=0.6535 |
| **Failure mode: Suture breakage** | p=1.000 | p=0.9998 |

## 11.1. Inconsistency: Load to Failure

eTable 11.1.1 Inconsistency between direct and indirect evidence: Load to Failure

| Side | Direct | | Indirect | | Difference | | |
| --- | --- | --- | --- | --- | --- | --- | --- |
|  | Coef. | Std. Err. | Coef. | Std. Err. | Coef. | Std. Err. | P>z |
| A B | 32.02927 | 66.53951 | 70.64718 | 66.74688 | -38.61792 | 94.18491 | 0.682 |
| A C | -54.6768 | 36.18772 | 68.07405 | 172.6618 | -122.7509 | 176.31 | 0.486 |
| A D | 56.70998 | 65.20575 | 55.525 | 63.21345 | 1.184975 | 90.81528 | 0.990 |
| B C | -190.5145 | 39.08194 | -26.44847 | 38.50831 | -164.066 | 54.23255 | **0.002** |
| B D | 23.55784 | 40.03344 | -52.11511 | 69.25724 | 75.67296 | 79.9956 | 0.344 |
| C D | 76.34987 | 44.94707 | 167.7332 | 64.53818 | -91.38332 | 78.59954 | 0.245 |

*A: Metal, B: All-suture; C: Biocomposite, D: PEEK.

eTable 11.1.2 Design inconsistency: Load to Failure


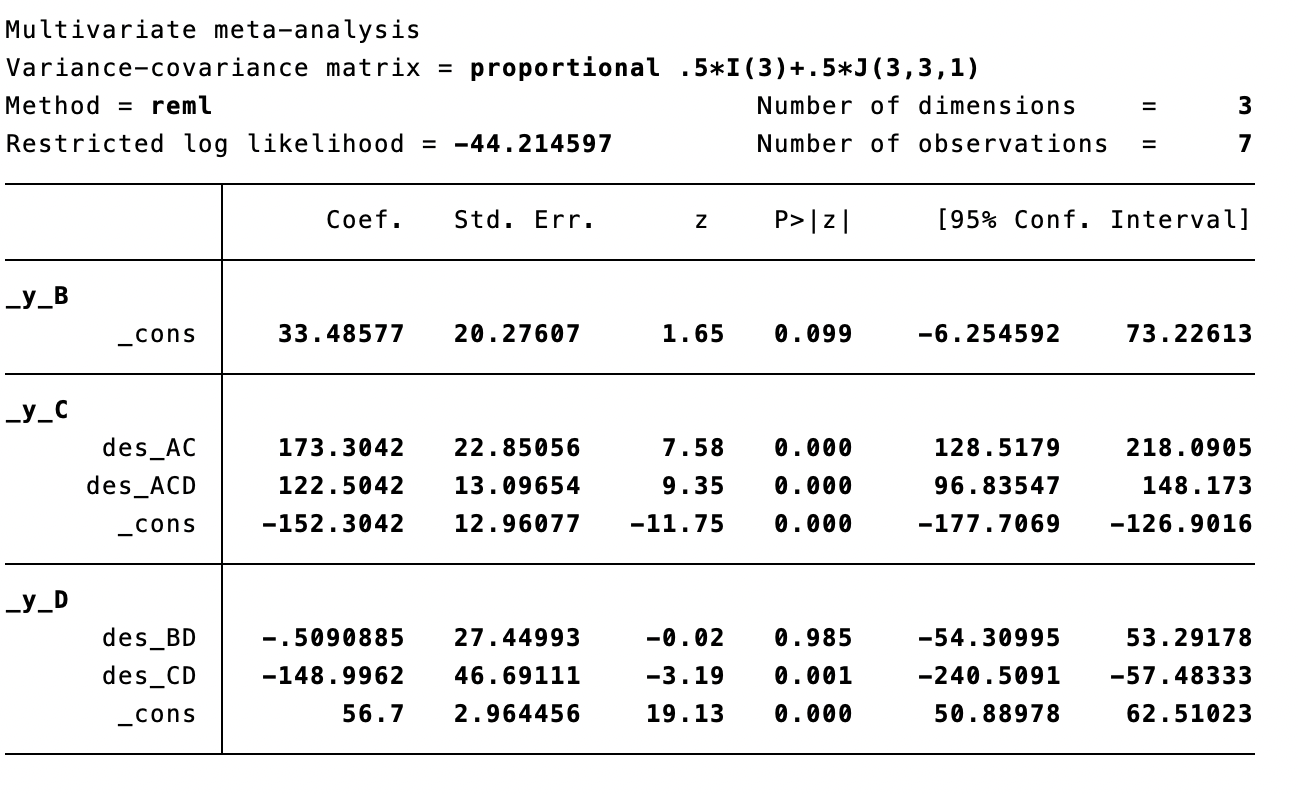


* A: Metal, B: All-suture; C: Biocomposite, D: PEEK.

## 11.2. Inconsistency: Stiffness

eTable 11.2.1 Inconsistency between direct and indirect evidence: Stiffness

| Side | Direct | | Indirect | | Difference | | |
| --- | --- | --- | --- | --- | --- | --- | --- |
|  | Coef. | Std. Err. | Coef. | Std. Err. | Coef. | Std. Err. | P>z |
| A B | 0.02 | 0.0917061 | 2.072712 | 149.7793 | -2.052712 | 149.7793 | 0.989 |
| A C | -0.08 | 0.0639531 | 4.025425 | 299.5586 | -4.105425 | 299.5586 | 0.989 |
| B C | -1 | 0.067082 | -4.205425 | 299.5586 | 4.105425 | 299.5586 | 0.989 |
| B D | -2.654563 | 2.607072 | 1.450861 | 299.5303 | -4.105425 | 299.5586 | 0.989 |

*A: Metal, B: All-suture; C: Biocomposite, D: PEEK.

eTable 11.2.2 Design inconsistency: Stiffness


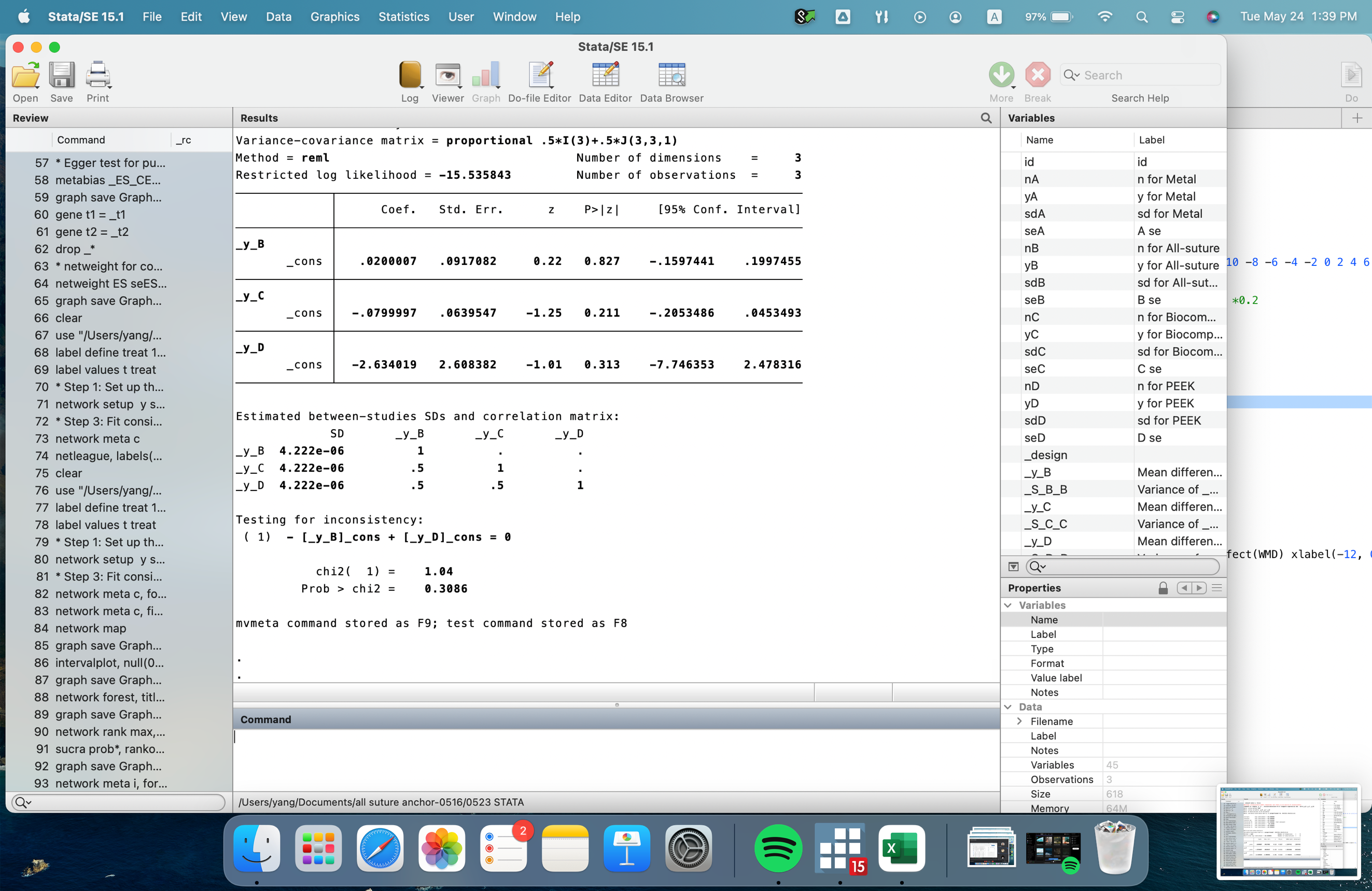


## 11.3. Inconsistency: Displacement

eTable 11.3.1 Inconsistency between direct and indirect evidence: Displacement

| Side | Direct | | Indirect | | Difference | | |
| --- | --- | --- | --- | --- | --- | --- | --- |
|  | Coef. | Std. Err. | Coef. | Std. Err. | Coef. | Std. Err. | P>z |
| A B | 1.599739 | 0.5804954 | 1.008039 | 2.81904 | 0.5917001 | 2.817136 | 0.834 |
| A C | 1.673711 | 0.4056992 | -3.104177 | 69.54708 | 4.777888 | 69.54801 | 0.945 |
| B C | 0.1003466 | 0.5966405 | -0.4704317 | 2.815528 | 0.5707783 | 2.823524 | 0.840 |
| B D | 0.9837974 | 0.6149504 | -3.794087 | 69.57884 | 4.777884 | 69.58158 | 0.945 |

*A: Metal, B: All-suture; C: Biocomposite, D: PEEK.

eTable 11.3.2 Design inconsistency: Displacement


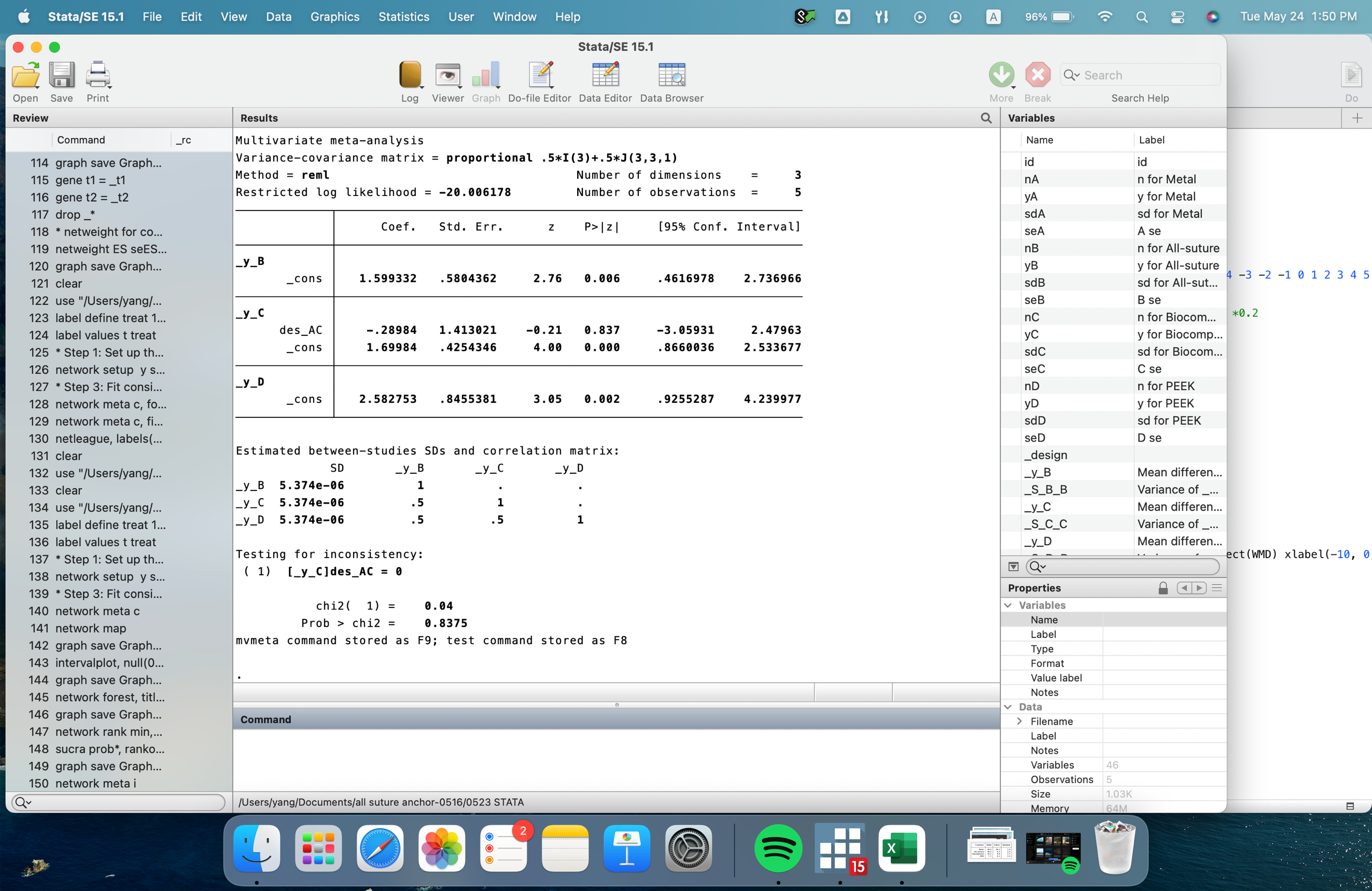


* A: Metal, B: All-suture; C: Biocomposite, D: PEEK.

## 11.4. Inconsistency: Anchor Pullout

eTable 11.4.1 Inconsistency between direct and indirect evidence: Anchor Pullout

| Side | Direct | | Indirect | | Difference | | |
| --- | --- | --- | --- | --- | --- | --- | --- |
|  | Coef. | Std. Err. | Coef. | Std. Err. | Coef. | Std. Err. | P>z |
| A C | -1.127632 | 2.085468 | 1.157034 | 7.787752 | -2.284666 | 8.059664 | 0.777 |
| A D | 3.90e-08 | 2.90609 | 3.548163 | 3.543011 | -3.548163 | 4.582389 | 0.439 |
| B D | -1.546486 | 1.507428 | 2.795785 | 104.0923 | -4.342271 | 104.1002 | 0.967 |
| C D | 2.901487 | 1.27258 | -7.978138 | 4.522071 | 10.87963 | 4.697651 | **0.021** |

*A: Metal, B: All-suture; C: Biocomposite, D: PEEK.

eTable 11.4.2 Design inconsistency: Anchor Pullout


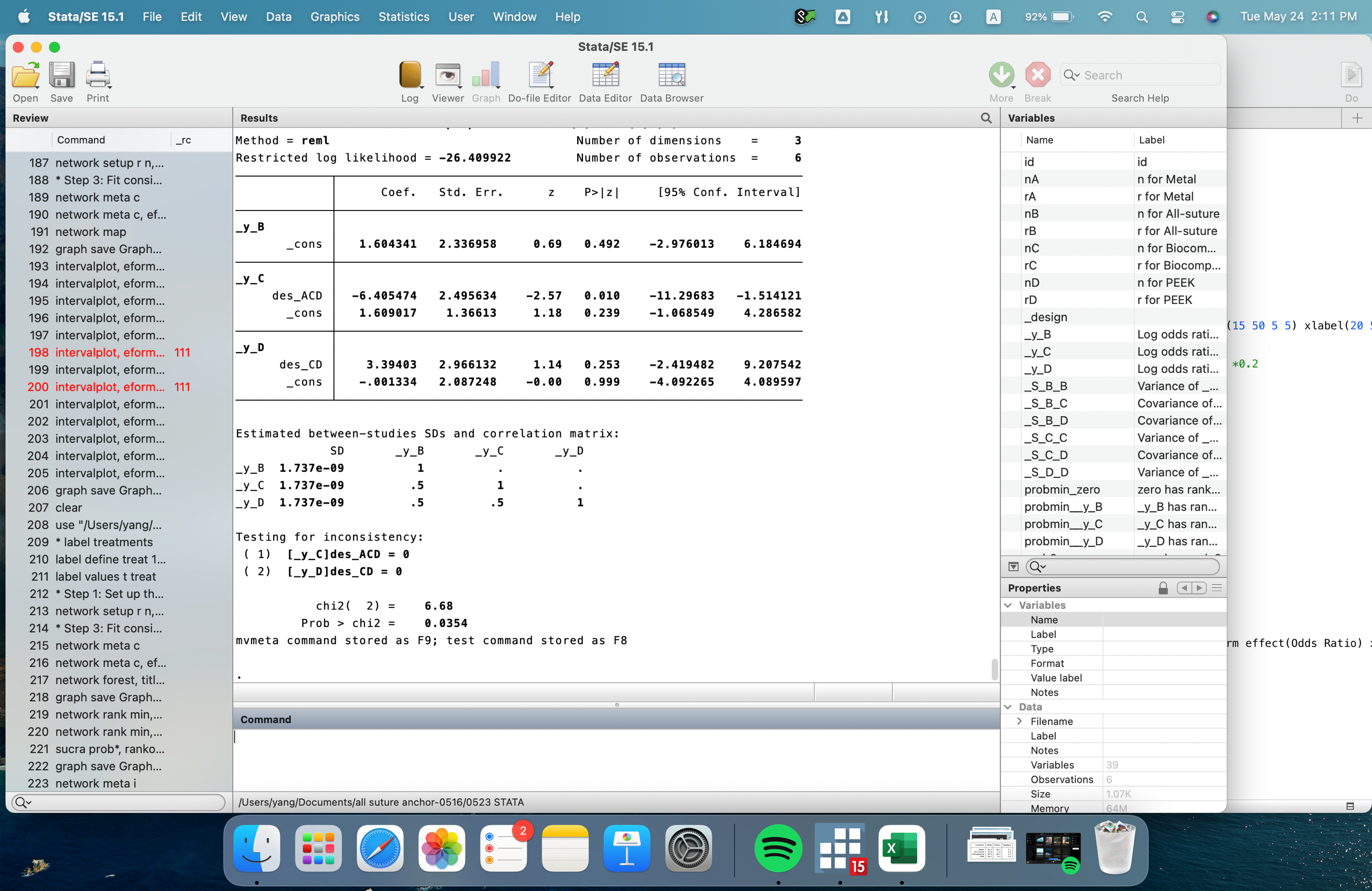


* A: Metal, B: All-suture; C: Biocomposite, D: PEEK.

## 11.5. Inconsistency: Eyelet Breakage

eTable 11.5.1 Inconsistency between direct and indirect evidence: Eyelet Breakage

| Side | Direct | | Indirect | | Difference | | |
| --- | --- | --- | --- | --- | --- | --- | --- |
|  | Coef. | Std. Err. | Coef. | Std. Err. | Coef. | Std. Err. | P>z |
| A C | 1.076817 | 1.98734 | -2.380328 | 7.496574 | 3.457145 | 7.753465 | 0.656 |
| A D | 4.23e-11 | 2.866785 | -3.041531 | 3.519959 | 3.041531 | 4.539666 | 0.503 |
| B D | 0.5761666 | 1.506843 | -2.500264 | 124.7561 | 3.076431 | 124.7633 | 0.980 |
| C D | -2.604695 | 1.310721 | 7.987224 | 4.525705 | -10.59192 | 4.711648 | **0.025** |

*A: Metal, B: All-suture; C: Biocomposite, D: PEEK.

eTable 11.5.2 Design inconsistency: Eyelet Breakage


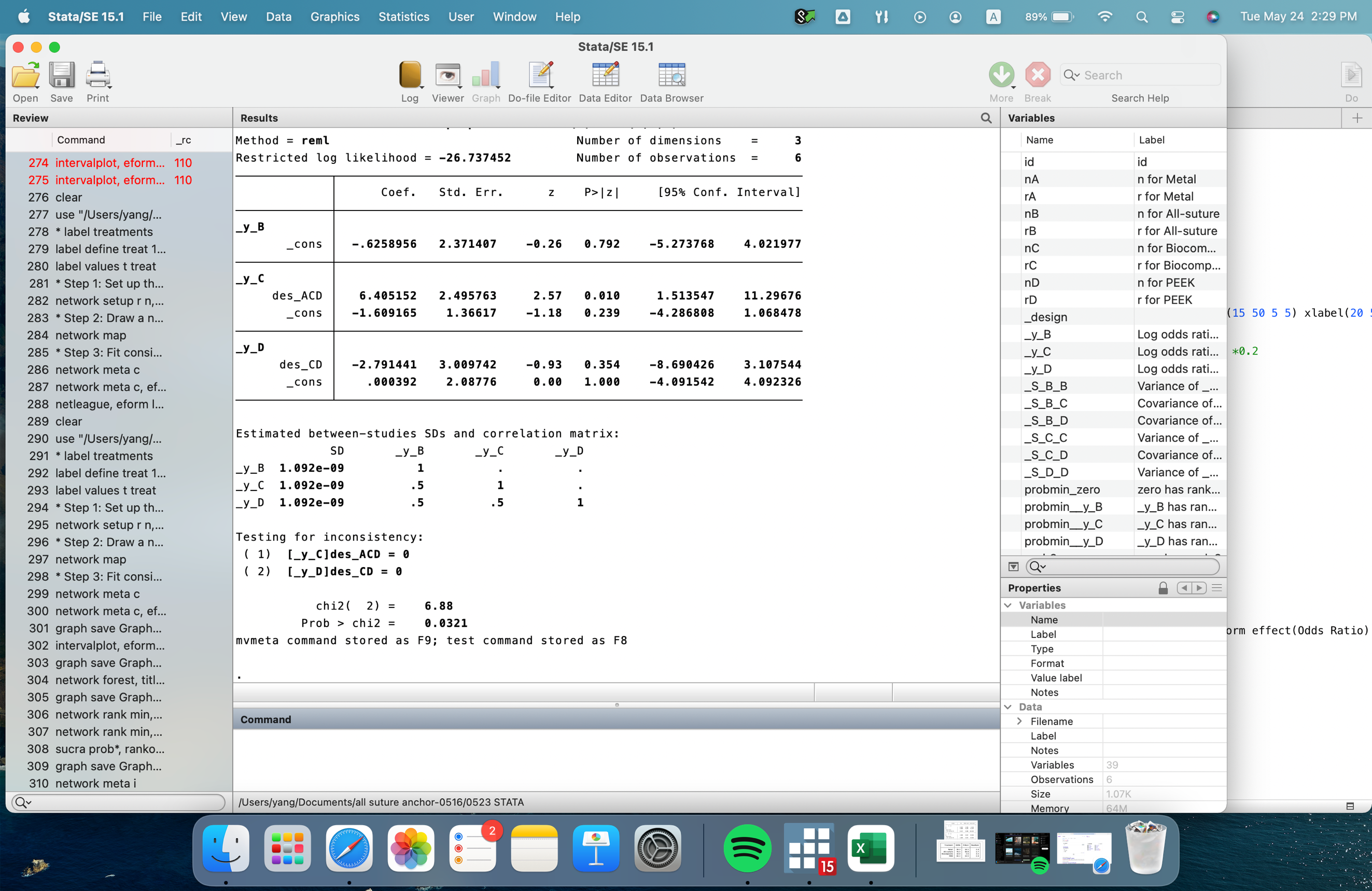


* A: Metal, B: All-suture; C: Biocomposite, D: PEEK.

## 11.6. Inconsistency: Suture Breakage

eTable 11.6.1 Inconsistency between direct and indirect evidence: Suture Breakage

| Side | Direct | | Indirect | | Difference | | |
| --- | --- | --- | --- | --- | --- | --- | --- |
|  | Coef. | Std. Err. | Coef. | Std. Err. | Coef. | Std. Err. | P>z |
| A C | 1.82e-11 | 1.472325 | 0.0046405 | 5.436307 | -0.0046405 | 5.632156 | 0.999 |
| A D | 4.23e-11 | 2.088932 | 0.0018417 | 2.905358 | -0.0018417 | 3.578371 | 1.000 |
| B D | 1.326114 | 1.04232 | 0.0309402 | 109.0142 | 1.295174 | 109.0156 | 0.991 |
| C D | 1.75e-11 | 1.459347 | 0.004708 | 5.492022 | -0.004708 | 5.682604 | 0.999 |

*A: Metal, B: All-suture; C: Biocomposite, D: PEEK.

eTable 11.6.2 Design inconsistency: Suture Breakage


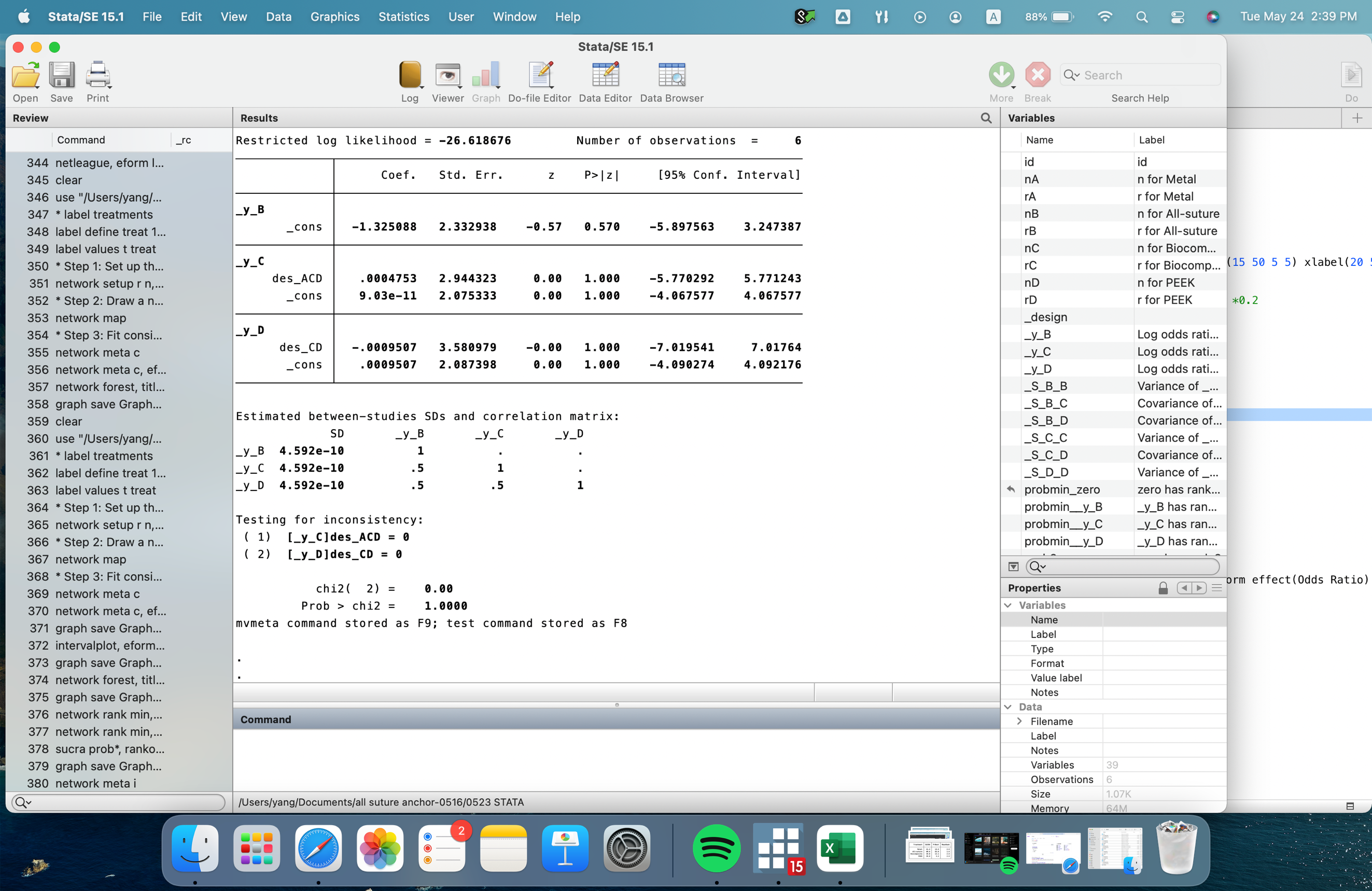


* A: Metal, B: All-suture; C: Biocomposite, D: PEEK.

# Appendix 12: Meta-regression

## 12.1 SUCRA and mean ranks changes before and after model adjustments: Load to Failure

eTable 12.1 SUCRA and mean ranks changes in Load to Failure

| Covariate/ SUCRA | Metal | All-suture | Biocomposite | PEEK |
| --- | --- | --- | --- | --- |
| Unadjusted model | 38.7 | 76.5 | 3.0 | 81.9 |
| Adjusted model | 10.8 | 58.0 | 39.7 | 91.5 |

## 12.2 SUCRA and mean ranks changes before and after model adjustments: Stiffness

eTable 12.2 SUCRA and mean ranks changes in Stiffness

| Covariate/ SUCRA | Metal | All-suture | Biocomposite | PEEK |
| --- | --- | --- | --- | --- |
| Unadjusted model | 71.5 | 79.1 | 33.3 | 16.1 |
| Adjusted model | 83.6 | 16.4 | N/A | N/A |

## 12.3 SUCRA and mean ranks changes before and after model adjustments: Displacement

eTable 12.3 SUCRA and mean ranks changes in LEAST Displacement

| Covariate/ SUCRA | Metal | All-suture | Biocomposite | PEEK |
| --- | --- | --- | --- | --- |
| Unadjusted model | 99.8 | 50.2 | 42.3 | 7.7 |
| Adjusted model | 61.0 | 66.3 | 35.5 | 37.2 |

## 12.4 SUCRA and mean ranks changes before and after model adjustments: Anchor Pullout

eTable 12.4 SUCRA and mean ranks changes in LEAST Anchor pullout

| Covariate/ SUCRA | Metal | All-suture | Biocomposite | PEEK |
| --- | --- | --- | --- | --- |
| Unadjusted model | 63.3 | 12.0 | 83.9 | 40.9 |
| Adjusted model | 93.3 | 4.4 | 65.0 | 37.3 |

## 12.5 SUCRA and mean ranks changes before and after model adjustments: Eyelet Breakage

eTable 12.5 SUCRA and mean ranks changes in LEAST Eyelet breakage

| Covariate/ SUCRA | Metal | All-suture | Biocomposite | PEEK |
| --- | --- | --- | --- | --- |
| Unadjusted model | 39.3 | 76.5 | 18.0 | 66.2 |
| Adjusted model | 9.6 | 82.3 | 42.8 | 65.3 |

## 12.6 SUCRA and mean ranks changes before and after model adjustments: Suture Breakage

eTable 12.6 SUCRA and mean ranks changes in LEAST Suture breakage

| Covariate/ SUCRA | Metal | All-suture | Biocomposite | PEEK |
| --- | --- | --- | --- | --- |
| Unadjusted model | 41.4 | 80.6 | 39.0 | 39.0 |
| Adjusted model | 43.8 | 75.3 | 43.1 | 37.9 |

# Appendix 13: Grading the evidence using CINeMA web application

## 13.1 Confidence Rating: Load to Failure

eTable 13.1. Grading the evidence in Load to failure

| **Comparison** | **Studies** | **Within-study bias** | **Reporting bias** | **Indirectness** | **Imprecision** | **Heterogeneity** | **Incoherence** | **Confidence rating** |
| --- | --- | --- | --- | --- | --- | --- | --- | --- |
| **All-suture: Metal** | 1 | Some concerns | No concerns | No concerns | No concerns | Undetected | Undetected | **Low** |
| **Biosomposite: Metal** | 3 | Some concerns | Major concerns | No concerns | Major concerns | Undetected | Undetected | **Very low** |
| **PEEK: Metal** | 1 | Major concerns | No concerns | No concerns | No concerns | Undetected | Undetected | **Very low** |
| **Biocomposite: All-suture** | 1 | Major concerns | No concerns | Major concerns | No concerns | Undetected | Undetected | **Very low** |
| **PEEK: All-suture** | 3 | Some concerns | No concerns | No concerns | Major concerns | Undetected | Undetected | **Very low** |
| **PEEK: Biocomposite** | 2 | Major concerns | No concerns | Major concerns | Some concerns | Undetected | Undetected | **Very low** |

## 13.2 Confidence Rating: Stiffness

eTable 13.2. Grading the evidence in Stiffness

| **Comparison** | **Studies** | **Within-study bias** | **Reporting bias** | **Indirectness** | **Imprecision** | **Heterogeneity** | **Incoherence** | **Confidence rating** |
| --- | --- | --- | --- | --- | --- | --- | --- | --- |
| **All-suture: Metal** | 1 | No concerns | No concerns | No concerns | No concerns | Undetected | Undetected | **Low** |
| **Biosomposite: Metal** | 1 | No concerns | Some concerns | No concerns | No concerns | Undetected | Undetected | **Low** |
| **PEEK: Metal** | 0 | No concerns | No concerns | Some concerns | Some concerns | Undetected | Undetected | **Low** |
| **Biocomposite: All-suture** | 1 | No concerns | No concerns | No concerns | No concerns | Undetected | Undetected | **High** |
| **PEEK: All-suture** | 2 | Some concerns | No concerns | No concerns | Major concerns | Undetected | Undetected | **Very low** |
| **PEEK: Biocomposite** | 0 | No concerns | No concerns | Some concerns | No concerns | Undetected | Undetected | **Low** |

## 13.3 Confidence Rating: Displacement

eTable 13.3. Grading the evidence in Displacement

| **Comparison** | **Studies** | **Within-study bias** | **Reporting bias** | **Indirectness** | **Imprecision** | **Heterogeneity** | **Incoherence** | **Confidence rating** |
| --- | --- | --- | --- | --- | --- | --- | --- | --- |
| **All-suture: Metal** | 1 | Major concerns | No concerns | Some concerns | No concerns | Undetected | Undetected | **Very low** |
| **Biosomposite: Metal** | 2 | Major concerns | No concerns | Some concerns | Some concerns | Undetected | Undetected | **Very low** |
| **PEEK: Metal** | 0 | No concerns | No concerns | Some concerns | No concerns | Undetected | Undetected | **Low** |
| **Biocomposite: All-suture** | 1 | No concerns | No concerns | No concerns | Major concerns | Undetected | Undetected | **Very low** |
| **PEEK: All-suture** | 3 | Some concerns | No concerns | No concerns | Major concerns | Undetected | Undetected | **Very low** |
| **PEEK: Biocomposite** | 0 | No concerns | No concerns | No concerns | No concerns | Undetected | Undetected | **High** |

## 13.4 Confidence Rating: Failure Modes

eTable 13.4. Grading the evidence in Anchor Pullout

| **Comparison** | **Studies** | **Within-study bias** | **Reporting bias** | **Indirectness** | **Imprecision** | **Heterogeneity** | **Incoherence** | **Confidence rating** |
| --- | --- | --- | --- | --- | --- | --- | --- | --- |
| **All-suture: Metal** | 0 | No concerns | No concerns | Major concerns | Some concerns | Some concerns | No concerns | **Very low** |
| **Biosomposite: Metal** | 2 | Some concerns | Some concerns | No concerns | Major concerns | Major concerns | Some concerns | **Very low** |
| **PEEK: Metal** | 1 | No concerns | No concerns | No concerns | Major concerns | Major concerns | Some concerns | **Very low** |
| **Biocomposite: All-suture** | 0 | No concerns | No concerns | No concerns | No concerns | Major concerns | No concerns | **Very low** |
| **PEEK: All-suture** | 3 | Some concerns | No concerns | Some concerns | No concerns | Some concerns | No concerns | **Low** |
| **PEEK: Biocomposite** | 2 | Some concerns | No concerns | Some concerns | No concerns | No concerns | Some concerns | **Low** |

eTable 13.5. Grading the evidence in Eyelet Breakage

| **Comparison** | **Studies** | **Within-study bias** | **Reporting bias** | **Indirectness** | **Imprecision** | **Heterogeneity** | **Incoherence** | **Confidence rating** |
| --- | --- | --- | --- | --- | --- | --- | --- | --- |
| **All-suture: Metal** | 0 | No concerns | No concerns | Some concerns | Some concerns | Major concerns | No concerns | **Very low** |
| **Biosomposite: Metal** | 2 | Some concerns | Some concerns | No concerns | Major concerns | Major concerns | Some concerns | **Very low** |
| **PEEK: Metal** | 1 | No concerns | No concerns | No concerns | Major concerns | Major concerns | Some concerns | **Very low** |
| **Biocomposite: All-suture** | 0 | No concerns | No concerns | Some concerns | No concerns | No concerns | No concerns | **Low** |
| **PEEK: All-suture** | 3 | No concerns | No concerns | No concerns | No concerns | Some concerns | No concerns | **Low** |
| **PEEK: Biocomposite** | 2 | Some concerns | No concerns | No concerns | No concerns | Major concerns | Some concerns | **Very low** |

eTable 13.6. Grading the evidence in Suture Breakage

| **Comparison** | **Studies** | **Within-study bias** | **Reporting bias** | **Indirectness** | **Imprecision** | **Heterogeneity** | **Incoherence** | **Confidence rating** |
| --- | --- | --- | --- | --- | --- | --- | --- | --- |
| **All-suture: Metal** | 0 | No concerns | No concerns | Some concerns | Some concerns | Major concerns | Major concerns | **Very low** |
| **Biosomposite: Metal** | 2 | No concerns | No concerns | No concerns | Major concerns | Major concerns | Some concerns | **Very low** |
| **PEEK: Metal** | 1 | No concerns | Some concerns | No concerns | Major concerns | Major concerns | Some concerns | **Very low** |
| **Biocomposite: All-suture** | 0 | No concerns | No concerns | No concerns | Some concerns | Some concerns | No concerns | **Low** |
| **PEEK: All-suture** | 3 | Some concerns | No concerns | Some concerns | No concerns | Some concerns | No concerns | **Low** |
| **PEEK: Biocomposite** | 2 | No concerns | No concerns | No concerns | Major concerns | Major concerns | Some concerns | **Very low** |

# Appendix 14: Sensitivity analysis

## 14.1 League table excluding trials of artificial bone: Load to Failure

eTable 14.1. Load to Failure in league table, exclusion of trials utilizing artificial bone models

| **Metal** | 58.13  (-43.44, 159.71) | 20.98  (-15.90, 57.86) | 80.85  (-14.21, 175.91) |
| --- | --- | --- | --- |
| -51.60  (-138.34, 34.97) | **All-suture** | -37.15  (-131.83, 57.52) | 22.72  (-13.09, 58.53) |
| 49.75  (-17.24, 116.74) | 101.44 (19.13, 183.74) | **Biocomposite** | 59.88  (-27.77, 147.52) |
| -56.25  (-137.46, 24.97) | -4.56  (-71.91, 62.78) | -106.00  (-180.33, -31.66) | **PEEK** |

*Results of the network meta-analysis are presented in the lower left triangle. Sensitivity analysis with exclusion of trials utilizing synthetic bone models are presented in the upper right triangle.

Odd ratio and 95% confidence interval were presented and left hand side intervention was reference group. Odds ratio less than one favor the column-defining treatment.

## 14.2 League table excluding trials of artificial bone: Stiffness

eTable 14.2. Stiffness in league table, exclusion of trials utilizing artificial bone models

| **Metal** | - | - | - |
| --- | --- | --- | --- |
| -0.02  (-0.20, 0.16) | **All-suture** | - | - |
| 0.08  (-0.05, 0.21) | 0.10 (-0.03, 0.23) | **Biocomposite** | - |
| -2.63  (-2.48, 7.75) | 2.65  (-2.46, 7.76) | 2.55  (-2.56, 7.66) | **PEEK** |

*Results of the network meta-analysis are presented in the lower left triangle. Sensitivity analysis with exclusion of trials utilizing synthetic bone models are presented in the upper right triangle.

Odd ratio and 95% confidence interval were presented and left hand side intervention was reference group. Odds ratio less than one favor the column-defining treatment.

## 14.3 League table excluding trials of artificial bone: Displacement

eTable 14.3. Displacement in league table, exclusion of trials utilizing artificial bone models

| **Metal** | -0.80  (-68.97, 67.37) | 1.41  (-1.23, 4.05) | 0.18  (-67.99, 68.35) |
| --- | --- | --- | --- |
| -1.59  (-2.72, -0.46) | **All-suture** | 2.21  (-66.01, 70.43) | 0.98  (-0.22, 2.19) |
| -1.67  (-2.47, -0.88) | -0.09 (-1.25, 1.08) | **Biocomposite** | -1.23  (-69.45, 66.99) |
| -2.57  (-4.22, -0.92) | -0.98  (-2.19, 0.22) | 0.90  (-2.57, 0.78) | **PEEK** |

*Results of the network meta-analysis are presented in the lower left triangle. Sensitivity analysis with exclusion of trials utilizing synthetic bone models are presented in the upper right triangle.

Odd ratio and 95% confidence interval were presented and left hand side intervention was reference group. Odds ratio less than one favor the column-defining treatment.

## 14.4 League table excluding trials of artificial bone: Anchor Pullout

eTable 14.4. Anchor pullout in league table, exclusion of trials utilizing artificial bone models

| **Metal** | 147.08  (1.46, 14819.32) | 4.98  (0.34, 72.44) | 29.54  (0.48, 1831.53) |
| --- | --- | --- | --- |
| 0.05  (0.00, 8.71) | **All-suture** | 0.03  (0.00, 1.45) | 0.20  (0.03, 1.58) |
| 2.33  (0.07, 74.33) | 47.13 (0.49, 4534.58) | **Biocomposite** | 5.93  (0.26, 137.47) |
| 0.23  (0.00, 16.03) | 4.69  (0.24, 89.96) | 0.10  (0.00, 3.27) | **PEEK** |

*Results of the network meta-analysis are presented in the lower left triangle. Sensitivity analysis with exclusion of trials utilizing synthetic bone models are presented in the upper right triangle.

Odd ratio and 95% confidence interval were presented and left hand side intervention was reference group. Odds ratio less than one favor the column-defining treatment.

## 14.5 League table excluding trials of artificial bone: Eyelet Breakage

eTable 14.5. Eyelet breakage in league table, exclusion of trials utilizing artificial bone models

| **Metal** | 0.03  (0.00, 3.94) | 0.20  (0.01, 2.91) | 0.06  (0.00, 4.30) |
| --- | --- | --- | --- |
| 6.27  (0.04, 996.13) | **All-suture** | 6.08  (0.12, 321.36) | 1.87  (0.21, 16.97) |
| 0.48  (0.02, 13.69) | 0.08 (0.00, 6.94) | **Biocomposite** | 0.31  (0.01, 8.32) |
| 3.53  (0.06, 214.92) | 0.56  (0.03, 10.77) | 7.34  (0.24, 222.86) | **PEEK** |

*Results of the network meta-analysis are presented in the lower left triangle. Sensitivity analysis with exclusion of trials utilizing synthetic bone models are presented in the upper right triangle.

Odd ratio and 95% confidence interval were presented and left hand side intervention was reference group. Odds ratio less than one favor the column-defining treatment.

## 14.6 League table excluding trials of artificial bone: Suture Breakage

eTable 14.6. Suture breakage in league table, exclusion of trials utilizing artificial bone models

| **Metal** | 0.27  (0.00, 112.76) | 1.00  (0.02, 58.30) | 1.00  (0.00, 297.85) |
| --- | --- | --- | --- |
| 3.76  (0.08, 186.20) | **All-suture** | 3.76  (0.04, 334.11) | 3.77  (0.49, 29.05) |
| 1.00  (0.06, 16.20) | 0.27 (0.01, 8.26) | **Biocomposite** | 1.00  (0.02, 54.36) |
| 1.00  (0.04, 27.76) | 0.27  (0.03, 2.05) | 1.00  (0.06, 15.86) | **PEEK** |

*Results of the network meta-analysis are presented in the lower left triangle. Sensitivity analysis with exclusion of trials utilizing synthetic bone models are presented in the upper right triangle.

Odd ratio and 95% confidence interval were presented and left hand side intervention was reference group. Odds ratio less than one favor the column-defining treatment.

# Appendix 15: The Quality Appraisal for Cadaveric Studies (QUACS) scale

## 15.1 The Tables of The Quality Appraisal for Cadaveric Studies (QUACS) scale

eTable 15.1. Tables of The Quality Appraisal for Cadaveric Studies (QUACS) scale

| Item/ Study | Ntalos et al. 2019 | Ntalos et al. 2019 | Nagra et al. 2017 | Barber et al. 2010 | Pietshmann et al. 2009 |
| --- | --- | --- | --- | --- | --- |
| 1. Objective stated | Yes | Yes | Yes | Yes | No |
| 2. Basic information about sample is included | No | No | Yes | Yes | Yes |
| 3. Applied methods are described comprehensibly | Yes | Yes | Yes | Yes | Yes |
| 4. Study reports condition of the examined specimens | No | No | No | No | No |
| 5. Education of dissecting researchers is stated | No | No | No | No | No |
| 6. Findings are observed by more than one researcher | No | No | No | No | No |
| 7. Results presented thoroughly and precise | Yes | Yes | Yes | Yes | Yes |
| 8. Statistical methods appropriate | Yes | Yes | Yes | Yes | Yes |
| 9. Details about consistency of findings are given | Yes | Yes | Yes | Yes | Yes |
| 10. Photographs of the observations are included | No | Yes | Yes | No | No |
| 11. Study is discussed within the context of the current evidence | Yes | Yes | Yes | Yes | Yes |
| 12. Clinical implications of the results are discussed | Yes | Yes | Yes | Yes | Yes |
| 13. Limitations of the study are addressed | Yes | Yes | Yes | Yes | Yes |
| Total score | **8** | **9** | **10** | **9** | **8** |
